# Highly diastereoselective construction of novel dispiropyrrolo[2,1-*a*]isoquinoline derivatives *via* multicomponent 1,3-dipolar cycloaddition of cyclic diketones-based tetrahydroisoquinolinium *N*-ylides†

**DOI:** 10.1039/c8ra09884k

**Published:** 2019-04-09

**Authors:** Sarra Boudriga, Saoussen Haddad, Moheddine Askri, Armand Soldera, Michael Knorr, Carsten Strohmann, Christopher Golz

**Affiliations:** Laboratory of Heterocyclic Chemistry Natural Product and Reactivity/CHPNR, Department of Chemistry, Faculty of Science of Monastir 5000 Monastir Tunisia sarra_boudriga@yahoo.fr; Departement of Chemistry, Quebec Center for Functional Materials University of Sherbrooke Sherbrooke Quebec Canada J1K 2R1; Institut UTINAM - UMR CNRS 6213, Université Bourgogne Franche-Comté 16 Route de Gray 25030 Besançon France; Technische Universität Dortmund, Anorganische Chemie Otto-Hahn-Strasse 6 44221 Dortmund Germany

## Abstract

In the quest for new heterocyclic scaffolds exhibiting potentially biological activities for medicinal chemistry, a multicomponent 1,3-dipolar cycloaddition reaction of tetrahydroisoquinolinium *N*-ylides, generated *in situ* from cyclic diketones and isoquinoline, and (*E*)-3-arylidene-1-phenyl-pyrrolidine-2,5-diones has been developed. This route provides workable access to dispiropyrrolo[2,1-*a*]isoquinoline-fused pyrrolidine-2,5-diones bearing two adjacent spiro-carbons. An unprecedented regioselectivity was observed in this 1,3-dipolar cycloaddition, leading to the construction of a novel dispirooxindole skeleton. The structure and relative stereochemistry of the spiranic adducts have been confirmed by three X-ray diffraction studies. To reinforce the observed regio- and stereoselectivity of the [3+2] cycloaddition, calculations using the DFT approach at the B3LYP/6-31G(d,p) level were carried out. It was found that this reaction affords the kinetic products.

## Abstract 2

A new series of dispiropyrrolo[2,1-*a*]isoquinoline derivatives was synthesized by multicomponent 1,3-dipolar cycloaddition reaction of tetrahydroisoquinolinium *N*-ylides and (*E*)-3-arylidene-1-phenyl-pyrrolidine-2,5-diones.

## Graphical abstract

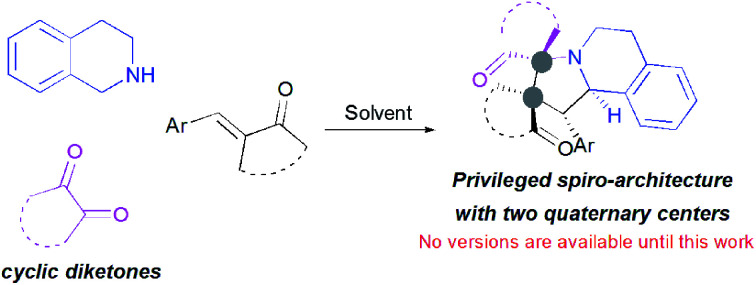

## Introduction

**Fig. 1:**
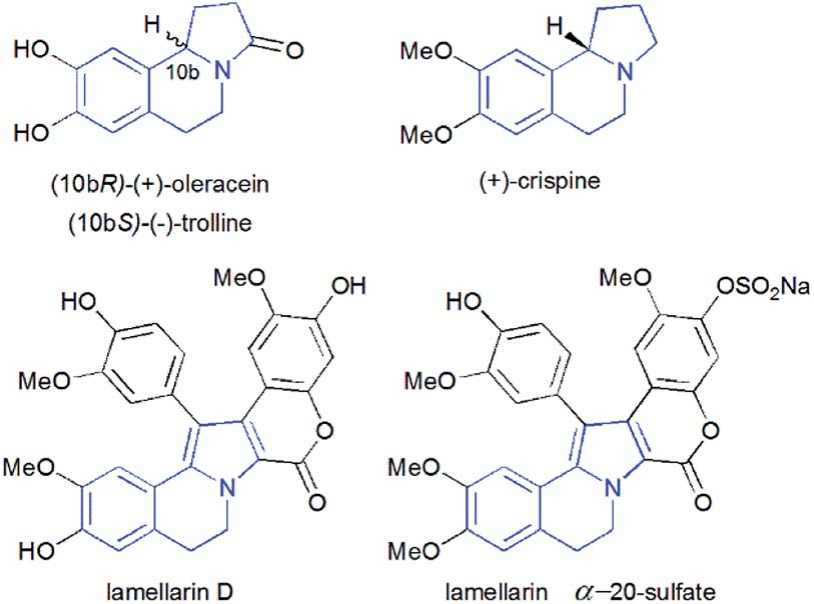
Structures of some pyrrolo[2,1-*a*]isoquinoline alkaloids.

Pyrrolo[2,1-*a*]isoquinolines as structural motifs have drawn considerable interest in the area of synthetic organic chemistry and medicinal chemistry as they represent substructures often encountered in numerous bioactive natural isolates and pharmaceutically important compounds.^1^ Some illustrative examples are depicted in Fig. 1 including (+)-oleracein,^1*b*^ (−)-trolline,^1*c*^ (+)-crispine^1*d*^ and lamellarin alkaloids such as lamellarin D and lamellarin α-20-sulfate.^1*f*^ Most of the pyrrolo[2,1-*a*]isoquinoline alkaloids display remarkable antitumor,^2^ antibacterial and antiviral,^3^ anti-inflammatory,^4^ antidepressant^5^ and cardiovascular^6^ properties. Furthermore, lamellarin D, isolated from marine molluscs, has been reported to be an inhibitor of human topoisomerase,^7^ whereas lamellarin α-20-sulfate inhibits the enzyme HIV integrase.^1*e*,8^ Owing to the intriguing synthetic value and multiple biological activities of these heterocyclic systems, development of efficient and versatile synthetic methods allowing the construction of such scaffolds was strongly encouraged.

**Scheme 1:**
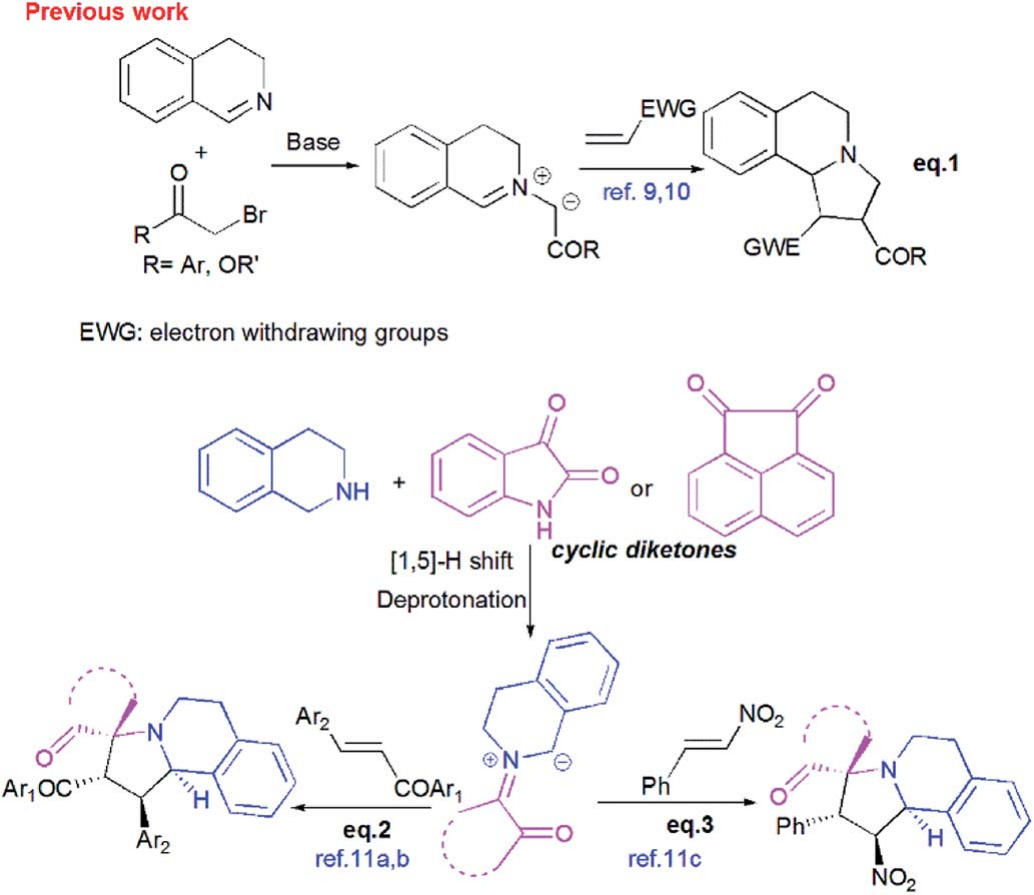
Strategy for direct construction of spiropyrrolo[2,1-*a*]isoquinoline derivatives.

In the past decade, 1,3-dipolar cycloaddition reactions of isoquinolinium *N*-ylides with activated alkynes or olefins^9^ have arguably emerged as a fascinating and powerful tool for the synthesis of pyrrolo[2,1-*a*]isoquinoline derivatives. However, a survey of the literature reveals that the reported 1,3-dipoles were generated *in situ* by basic deprotonation of their corresponding isoquinolinium bromides (Scheme 1, eqn (1)).^9,10^ These latter were prepared mainly by the condensation of isoquinoline with substituted phenacyl bromide or alkylbromoacetates.

However, tetrahydroisoquinolinium *N*-ylide stemming from cyclic diketones have rarely been considered as azomethine ylide precursors.

So far, Sarrafi *et al.*^11^ developed the first examples of multicomponent 1,3-dipolar cycloaddition reaction involving acenaphthenequinone and isatin as cyclic diketones-based tetrahydroisoquinolinium *N*-ylides with chalcone and nitrostyrene derivatives as dipolarophiles (Scheme 1, eqn 2 and 3).^11^ The azomethine ylides were produced *in situ via* [1,5]-H shift and subsequent deprotonation.

**Scheme 2:**
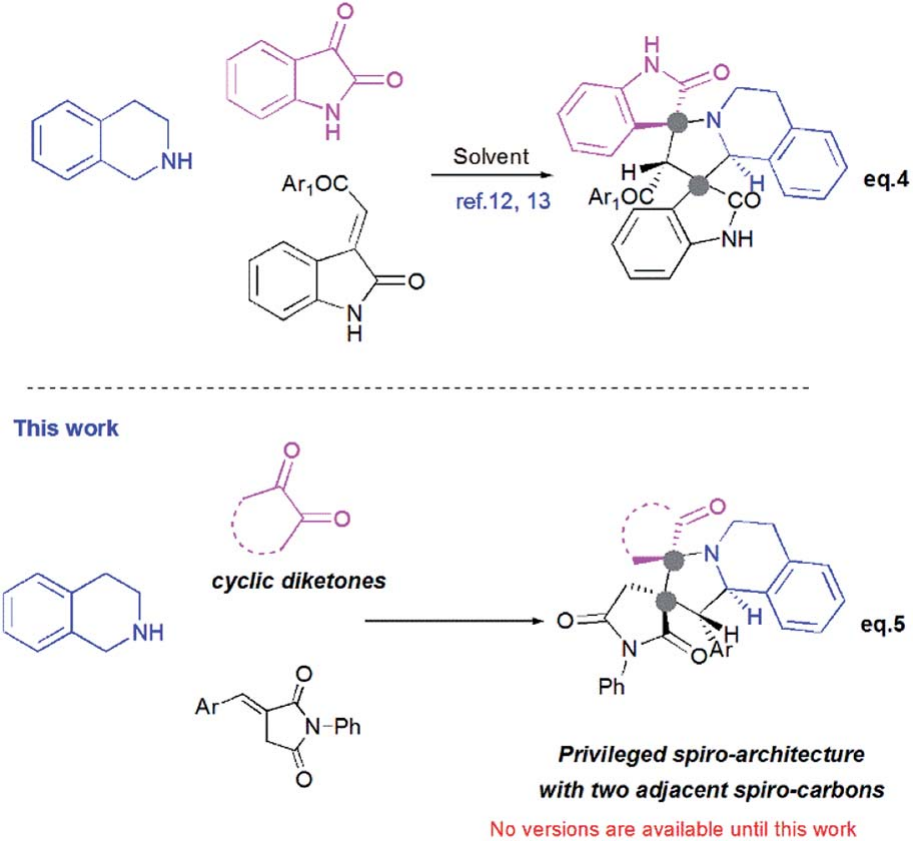
Profile of 1,3-dipolar cycloaddition for exocyclic enones with tetrahydroisoquinolinium *N*-ylides derived from cyclic diketones.

Recently, Huang^12^ and Kumar^13^ successfully developed a [3+2]-cycloaddition reaction of 2-oxoindolin-3-ylidene derivatives with azomethine ylides derived from isatin and 1,2,3,4-tetrahydroisoquinoline, and only one single regioisomer, namely dispiroindoline-3,1′-pyrrolo[2,1-*a*]isoquinoline-3′,3′-indoline-2,2′′-dione was obtained. Generally, it has been found that dispiropyrrolo[2,1-*a*]isoquinoline derivatives were selectively formed in this kind of 1,3-dipolar cycloadditions. However, the alternative regioisomer incorporating two adjacent spiro-carbons, has never been reported (Scheme 2, eqn (4)).^12–14^ In spite of this initial work, the systematic investigation on 1,3-dipolar cycloaddition involving exocyclic enones and tetrahydroisoquinolinium *N*-ylides derived from cyclic diketones is still rather limited and represents certainly interesting opportunities for further development.

New advances in this area could afford a convenient approach to structurally novel pyrrolo[2,1-*a*]isoquinoline derivatives bearing multiple neighboured stereogenic centres, which may provide some benefits to medicinal chemistry and drug discovery. Considering this challenge, we wondered whether α-alkylidenesuccinimides could be employed as exocyclic enones to undergo [3+2] cycloaddition using tetrahydroisoquinolinium *N*-ylides derived from cyclic diketones, thus yielding dispiropyrrolo[2,1-*a*]isoquinoline derivatives.

On the other hand, our targeted formal 1,3-dipolar cycloaddition products contain pyrrolidinone-2,5-dione scaffolds. These latter have the important advantage of being present in a great variety of natural products as well as in a vast library of synthetic compounds endowed with several bioactivities.^15^ Recently, we reported the synthesis of functionalized spirooxindolepyrrolidines and dispiropyrrolothiazoles containing the pyrrolidine-2,5-dione motif which exhibits antibacterial, antifungal, antimalarial, and antimycobacterial activities.^16^

Herein, and in continuation of our research in the area of [3+2] cycloaddition of azomethine ylides,^16,17^ we describe for the first time the formation of the hitherto unknown regioisomer, with two adjacent spiro-carbons, by 1,3-dipolar cycloaddition reactions of cyclic diketones-based tetrahydroisoquinolinium *N*-ylides and α-alkylidenesuccinimides. This route provides an attractive access to novel dispiropyrrolo[2,1-*a*]isoquinolines with a broad substrate scope in good to high yields. The stereochemistry of the spiroadducts has been confirmed by three X-ray diffraction studies. Moreover, to better rationalize mechanistically this kind of reaction, the regio- and stereochemistry of dispiropyrrolo[2,1-*a*]isoquinoline was investigated by means of Density Functional Theory (DFT) calculations.

## Results and discussion

### Three-component synthesis of the spiropyrrolo[2,1-*a*]isoquinolines

**Scheme 3:**
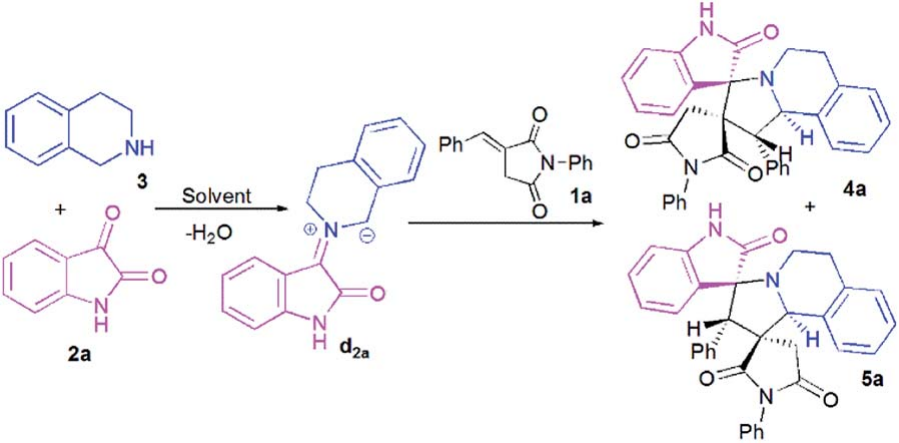
Reaction of (*E*)-3-benzylidene-1-phenylpyrrolidine-2,5-dione 1a with isatin 2a and 1,2,3,4-tetrahydroisoquinoline (THIQ) 3.

At the onset of our work, (*E*)-3-benzylidene-1-phenylpyrrolidine-2,5-dione 1a, isatin 2a and 1,2,3,4-tetrahydroisoquinoline (THIQ) 3 were employed as substrate models to optimize the reaction conditions (Scheme 3, Table 1). The reaction was investigated with different solvents of different polarities such as methanol, ethanol, acetonitrile and toluene.

**Table 1:** Optimization of the 1,3-dipolar cycloaddition of dipolarophile 1a with isatin 2a and 1,2,3,4-tetrahydroisoquinoline (THIQ) 3a^a^

Entry	Solvent	*T* (°C)	Time (h)	rr^c^ (4 : 5)	Yield^d^ (%)
1	PhCH_3_	25	24	—	NR^b^
2	MeOH	25	24	—	NR^b^
3	EtOH	25	24	—	NR^b^
4	CH_3_CN	25	24	—	NR^b^
5	PhCH_3_	Reflux	12	—	Trace
6	CH_3_CN	Reflux	6	52 : 48	55
7	EtOH	Reflux	4	60 : 40	65
**8**	**MeOH**	**Reflux**	**4**	**60** **:** **40**	**85**

a The reaction was carried out in 0.2 mmol scale in solvent (2 mL), and the ratio of 1a/2a/3 is 1 : 1 : 1.2.

b No reaction due to insufficient solubility.

c The regioisomeric ratio (rr) was determined by ^1^H NMR spectra of the crude reaction mixture.

d Combined yield of isolated 4a and 5a.

As shown in Table 1, the reaction does not proceed at room temperature (Table 1, entries 1–4) and does not occur in refluxing toluene (Table 1, entry 5). Surprisingly, in acetonitrile, the reaction proceeds smoothly affording the corresponding spiropyrrolo[2,1-*a*]isoquinolines as a mixture of two regioisomers 4a and 5a in a 60 : 40 ratio, albeit with a moderate yield (Table 1, entry 6). As can be seen from Table 1, the best results were obtained by refluxing the reaction mixture in methanol for 4 h, providing isomeric dispiropyrrolo[2,1-*a*]isoquinolineoxindoles 4a and 5a in high yield (85%), along with high diastereoselectivities (Table 1, entry 8).

**Scheme 4:**
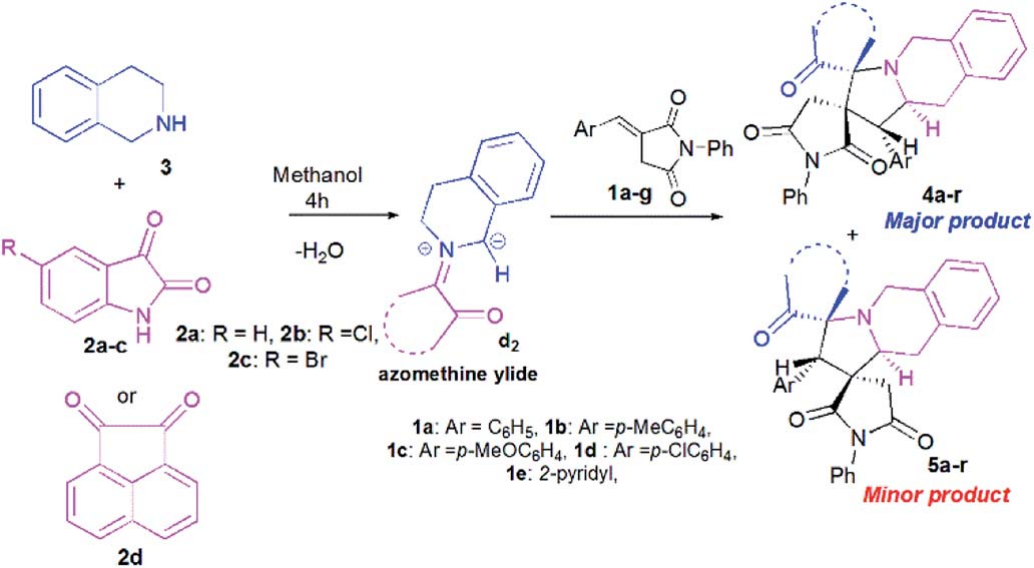
Reaction of (*E*)-3-arylidene-1-phenylpyrrolidine-2,5-dione 1a–g with cyclic diketones 2 and 1,2,3,4-tetrahydroisoquinoline (THIQ) 3.

Once the optimal conditions being established (Table 1, entry 8), we tried to extend the scope of this reaction with different *p*-aryl substituted dipolarophiles 1, as well as with various diketones 2a–d (Scheme 4, Table 2). The electronic properties exerted by the substituent at the *p*-position of the aryl group of imides 1 were shown to have little influence on the efficiency of this reaction (Table 2). For example, enones 1 bearing an electron-neutral (H), or electron-donating (*e.g.*, 4-Me or 4-OMe) group or electron-withdrawing substituent (Cl) or group (2-pyridinyl) reacted smoothly to give isomeric spiropyrrolo[2,1-*a*]isoquinolineoxindoles products in excellent yields (Table 2, entries 1–5).

**Table 2:** Synthesis of the dispiropyrrolo[2,1-*a*]isoquinolines 4 and 5^a^

Entry	Comp.	Diketone	Ar	rr^b^ (4 : 5)	Yield^d^ (%)
1	4a + 5a	2a	C_6_H_5_	60 : 40^c^	85
2	4b + 5b	2a	*p*-MeC_6_H_4_	65 : 35	88
3	4c + 5c	2a	*p*-MeOC_6_H_4_	68 : 32^c^	87
4	4d + 5d	2a	*p*-ClC_6_H_4_	68 : 32^c^	80
5	4e + 5e	2a	2-Pyridyl	60 : 40	82^e^
6	4f + 5f	2b	C_6_H_5_	80 : 20	85
7	4g + 5g	2b	*p*-MeC_6_H_4_	83 : 17	83
8	4h + 5h	2b	*p*-MeOC_6_H_4_	>90 : 10	87^e^
9	4i + 5i	2b	2-Pyridyl	>90 : 10	85^e^
10	4j + 5j	2c	C_6_H_5_	>90 : 10	80^e^
11	4k + 5k	2c	*p*-MeC_6_H_4_	85 : 15	88
12	4l + 5l	2c	*p*-MeOC_6_H_4_	86 : 14	85
13	4m + 5m	2c	2-Pyridyl	>90 : 10	83^e^
14	4n + 5n	2d	C_6_H_5_	77 : 23	72
15	4o + 5o	2d	*p*-MeC_6_H_4_	78 : 22^c^	75^f^
16	4p + 5p	2d	*p*-MeOC_6_H_4_	78 : 22	67
17	4q + 5q	2d	*p*-ClC_6_H_4_	90 : 10	63
18	4r + 5r	2d	2-Pyridyl	80 : 20^c^	65^f^

a The reaction was carried out in 1 mmol scale in methanol (5 mL) at reflux for 4 h, and the ratio of 1/2/3 is 1 : 1 : 1.2.

b Unless otherwise noted, the regioisomeric ratio (rr) was determined by the isolated yields of 4 and 5.

c Determined by ^1^H NMR analysis of the crude product.

d Combined yields of isolated 4 and 5.

e Failed to separate compound 5.

f Failed to separate compound 4.

However, the regioselectivity of these reactions was quite poor. Moreover, it seems that the electronic effects induced by (i) halogen substituents (Cl and Br) on the phenyl ring of isatin (Table 2, entries 6–13) or (ii) acenaphthenequinone 2d, as cyclic diketone (Table 2, entries 14–18), were beneficial for increasing the regioselectivities. All compounds were isolated as colourless solids.

### Spectroscopic and crystallographic characterization of the isomeric cycloadducts

The structure and the relative configuration of the isomeric pyrrolo[2,1-*a*]isoquinolines resulting from the cycloaddition were deduced from NMR in solution and, in the solid state from X-ray structure determinations performed on cycloadducts 4a, 5a and 5r.

**Fig. 2:**
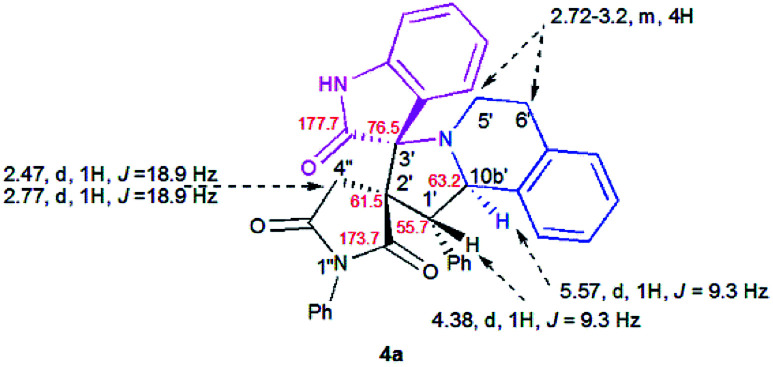
Selected ^1^H and ^13^C NMR chemical shifts of 4a.

**Fig. 3:**
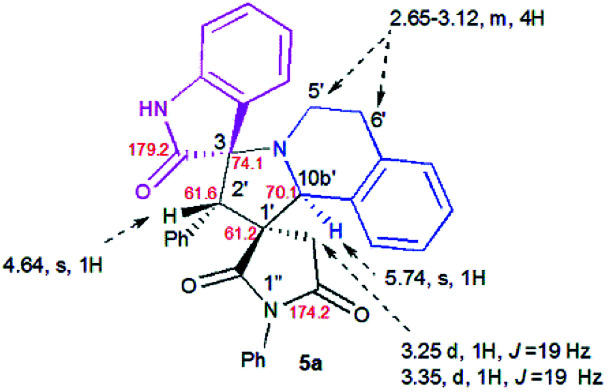
Selected ^1^H and ^13^C NMR chemical shifts of 5a.

Relevant ^1^H and ^13^C chemical shifts of pyrrolo[2,1-*a*]isoquinolines 4a and 5a are shown in Fig. 2 and 3, respectively. The ^1^H NMR spectrum of 4a shows two mutually coupled doublets at *δ* 2.47 and 2.77 ppm (*J* = 18.9 Hz) corresponding to the diastereotopic 4′-CH_2_, as well as two further doublets at *δ* 4.38 (*J* = 9.3 Hz) and 5.57 ppm (*J* = 9.3 Hz) assigned to the pyrrolidine H-1′ and H-10b′ protons (Fig. 2), respectively.

Their coupling constants of approximately 9 Hz indicate that these protons are *trans*-arranged. The occurrence and the multiplicity of these signals clearly prove the regiochemistry of the cycloaddition reaction. In contrast, in the ^1^H NMR spectrum of regioisomer 5a (Fig. 3), the pyrrolidinyl protons H-2′ and H-10b′ appear as two singlets resonating at *δ* 4.64 and 5.74 ppm, respectively. The ^13^C NMR spectra of all synthesized spirooxindoles exhibit two peaks at*δ* 74.1–76.5 and 177.7–179.2 ppm for the spiro-carbon and the oxindole carbonyl group, respectively.

**Fig. 4:**
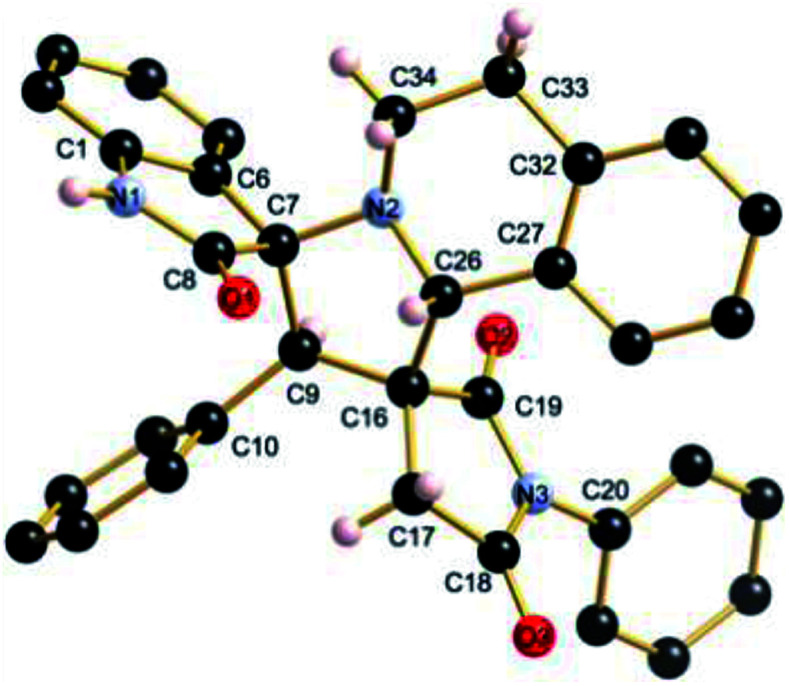
Ball and sticks presentation of the molecular structure of 4a in the crystal. For clarity, only stereochemically significant hydrogen atoms are shown. Selected bond lengths (Å) and angles (°): C4–N8 1.3991(16), C10–N8 1.3992(16), C10–O3 1.2015(17), C4–O1 1.2012(16), C19–N5 1.4506(18), C27–N5 1.4582(17), C15–N7 1.3441(18), C15–O2 1.2295(16); C4–N8–C10 112.14(11), C27–N5–C20 114.71(11), N5–C20–C36 105.63(12), C19–C16–C25 104.23(10), C25–C27–N5 101.17(10), C27–N5–C19 107.04(11), N5–C19–C15 111.46(11).

**Fig. 5:**
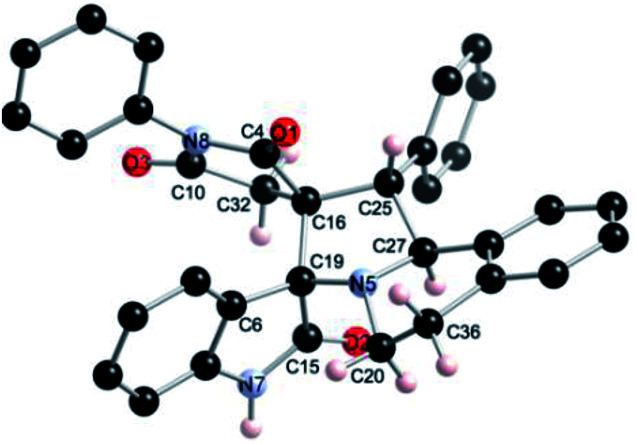
Ball and sticks presentation of the molecular structure of 5a in the crystal. For clarity, only stereochemically significant hydrogen atoms are shown. Selected bond lengths (Å) and angles (°): C8–O1 1.224(4), C8–N1 1.366(4), N1–C1 1.409(4), C19–O2 1.207(3), C19–N3 1.401(4), N3–C18 1.386(4), C18–O3 1.220(3), N2–C26 1.471(4), N2–C34 1.460(4); C1–N1–C8 112.2(3), C8–C7–N2 112.6(2), C7–N2–C34 116.9(2), C7–N2–C26 105.9(2), C16–C26–N2 103.0(2), C27–C26–N2 110.3(2), C19–N3–C18 112.3(2), N3–C18–C17 108.6(3), N3–C19–C16 108.8(2).

**Fig. 6:**
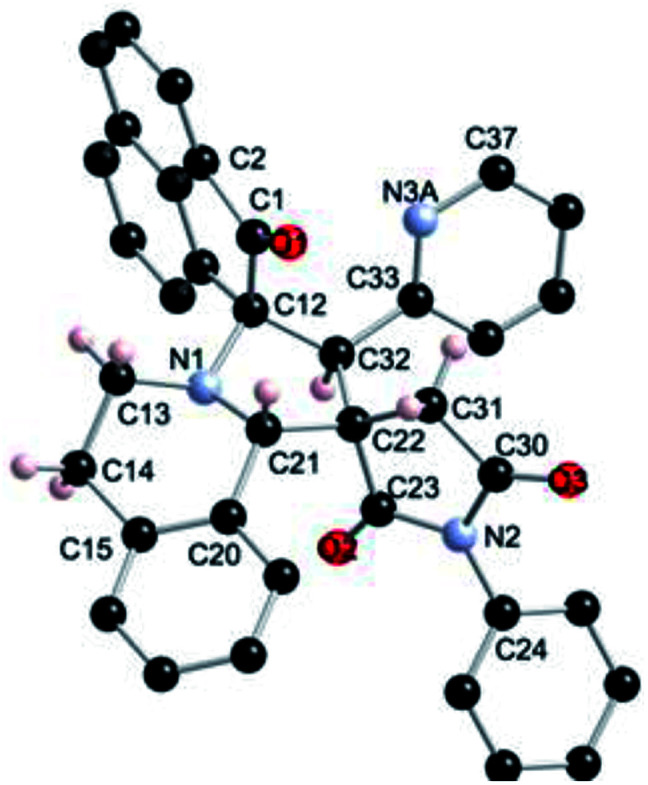
Ball and sticks presentation of the molecular structure of 5r in the crystal. For clarity, only stereochemically significant hydrogen atoms are shown. Selected bond lengths (Å) and angles (°): C1–O1 1.2134(17), C12–N1 1.4760(18), N1–C21 1.4577(17), C23–O2 1.2086(17), C23–N2 1.3935(17), N2–C30 1.3975(17), C30–O3 1.2107(16); C12–N1–C21 106.09(10), N1–C21–C22 103.57(11), C21–C22–C23 112.90(11), C32–C12–N1 98.54(10), C22–C23–N2 108.53(11), C23–N2–C30 112.73(11), N2–C30–C31 108.11(11), C31–C22–C32 115.82(11).

The regio- and the stereochemical outcome of the cycloadditions were unambiguously ascertained by X-ray analysis of the crystal structure of cycloadducts 4a, 5a and 5r, whose molecular structures are depicted in Fig. 4, 5 and 6, respectively. The crystal data collection and refinement data are summarized in Table 3.

**Table 3:** Crystal data collection and structure refinement of 4a, 5a and 5r

Compound/formula	4a/C_34_H_27_N_3_O_3_	5a/C_34_H_27_N_3_O_3_	5r/C_37_H_27_N_3_O_3_
Formula weight	525.58	525.58	561.61
Temperature/K	150(2)	150(2)	150(2)
Wavelength/Å	0.71073	0.71073	0.71069
Crystal system	Triclinic	Orthorhombic	Monoclinic
Space group	*P*1̄	*P*2_1_2_1_2_1_	*P*2_1_/*c*
*a*/Å	7.8139(9)	9.5047(6)	11.7916(5)
*b*/Å	12.9798(6)	11.4368(7)	9.7185(4)
*c*/Å	14.2280(7)	23.899(2)	24.5997(10)
*α*	65.084(5)°	90°	90°
*β*	80.892(11)°	90°	91.888(4)°
*γ*	79.404(3)°	90°	90°
Volume/Å^3^	1281.12(11)	2598.0(3)	2817.5(2)
*Z*	2	4	4
Density (calculated) g cm^−3^	1.362	1.344	1.324
Absorp. coefficient/mm^−1^	0.088	0.087	0.085
*F*(000)	552.0	1104.0	1176
Crystal size/mm^3^	0.10 × 0.10 × 0.41	0.19 × 0.11 × 0.07	0.30 × 0.20 × 0.10
Theta range for data collection/°	5.32 to 54.00	4.61 to 51.99	4.50 to 54.00
Index ranges	−9 ≤ *h* ≤ 9,	−10 ≤ *h* ≤ 11	−15 ≤ *h* ≤ 15
	−16 ≤ *k* ≤ 16	−12 ≤ *k* ≤ 14	−13 ≤ *k* ≤ 13
	−18 ≤ *l* ≤ 18	−26 ≤ *l* ≤ 29	−31 ≤ *l* ≤ 31
Reflections collected	33 514	11 626	39 113
Independent reflections	5562 [*R*(int) = 0.0384]	5098 [*R*(int) = 0.0419]	6140 [*R*(int) = 0.0470]
Refinement method	Full-matrix least-squares on *F*^2^	Full-matrix least-squares on *F*^2^	Full-matrix least-squares on *F*^2^
Data/restraints/parameters	5562/1/365	5098/1/365	6140/0/407
Goodness-of-fit on *F*^2^	1.041	1.032	1.016
Final *R* indices [*I* > 2sigma(*I*)]	*R*_1_ = 0.0423	*R*_1_ = 0.0451	*R*_1_ = 0.0440
	w*R*_2_ = 0.0973	w*R*_2_ = 0.0785	w*R*_2_ = 0.0934
*R* indices (all data)	*R*_1_ = 0.0563,	*R*_1_ = 0.0679,	*R*_1_ = 0.0607,
	w*R*_2_ = 0.1040	w*R*_2_ = 0.0875	w*R*_2_ = 0.1022
Largest diff. peak and hole/e Å^−3^	0.31 and −0.24	0.18 and −0.24	0.26 and −0.24

Elucidation of the three structures reveals that (i) the two carbonyl carbons of the enone part and isatin or acenaphthenequinone moieties are in *trans*-relationship, and (ii) a *cis*-relationship between the carbonyl of the oxindole or acenaphthenequinone ring and the proton attached at C-10b′. Thus, the cycloadducts are formed through an *exo*-approach between the *syn*-d_2_ and (*E*)-3-arylidene-1-phenyl-pyrrolidine-2,5-diones 1 (Scheme 4). The cycloaddition proceeds with high *exo*-diastereoselectivity affording in each case only one diastereomer. It is worth noting that our result is in contrast to the observed diastereoselectivity outcome from previously published studies.^11^ These works handled 1,3-dipolar cycloaddition reactions with tetrahydroisoquinolinium *N*-ylides derived from cyclic diketones. The authors revealed that the products were formed selectively through an *endo*-approach between *syn*-ylide and the dipolarophile.

### DFT calculations

To better grasp the experimentally observed high diastereoselectivity in the multicomponent 1,3-dipolar cycloaddition, calculations using the density functional approach (DFT) were performed. The effect of solvent (methanol) was estimated using the polarizable continuum model (PCM) approach.

**Scheme 5:**
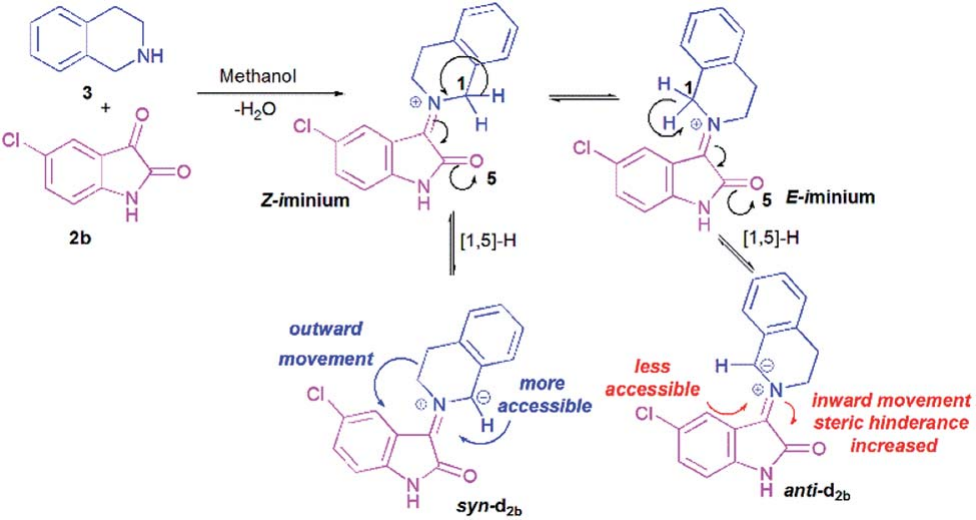
Generation of azomethine ylide d_2b_.

The model case study was the reaction of the dipolarophile 1a with azomethine ylide d_2b_ formed from the condensation of THIQ with chloroisatin 2b which should lead to the formation of the two types of iminium ions, *Z* and *E* (Scheme 5). For the generation of an azomethine ylide *via* a [1,5]-H shift, the oxygen of the carbonyl group of the *E* form is spatially too far from the benzylic hydrogen, while in the *Z* form they are close enough to permit a H-shift.^18^ So, one can expect that the *syn*-d_2b_*N*-ylide would be formed from the *Z*-iminium salt.

Moreover, and in accordance with our previous work,^16*b*,17*b*^ the attack of the dipolarophile on *syn*-d_2b_ would enforce the inward movement of THIQ ring to the cyclic diketones core, thus enhancing the steric hindrance between these later nuclei. In the case of *anti*-d_2b_, the 1,3-dipolar cycloaddition of the *N*-ylide would be favoured because no apparent steric hindrance would have evolved from the outward movement of the THIQ core.^14,15*b*^ Therefore, only the *syn-N*-ylide forming azomethine ylide d_2b_ are considered in the subsequent calculations.

**Scheme 6:**
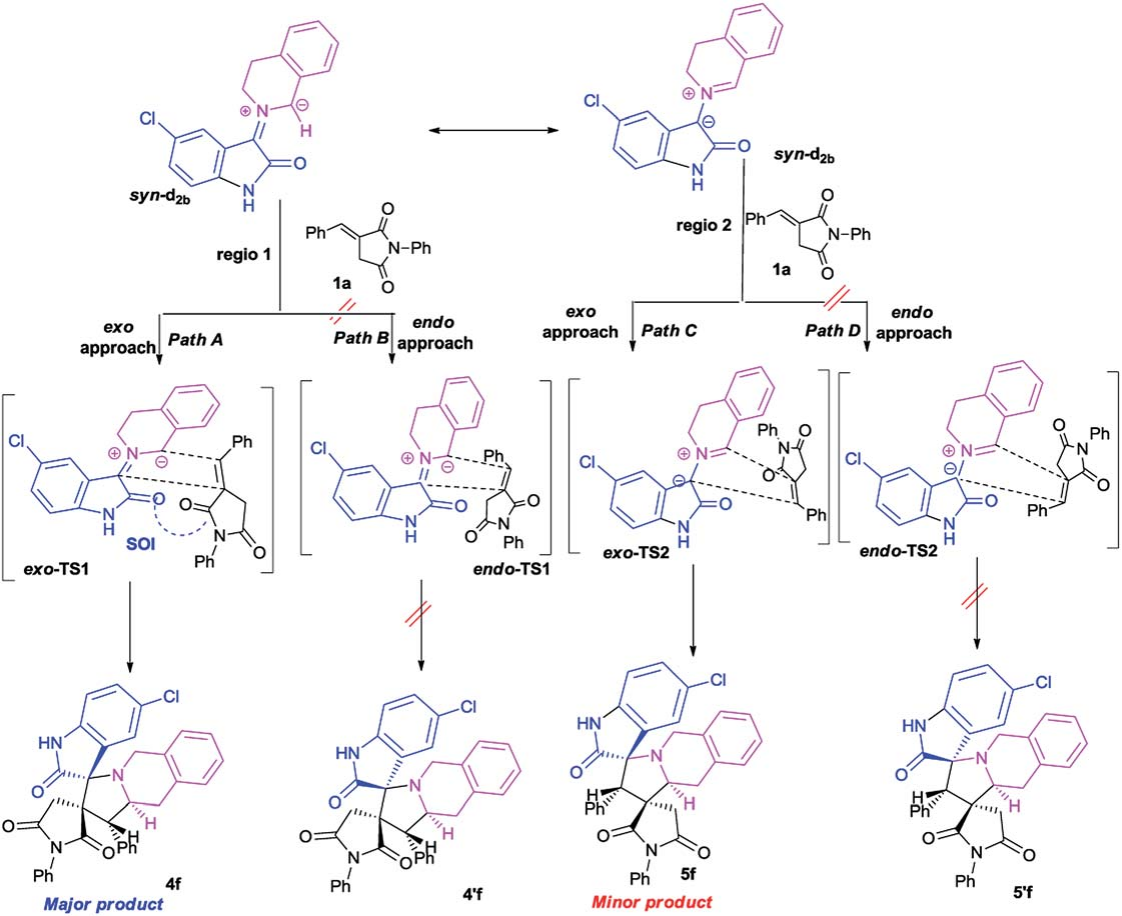
Plausible mechanism for the regio- and stereoisomeric 1,3-dipolar cycloaddition reaction of dipolarophile 1a with *syn*-d_2b_.

**Fig. 7:**
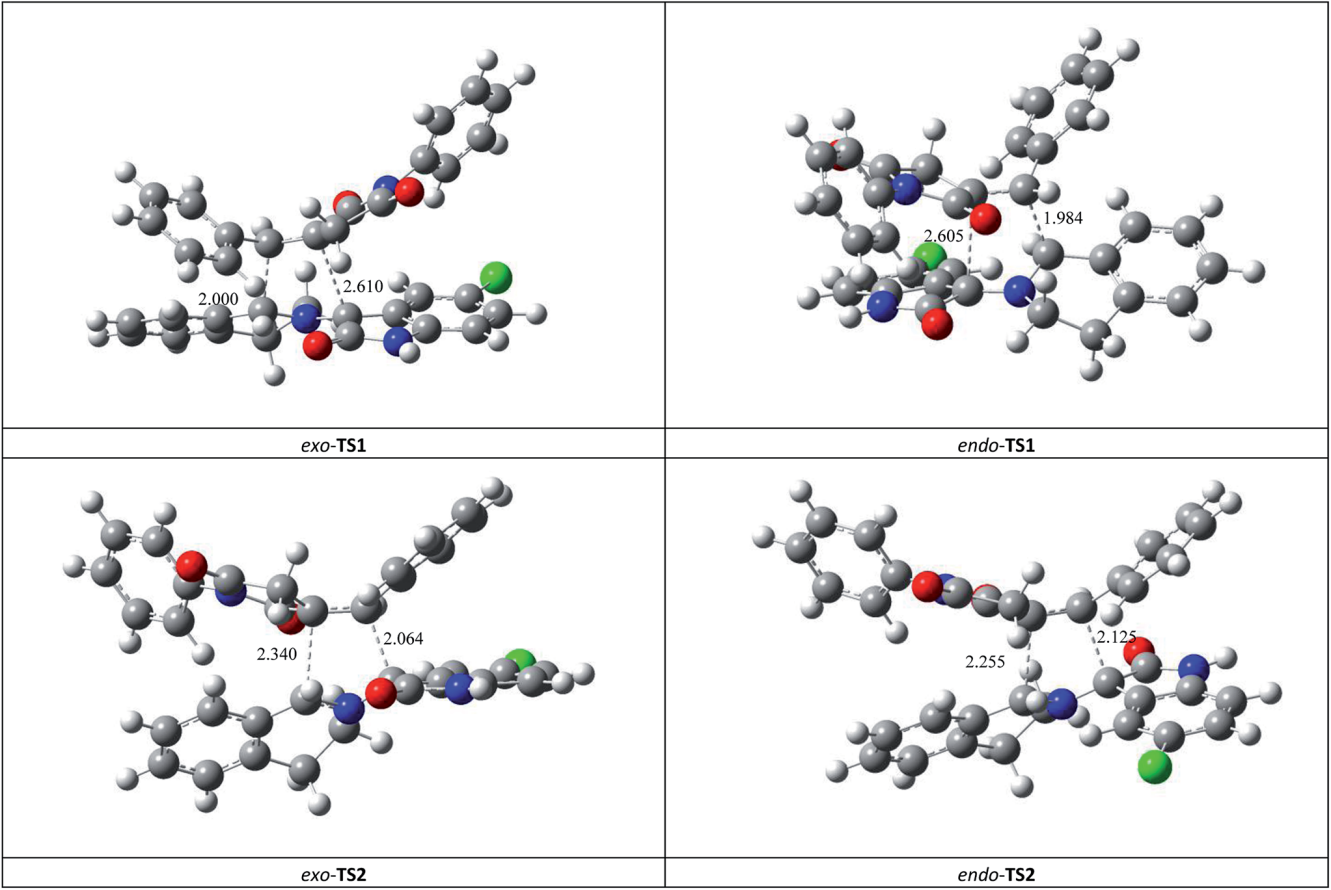
Four possible TS for the 1,3-dipolar cycloaddition of *syn*-d_2b_ across 1a, optimized at the B3LYP/6-31G(d,p) level. The lengths of the bonds directly involved in the reaction are given in Å. Coordinates are given in the ESI.†

This *syn* intermediate could in principle react with the dipolarophile 1a along four reactive channels related to two regioisomeric and two stereoisomeric approaches (Scheme 6). The cycloadducts were labelled 4f, 4′f, 5f, 5′f and their transition states (TS) *exo*-TS1, *endo*-TS1, *exo*-TS2 and *endo*-TS2 were energetically optimized and characterized under the same level of calculation by using the Berny analytical gradient method.^19^ These TS, which are characterized by a saddle point with a single imaginary frequency, are depicted in Fig. 7. The differences between reactants and transition states in the Gibbs free energy (Δ*G*), enthalpy (Δ*H*), entropy (Δ*S*) and internal energy (Δ*U*), for the different stationary points along the reactive pathways are compiled in Table 4.

**Table 4:** Relative Gibbs free energies at 298.15 K (Δ*G*, in kcal mol^−1^), enthalpies (Δ*H*, in kcal mol^−1^) and entropies (Δ*S*, in cal mol^−1^ K^−1^) for TSs and adducts of 1,3-dipolar cycloaddition between *syn*-d_2b_ and 1a, calculated at the B3LYP/6-31G(d,p) level

	Δ*G*	Δ*H*	Δ*S*	Δ*U*
4f	3.1	−10.6	−46.0	−13.7
4′f	6.6	−6.7	−44.8	−9.4
5f	6.7	−8.7	−51.6	−11.1
5′f	9.6	−5.4	−50.1	−7.7
*exo-*TS1	23.8	9.5	−47.8	8.3
*endo*-TS1	25.5	11.7	−46.1	10.7
*exo-*TS2	23.1	9.3	−46.2	8.2
*endo*-TS2	26.7	13.2	−45.3	12.1

All the energy values have been corrected for the zero point energy. The kinetic parameters (activation energy Δ*G*^#^, activation enthalpies Δ*H*^#^, and activation entropies Δ*S*^#^ between reactants and TS) are first discussed.

The corresponding activation parameters, Δ*H*^#^, Δ*S*^#^ and Δ*G*^#^, are in fairly good agreement with the experiment results. However, the strong negative entropy values of *exo*-TS1 and *exo*-TS2 suggest a highly ordered rate-determining transition state, as expected for a polar concerted cycloaddition.^20^

The potential energy requested to reach the TS, *i.e.*, the activation energy, was shown to increase in the following order: *exo*-TS2 < *exo*-TS1 < *endo*-TS1 < *endo*-TS2.

The favoured *exo* approaches compared with the *endo* attack agree well with experimental findings showing that mixtures of the two *exo*-regioisomers are observed only.

The thermodynamic parameters, Δ*G*, Δ*H* and Δ*S*, between reactants and products of the investigated reactions are reported in Table 4.

The lowest Δ*G* value is achieved for the *exo* approach on 4f (3.1 kcal mol^−1^). In addition, cycloadditions leading to 4f, 5f, 4′f and 5′f are exothermic processes, and the most negative values of Δ*H* correspond to 4f and 5f (−10.6 and −8.7 kcal mol^−1^, respectively). It is worth noting that calculations in methanol solution indicate positive free Gibbs energies (Δ*G* > 0) for these reactions. Moreover, the cycloadduct 4f resulting from the *exo* approach of *syn*-d_2b_ with dipolarophile 1a is the most favourable thermodynamic product. TSs *exo*-TS1 and *exo*-TS2 of the two cycloadducts 4f and 5f respectively, exhibit comparable energies. They are thus the two outcomes, but due to a clear difference in energy, 4f is the major product.

All the TS are concerted asynchronous as expected for a typical normal-demand 1,3-dipolar cycloaddition. To support this accordance, the two new *σ* bonds lengths *I*_1_ and *I*_2_ are measured. At the TSs associated with the approach affording the cycloadducts 4f and 4′f (Fig. 7), the length of the C-1′–C-10b′ forming bonds are 1.991 Å at *exo*-TS1 and 1.974 Å at *endo*-TS1, while the distance between the C-2′ and the C-3′ atoms is 2.469 Å at *exo*-TS1 and 2.649 Å at *endo*-TS1. However, the opposite trend is found for fused transition structures (*exo*-TS2 and *endo*-TS2), with forming C-1′–C-3′ bond lengths of 1.996 Å and 2.001 Å, respectively. The computed distance between the C-2′ and the C-10b′ atoms is 2.490 Å at *exo*-TS2 and 2.417 Å at *endo*-TS2.

The extent of the asynchronicity of the bond-formation can thus be measured by the difference between the lengths of the two *σ* bonds being formed in the reaction, *i.e.*, Δ*d* = *l*_1_ − *l*_2_. The asynchronicity at the first trend leading 4f and 4′f is Δ*d* = 0.478 Å at *exo*-TS1 and 0.675 Å at *endo*-TS1, while that at the second tend affording 5f and 5′f is Δ*d* = 0.494 Å at *exo*-TS2 and 0.416 Å at *endo*-TS2. Therefore, the formation of the TSs associated with 4f and 5f is favoured. In addition, these geometrical parameters indicate that these cycloadditions correspond to asynchronous concerted processes.

Frontier molecular orbitals (FMO) have been determined in order to get global reactivity indices. These indices, namely, electronic chemical potential, *μ*, chemical hardness, *η*, and global electrophilicity, *ω*, are powerful tools for studying the feasibility of the reaction, as well as the reactivity of the reagents.^21^ They have been computed for the separated reagents involved in the [3+2] cycloaddition reaction of dipolarophile 1a with azomethineylide *syn*-d_2b_. The chemical hardness *η* reveals the stability and reactivity of a chemical system.^22–24^ The global electrophilicity *ω* measures the propensity or capacity of a species to accept electrons.^24*g*,25^ All the data are displayed in Table S1.†

Analysis of the reactive indices suggests that the electronic chemical potential *μ* of dipole *syn*-d_2b_ (−3.921 eV) is higher than that of dipolarophile 1a (−4.343 eV) and the chemical hardness of *syn*-d_2b_ (*η* = 3.624 eV) is lower than of 1a (*η* = 4.595 eV). This result indicates that the charge transfer will take place from the dipole toward dipolarophile. Over and above, the electrophilicity of dipolarophile 1a (*ω* = 2.052 eV) is greater than that of *syn*-d_2b_ (*ω* = 2.237 eV), indicating that the 1,3-dipole will act as a nucleophile due to its low *ω* value and high global nucleophilicity values (*N* = 4.422 eV). However, the dipole *syn*-d_2b_ is classified as strong nucleophiles due to its high nucleophilicity index, *N* > 3 eV.^26^1a will thus act as an electrophile.

After evaluation of the global nucleophilic/electrophilic character, a local analysis was carried out with the calculation of condensed Fukui functions considering the cationic and anionic systems in their optimized geometries of the corresponding neutral systems. Fukui function parameters have been evaluated through the calculation of the electrostatic potential (ESP) derived atomic populations.^27^ The calculated local chemical reactivity parameters of *syn*-d_2b_ and dipolarophile 1a are shown in Fig. S1 (in the ESI).† We can notice that C-10b′ of azomethine ylide *syn*-d_2_ has more nucleophilic character (*f*^−^ = 0.162). On the other hand, the dipolarophile 1a has the highest electrophilic centre at C-1′ (*f*^+^ = 0.118). The electrophilicity at C-1′ can be attributed to the presence of a strong electron-withdrawing carbonyl group. Thereby, the most favourable nucleophilic/electrophilic interaction will occur between C-10b′ carbon atom of *syn*-d_2_ and C-1′ atom of enone 1a leading to preferred formation of major regioisomer 4f. These results perfectly agree with the experimental observations (Scheme 5).

## Conclusion

We have thus developed an efficient method for the synthesis of a novel class of dispiropyrrolo[2,1-*a*]isoquinolines fused pyrrolidine-2,5-dione bearing two adjacent spiro-carbons in good to high yields with high diastereoselectivity. Our strategy to prepare these cycloadducts involves three-component 1,3-dipolar cycloaddition reaction between cyclic diketones as tetrahydroisoquinolinium *N*-ylides precursors and α-alkylidenesuccinimides as dipolarophiles. More importantly, these findings proved the versatile utility of this strategy for the synthesis of complex dispiroheteropolycyclic systems, which is of great importance for the development of medicinally active drugs.

An analysis based on theoretical calculations using the DFT approach, B3LYP/6-31G(d,p) reveals that the isomeric spirocycloadducts 4 and 5 are obtained through a 1,3-dipolar cycloaddition reaction *via* a high asynchronous mechanism with very low activation energies, when compared to the other possible reaction paths. This outcome is in agreement with the experimental observations.

## Experimental

### Apparatus and general information

NMR spectra were recorded with a Bruker-Spectrospin AC 300 spectrometer operating at 300 MHz for ^1^H and 75 MHz for ^13^C using tetramethylsilane as the internal standard (0.00 ppm) in CDCl_3_ as solvent. The following abbreviations were used to explain the multiplicities: bs = broad singlet, s = singlet, d = doublet, m = multiplet.

Elemental analyses were performed on a Perkin Elmer 2400 Series II Elemental CHNS analyzer. Materials: thin-layer chromatography (TLC): TLC plates (Merck, silica gel 60 F_254_ 0.2 mm 200 × 200 nm); substances were detected using UV light at 254 nm.

### Typical representative procedure for preparation of cycloadducts 4a–5a

A mixture of 1a (2 63 mg, 1.0 mmol), isatin 2a (147 mg, 1 mmol) and tetrahydroisoquinoline 3 (159.7 mg, 1.2 mmol) was refluxed in methanol (5 mL) for 4 h. After completion of the reaction (TLC), the solvent was removed under vacuum. The residue was chromatographed on silica gel employing ethyl acetate-cyclohexane (3 : 7 v/v) as eluent to obtain the pure products 4a (0.26 g, 51%) and 5a (0.17 g, 34%). Spectroscopic data and further experimental details for the all compound are presented in the ESI.†

### DFT calculations

The geometric optimizations of all reactants, TS, and products were performed using the B3LYP functional and the 6-31G(d,p) basis set in the Gaussian 09 (ref. 27) environment, by using the Berny analytical gradient method.^28^ The global electrophilicity index, *ω*, was calculated following the expression,^24,25^*ω* = (*μ*^2^/2*η*), where *μ* is the electronic chemical potential, *μ* = (*E*_HOMO_ + *E*_LUMO_)/2, and *η* is the chemical hardness, *η* = (*E*_LUMO_ − *E*_HOMO_). The Fukui condensed functions have been calculated from local populations found from single point calculations carried out on reduced, neutral, and oxidized forms with geometries optimized for neutral species. The most favourable attack site can also be disclosed by theoretical calculations. The actual DFT-based local chemical reactivity Fukui function parameters for nucleophilic (*f*_k_^+^) and electrophilic (*f*_k_^−^) attacks were calculated through the electrostatic potential (ESP) derived atomic populations.^26^

Characterizations have been performed under the same level of calculation. Vibrational analysis has been performed for all the stationary points. TS are characterized by a single negative imaginary frequency. For all TS structures, the intrinsic reaction coordinate^27^ calculation, using the Hessian-based predictor–corrector method,^28^ was performed to ascertain that each TS connected the expected reactants and products. Thermal corrections were computed from unscaled frequencies for a standard state of 298.15 K and 1 atm in methanol solution and within the harmonic approximation.

### Crystal structure determinations

A suitable crystal of 4a, 5a and 5r was selected and mounted on an Xcalibur, Sapphire3 diffractometer. The crystals were kept at 150(2) K during data collection. Using Olex2,^29^ the structures were solved with the ShelXS^30^ structure solution program using Direct Methods and refined with the XL^30^ refinement package using Least Squares minimisation.

## Conflicts of interest

There are no conflicts to declare.

## Supplementary Material

RA-009-C8RA09884K-s001

RA-009-C8RA09884K-s002
