# Supplementary material for: Highly diastereoselective construction of novel dispiropyrrolo[2,1-a]isoquinoline derivatives via multicomponent 1,3-dipolar cycloaddition of cyclic diketones-based tetrahydroisoquinolinium N-ylides
Source: RSC Adv. 2019 Apr 9;9(20):11082–91. doi: 10.1039/c8ra09884k (PMC9092959; doi:10.1039/c8ra09884k)
Supplement: RA-009-C8RA09884K-s001 [file RA-009-C8RA09884K-s001.pdf]

Electronic Supplementary Material (ESI) for RSC Advances.
This journal is © The Royal Society of Chemistry 2019

Highly diastereoselective construction of novel dispiropyrrolo[2,1-a]isoquinoline derivatives *via* multicomponent 1,3-dipolar cycloaddition of cyclic diketones-based tetrahydroisoquinolinium *N*-ylides

Sarra Boudriga,[a,*] Saoussen Haddad,[a] Mohеddine Askri,[a,*] Armand Soldera,[b] Michael Knorr, [c]Carsten Strohmann,[d] Christopher Golz[d]

[a] *Laboratory of Heterocyclic Chemistry Natural Products and Reactivity/CHPNR, Department of Chemistry, Faculty of Science of Monastir, 5000 Monastir, Tunisia.E-mail: moheddine.askri@fsm.rnu.tn;Tel: +216 98676187*
[b] *Institute UTINAM - UMR CNRS 6213, University of Franche-Comté, 16 Route de Gray, F-25030 Besançon, France.*
[c] *Technische Universität Dortmund, Anorganische Chemie Otto-Hahn-Strasse 6, 44221 Dortmund (Germany)*

Supporting information

Table of contents **Page**

1 DFT data page 2
2 Cartesian coordinates and energies for **1a**, ***syn*-d$_{2b}$**, ***exo*-TS1**, ***endo*-TS1**, ***exo*-TS2**, ***endo*-TS2**, **4f, 4'f**, **5f** and **5'f** at the B3LYP/6-31G(d,p) level page 2-17
3 Experimental Section page 18
3.1. General procedure for the preparation of cycloadducts **4–5**
3.2 Spectra data of synthesized compounds **4a-r** and **5a-r** page 18-23
3.3 ^{1}H- and ^{13}C-NMR Spectra of compounds 4**a-r and** 5**a-r** (Fig. S1 to S58) page 24-52

1. DFT data

Table S1. HOMO/LUMO energies, global electrophilicity, electronic chemical potential, chemical hardness of the species, electronegativity and index of reactants (in eV) calculated at the B3LYP/6-31G(d,p) level

	**HOMO**	**LUMO**	**μ**	**X**	**η**	**ω**	**N**
**syn-d$_{2a}$**	-0,211	-0,077	-3,921	3,921	3,624	2,121	4.423
**1a**	-0,244	-0,075	-4,343	4,343	4,595	2,052	2.479

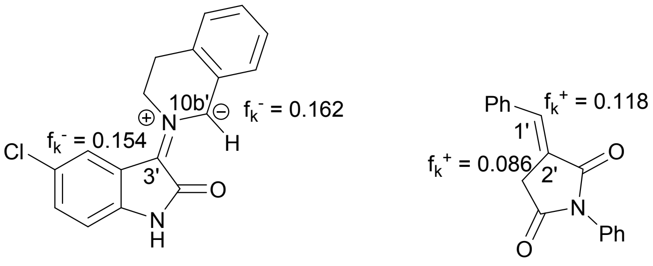

***syn*-d$_{2b}$** **1a**

Figure S1. Fukui Function in the reactive centers of the species involved in the 1,3-dipolar cycloaddition (according to the ESP scheme).

2. Cartesian coordinates and energies for 1a, *syn*-d$_{2a}$, *exo*-TS1, *endo*-TS1, *exo*-TS2, *endo*-TS2, 4f, 4'f, 5f and 5'f at the B3LYP/6-31G(d,p) level

- **1a**

Coordinates (Angstroms)

Atom	X	Y	Z
C	0.538104	-0.755159	0.079733
C	-0.780484	-0.073669	-0.006336
C	-0.532246	1.393616	-0.198809
C	0.987864	1.526944	-0.204767
N	1.537003	0.244227	-0.042194
H	-0.936790	2.022537	0.602992
C	2.941469	-0.024702	-0.002893
C	3.453059	-1.150289	-0.658379
C	3.797717	0.839006	0.689296
C	4.821912	-1.408562	-0.613059
H	2.783891	-1.821120	-1.180947
C	5.165616	0.573372	0.717901
H	3.397908	1.714396	1.184141
C	5.682780	-0.549463	0.070876
H	5.214159	-2.285744	-1.118728
H	5.827050	1.248602	1.252285
H	6.748865	-0.753592	0.099595
C	-1.907237	-0.806421	0.098382
H	-1.731300	-1.872145	0.239167

H	-0.920183	1.790596	-1.144433
C	-3.312103	-0.410860	0.058245
C	-4.279926	-1.423401	0.218078
C	-3.765080	0.911473	-0.128568
C	-5.640330	-1.132746	0.195407
H	-3.948616	-2.448077	0.362348
C	-5.126334	1.200251	-0.151488
H	-3.056469	1.719585	-0.258078
C	-6.070118	0.182731	0.010401
H	-6.365348	-1.931131	0.321679
H	-5.453835	2.225392	-0.296934
O	0.756030	-1.942425	0.226430
O	1.614563	2.556715	-0.326889
H	-7.131062	0.414078	-0.008184

Zero-point correction = 0.259160Hartree
ZPE is included in the following quantities:
Electronic energy = -860.623580 Hartree
Internal energy = -860.607784 Hartree
Enthalpies= -860.606840 Hartree
Gibbs energy = -860.668766 Hartree
Number of imaginary frequencies: 0

- ***syn*-d$_{2b}$**

Coordinates (Angstroms)

Atom	X	Y	Z
C	2.652873	1.527773	0.150353
C	1.783245	0.400915	0.006333
C	2.373486	-0.862242	-0.173795
C	3.764693	-0.967552	-0.156444
C	4.601388	0.134321	0.013902
C	4.033876	1.405035	0.160162
C	0.517069	2.385495	0.135983
C	0.442696	0.933017	0.029899
H	1.791100	-1.755373	-0.354721
H	5.677059	0.005878	0.021191
H	4.668402	2.278647	0.274801
N	1.879577	2.672176	0.245160
H	2.220197	3.618645	0.305153
O	-0.380698	3.236022	0.154158
C	-0.783175	-1.198207	0.242722
C	-1.937989	0.878994	-0.220830
C	-2.025468	-1.647159	1.006520
H	-0.725868	-1.683572	-0.738162
C	-3.197593	0.184181	-0.183108
C	-3.286582	-1.107344	0.383267

H	-2.031239	-2.741195	1.042096
C	-4.358837	0.792819	-0.699212
H	-4.291777	1.788863	-1.127274
C	-4.508680	-1.770545	0.404748
H	-4.572075	-2.763175	0.843492
C	-5.574815	0.118549	-0.671275
H	-6.463709	0.592137	-1.076631
C	-5.653417	-1.165902	-0.124758
H	-6.602055	-1.693348	-0.105889
H	-1.880962	1.938323	-0.437091
N	-0.772719	0.275070	0.010667
H	0.111762	-1.443423	0.808548
H	-1.951491	-1.297500	2.046151
Cl	4.487742	-2.565688	-0.373678

Zero-point correction = 0.264247 Hartree
ZPE is included in the following quantities:
Electronic energy = -1300.322345 Hartree
Internal energy = -1300.306125 Hartree
Enthalpies= -1300.305181 Hartree
Gibbs energy = -1300.366719 Hartree
Number of imaginary frequencies: 0

- ***exo*-TS1**

Coordinates (Angstroms)

Atom	X	Y	Z
C	1.964025	-1.869119	1.775371
C	1.376849	-1.819240	0.482678
C	2.200183	-2.056146	-0.627642
C	3.539190	-2.388221	-0.412741
C	4.095579	-2.458114	0.863786
C	3.299424	-2.182610	1.978851
C	-0.230050	-1.280263	2.139320
C	-0.017149	-1.422052	0.670241
H	1.844997	-1.952662	-1.642074
H	5.141545	-2.713434	0.985956
H	3.721903	-2.212273	2.977906
N	0.998445	-1.548375	2.720159
H	1.140561	-1.477205	3.716141
O	-1.237951	-0.981517	2.782053
C	-0.835672	-2.027641	-1.580390
C	-2.234011	-0.865898	0.061261
C	-2.049999	-2.768861	-2.124197
H	-0.581850	-1.152554	-2.189131
C	-3.417634	-1.146530	-0.790479
C	-3.335591	-2.018447	-1.891830

H	-1.890982	-2.955823	-3.191583
C	-4.658061	-0.563431	-0.477028
H	-4.743180	0.077283	0.392358
C	-4.465099	-2.250657	-2.679976
H	-4.389307	-2.927195	-3.527689
C	-5.777478	-0.802643	-1.269017
H	-6.725117	-0.338767	-1.013034
C	-5.681481	-1.638906	-2.382697
H	-6.550811	-1.824641	-3.006373
N	-1.067153	-1.570556	-0.191421
H	0.016099	-2.701722	-1.568251
H	-2.117873	-3.754060	-1.641532
C	-0.326423	1.157917	0.412933
C	-1.631691	1.030931	-0.139362
H	-2.438849	-0.777051	1.121420
Cl	4.562775	-2.708973	-1.810896
H	-1.615131	1.027058	-1.228820
C	0.834196	1.193140	-0.482774
C	1.525879	2.186272	1.519914
C	0.055898	1.838236	1.696192
H	-0.083381	1.243974	2.605143
H	-0.503206	2.769489	1.859271
O	2.250098	2.689491	2.353852
N	1.899253	1.839106	0.210218
O	0.913161	0.780911	-1.634157
C	3.163309	2.169912	-0.371259
C	3.785202	1.294392	-1.268619
C	3.773405	3.390476	-0.050880
C	5.008306	1.642950	-1.839715
H	3.309737	0.359640	-1.525391
C	5.000760	3.721251	-0.621073
H	3.298769	4.064039	0.650120
C	5.623458	2.852704	-1.518509
H	5.480762	0.955979	-2.535331
H	5.467995	4.666890	-0.362407
H	6.578976	3.116392	-1.962106
C	-2.750240	1.844911	0.425034
C	-3.488234	2.674086	-0.434614
C	-3.095757	1.818949	1.787540
C	-4.529970	3.464357	0.049821
H	-3.238404	2.700402	-1.491661
C	-4.141163	2.607273	2.269994
H	-2.563863	1.157445	2.464932
C	-4.861044	3.434330	1.405350

H	-5.084097	4.101414	-0.633520
H	-4.396036	2.569993	3.325267
H	-5.674182	4.046383	1.784180

Zero-point correction = 0.525230 Hartree
ZPE is included in the following quantities:
Electronic energy = -2160.923276 Hartree
Internal energy = -2160.890804 Hartree
Enthalpies = -2160.889860 Hartree
Gibbs energy = -2160.987628 Hartree
Number of imaginary frequencies: 1 (-362.6156)

- ***endo*-TS1**

Coordinates (Angstroms)

Atom	X	Y	Z
C	0.947504	0.761444	2.715716
C	1.896226	0.520881	0.484408
C	2.282764	1.239553	3.267896
H	0.303674	1.598994	2.429121
C	2.967002	1.480078	0.862103
C	3.144530	1.872266	2.202860
H	2.087598	1.947690	4.080289
C	3.843666	1.974433	-0.118691
H	3.735102	1.650342	-1.147392
C	4.162270	2.772963	2.526224
H	4.294464	3.070698	3.563419
C	4.852198	2.873136	0.217412
H	5.517767	3.248031	-0.554040
C	5.008414	3.282648	1.542800
H	5.791376	3.985808	1.810644
N	1.176400	-0.078694	1.507107
H	0.398985	0.169291	3.441404
H	2.822799	0.392625	3.714802
C	-0.691406	0.571734	-0.576082
C	0.482750	1.381228	-0.610368
C	0.308951	-1.076920	1.176123
C	0.551875	-2.205587	0.281743
C	-0.925025	-1.405254	1.953601
C	-0.563089	-3.076934	0.410409
H	2.190814	-0.162808	-0.298132
O	-1.455043	-0.825412	2.894355
N	-1.430601	-2.556772	1.359561
H	-2.281160	-2.988406	1.687584
C	1.596576	-2.587812	-0.570659
C	-0.669525	-4.261938	-0.304967
H	-1.536932	-4.904504	-0.195277
C	1.489576	-3.789896	-1.273677
C	0.373205	-4.619141	-1.163978

H	0.320978	-5.536364	-1.738480
H	2.500301	-2.003411	-0.681008
Cl	2.811435	-4.266967	-2.336247
H	0.368444	2.268509	0.011502
C	-1.261759	-0.291859	-1.664780
H	-1.188488	0.173132	-2.656797
H	-0.816661	-1.288163	-1.758646
C	-1.804213	0.994711	0.290209
C	-2.734983	-0.445690	-1.301770
O	-3.554199	-1.121887	-1.889228
O	-1.780373	1.790867	1.215454
N	-2.982341	0.340771	-0.169603
C	-4.269032	0.503906	0.430262
C	-4.396266	0.480101	1.823941
C	-5.394518	0.687536	-0.381036
C	-5.655620	0.652301	2.397156
H	-3.520288	0.327569	2.442241
C	-6.648312	0.845432	0.206696
H	-5.287698	0.691145	-1.458443
C	-6.783709	0.833411	1.595655
H	-5.752326	0.638428	3.478830
H	-7.520084	0.982681	-0.426209
H	-7.761924	0.961936	2.049670
C	1.144315	1.673960	-1.918415
C	1.546196	0.668749	-2.814751
C	1.379970	3.011651	-2.275992
6	2.157421	0.992037	-4.026849
H	1.393134	-0.376801	-2.562868
C	1.985917	3.335704	-3.489027
H	1.080400	3.802887	-1.594728
C	2.379218	2.326769	-4.369860
H	2.461402	0.197449	-4.702014
H	2.152747	4.378169	-3.744278
H	2.854479	2.577317	-5.313443

Zero-point correction = 0.524750 Hartree
ZPE is included in the following quantities:
Electronic energy = -2160.918647 Hartree
Internal energy = -2160.886066 Hartree
Enthalpies = -2160.885122 Hartree
Gibbs energy = -2160.983177 Hartree
Number of imaginary frequencies: 1 (-375.9301)

- ***exo*-TS2**

Coordinates (Angstroms)

Atom	X	Y	Z
C	4.146929	0.218102	-0.611981
C	3.048360	0.433338	0.250372

C	3.231635	0.294402	1.627006
C	4.513665	0.005157	2.105491
C	5.597633	-0.178467	1.248513
C	5.415186	-0.081987	-0.134703
C	2.365336	0.609469	-2.016581
C	1.846624	0.609538	-0.595548
H	2.410692	0.392765	2.326089
H	6.575971	-0.404059	1.656524
H	6.248682	-0.240887	-0.811217
N	3.719730	0.351028	-1.931253
H	4.281741	0.192904	-2.754012
O	1.752376	0.786130	-3.062440
C	0.712149	2.145613	1.028848
C	-0.282012	1.457301	-1.092375
C	-0.104221	3.432698	0.963096
H	0.245411	1.419615	1.701641
C	-1.442428	2.298914	-0.818298
C	-1.408150	3.241807	0.229260
H	-0.277283	3.788490	1.983772
C	-2.600675	2.181048	-1.606884
H	-2.624218	1.470116	-2.426980
C	-2.535924	4.021819	0.485775
H	-2.506617	4.752243	1.290325
C	-3.721118	2.958946	-1.335436
H	-4.614803	2.847689	-1.940806
C	-3.692045	3.877610	-0.283031
H	-4.565445	4.485016	-0.066134
N	0.826696	1.531673	-0.317013
H	1.720419	2.367240	1.375676
H	0.483375	4.209684	0.453439
C	0.790583	-1.158502	-0.446865
C	-0.569507	-0.841021	-0.752746
H	0.954198	-1.197087	0.628463
C	-1.379157	-1.323500	-1.931527
C	-1.492783	-0.668829	0.375030
C	-2.824936	-1.265362	-1.454087
C	1.564721	-2.206183	-1.164518
C	2.371624	-3.074338	-0.406477
C	1.532962	-2.388155	-2.560574
C	3.101188	-4.096486	-1.010618
H	2.414881	-2.948519	0.671662
C	2.264508	-3.410660	-3.163773
H	0.965804	-1.706300	-3.182264
C	3.047060	-4.272938	-2.393900
H	3.708785	-4.756872	-0.398573
H	2.222628	-3.531150	-4.242624

O	-1.232915	-0.376655	1.535979
N	-2.813773	-0.911872	-0.098572
O	-3.814485	-1.487322	-2.122279
C	-3.985148	-0.811131	0.711324
C	-4.994798	-1.773473	0.595467
C	-4.122964	0.246624	1.617110
C	-6.138836	-1.671780	1.384914
H	-4.888982	-2.581817	-0.116703
C	-5.266597	0.329401	2.409624
H	-3.337341	0.985473	1.702343
C	-6.278433	-0.624994	2.297011
H	-6.920972	-2.418837	1.287366
H	-5.366520	1.149067	3.115175
H	-7.169110	-0.552972	2.914174
H	-1.284302	-0.734198	-2.850474
H	-1.151304	-2.360827	-2.207901
H	-0.112099	1.129405	-2.107759
H	3.610435	-5.071621	-2.867436
Cl	4.758260	-0.140668	3.843476

Zero-point correction = 0.525026 Hartree
ZPE is included in the following quantities:
Electronic energy = -2160.921882 Hartree
Internal energy = -2160.889344 Hartree
Enthalpies = -2160.888400 Hartree
Gibbs energy = -2160.986698 Hartree
Number of imaginary frequencies: 1 (-362.2742)

- ***endo*-TS2**

Coordinates (Angstroms)

Atom	X	Y	Z
C	0.387800	2.123574	2.004941
C	0.218874	1.314146	-0.286314
C	-0.061610	3.502349	1.535311
H	-0.446691	1.554966	2.422576
C	-0.754257	2.366490	-0.587175
C	-0.948832	3.430170	0.317027
H	-0.578571	3.999133	2.362647
C	-1.485698	2.332334	-1.787271
H	-1.326656	1.525035	-2.496198
C	-1.887843	4.417867	0.015303
H	-2.035337	5.240270	0.710628
C	-2.423690	3.319229	-2.071105
H	-2.992358	3.273882	-2.994169
C	-2.629859	4.362629	-1.165102
H	-3.362499	5.134349	-1.380863
N	0.955870	1.360815	0.856036
H	1.157121	2.204859	2.767773

H	0.819467	4.117008	1.300368
C	0.359861	-1.253658	0.954237
C	-0.615271	-0.758342	0.027458
H	0.054820	-1.074414	1.983768
C	-0.897204	-1.318294	-1.348365
C	-1.911586	-0.319016	0.585968
C	-2.361242	-0.990058	-1.610862
C	1.083213	-2.536501	0.792637
C	1.233553	-3.356327	1.928257
C	1.628370	-2.992659	-0.422300
C	1.874625	-4.590197	1.848818
H	0.829961	-3.017332	2.878357
C	2.272440	-4.227221	-0.500383
H	1.574414	-2.372926	-1.309301
C	2.393771	-5.034538	0.631041
H	1.965022	-5.207052	2.738418
H	2.684438	-4.554997	-1.450356
O	-2.155533	0.097268	1.707181
N	-2.895824	-0.457890	-0.432299
O	-2.962362	-1.162535	-2.651623
C	-4.267726	-0.092781	-0.270779
C	-5.271012	-0.927580	-0.775346
C	-4.604562	1.094242	0.388935
C	-6.608861	-0.569680	-0.619155
H	-5.002939	-1.837853	-1.296480
C	-5.946902	1.434032	0.548992
H	-3.823528	1.732706	0.779985
C	-6.952712	0.608030	0.045781
H	-7.383537	-1.218674	-1.016533
H	-6.204613	2.352858	1.067440
H	-7.996652	0.880082	0.170227
H	-0.290358	-0.914572	-2.167223
H	-0.780200	-2.408467	-1.390764
C	1.819939	0.286557	1.071030
C	2.939365	-0.034859	0.153578
C	2.325479	-0.045097	2.450409
C	3.937417	-0.674263	0.925579
H	0.691425	0.846314	-1.135719
O	1.782133	0.106381	3.535948
N	3.534686	-0.692066	2.256775
H	4.039503	-1.108911	3.023895
C	3.182414	0.177701	-1.203695
C	5.125117	-1.134937	0.374963
H	5.873920	-1.626800	0.987449
C	4.385940	-0.275229	-1.753536
C	5.346285	-0.936411	-0.990868

H		6.261676	-1.284059	-1.455178
H		2.483454	0.696359	-1.846876
H		2.890761	-5.997916	0.565577
Cl		4.686180	-0.004285	-3.468908

Zero-point correction = 0.524702 Hartree
ZPE is included in the following quantities:
Electronic energy = -2160.914445 Hartree
Internal energy = -2160.881817 Hartree
Enthalpies = -2160.880872 Hartree
Gibbs energy = -2160.979138 Hartree
Number of imaginary frequencies: 1 (-378.2990)

- **4f**

Coordinates (Angstroms)

Atom		X	Y	Z
6	0	1.532876	-2.622593	2.067269
6	0	1.288258	-1.992035	0.834150
6	0	2.236047	-2.107171	-0.179139
6	0	3.400969	-2.842859	0.069757
6	0	3.633291	-3.463415	1.294429
6	0	2.686269	-3.354721	2.316589
6	0	-0.487773	-1.564549	2.386323
6	0	-0.065547	-1.283806	0.892714
1	0	2.087364	-1.639357	-1.142771
H		4.546256	-4.026066	1.452145
H		2.855312	-3.831283	3.276773
N		0.473465	-2.374237	2.941754
H		0.394005	-2.721167	3.886602
O		-1.478409	-1.167326	2.973735
C		-0.787118	-2.388288	-1.253467
C		-2.234357	-0.900006	0.105800
C		-1.998486	-3.105643	-1.835716
H		-0.455727	-1.588786	-1.931852
C		-3.314474	-1.156037	-0.934278
C		-3.206968	-2.202443	-1.864544
H		-1.763523	-3.459786	-2.845841
C		-4.468725	-0.357053	-0.940609
H		-4.574993	0.436676	-0.209288
C		-4.235533	-2.408377	-2.792653
H		-4.139304	-3.216972	-3.513513
C		-5.485169	-0.570904	-1.867860
H		-6.367390	0.062434	-1.856075
C		-5.368271	-1.600155	-2.803745
H		-6.154878	-1.772907	-3.532571
N		-1.136293	-1.884860	0.084851
H		0.036605	-3.095819	-1.146955

H	-2.213596	-3.993500	-1.225821
C	-0.095852	0.293877	0.634342
C	-1.540899	0.513697	0.005765
H	-2.701404	-0.964519	1.090875
Cl	4.603270	-2.986559	-1.208398
H	-1.380654	0.693825	-1.060343
C	0.907362	0.760162	-0.434143
C	1.313364	2.153707	1.392126
C	0.304424	1.128547	1.867641
H	0.807781	0.526648	2.631572
H	-0.525809	1.633163	2.361043
O	1.781032	3.071117	2.026676
N	1.650699	1.834372	0.061069
O	1.014467	0.321913	-1.565366
C	2.618917	2.557232	-0.709612
C	3.587180	1.862121	-1.439313
C	2.589158	3.954725	-0.719091
C	4.522672	2.573513	-2.188643
H	3.604013	0.779526	-1.424212
C	3.536814	4.654778	-1.464124
H	1.841666	4.482748	-0.140391
C	4.501956	3.968847	-2.202621
H	5.271531	2.032183	-2.758566
H	3.516135	5.740292	-1.467566
H	5.235385	4.518779	-2.784557
C	-2.338375	1.682300	0.545887
C	-2.499862	2.829263	-0.244937
C	-2.937778	1.661156	1.817899
C	-3.236173	3.923029	0.211825
H	-2.050790	2.860477	-1.234428
C	-3.675280	2.754495	2.274323
H	-2.819273	0.788601	2.453072
C	-3.828160	3.888558	1.474402
H	-3.349521	4.798488	-0.421040
H	-4.131287	2.718565	3.259572
H	-4.403865	4.736994	1.832358

Zero-point correction = 0.528551 Hartree
ZPE is included in the following quantities:
Electronic energy = -2160.956677 Hartree
Internal energy = -2160.924698 Hartree
Enthalpies= -2160.923754 Hartree
Gibbs energy = -2161.021246 Hartree.
Number of imaginary frequencies: 0

- **4’f**

Coordinates (Angstroms)

Atom X Y Z

C	1.919084	-0.598164	2.565584
C	1.946800	0.686436	0.379582
C	3.408696	-0.207160	2.547342
H	1.454145	-0.250629	3.496514
C	2.896029	1.686424	1.012530
C	3.617364	1.227346	2.129958
H	3.846009	-0.399297	3.531834
C	3.087384	2.991662	0.555669
H	2.545529	3.351013	-0.312549
C	4.499649	2.088295	2.784279
H	5.046412	1.733958	3.654660
C	3.981405	3.842774	1.209699
H	4.121373	4.856993	0.847588
C	4.684424	3.394130	2.326644
H	5.373810	4.057005	2.841377
N	1.199492	-0.007150	1.433914
H	1.848868	-1.696081	2.553011
H	3.934433	-0.864542	1.839950
C	-0.401838	0.266251	-0.304045
C	0.808531	1.260760	-0.485919
C	0.095365	-0.734196	0.851318
C	0.397523	-2.167664	0.377200
C	-1.007167	-1.010148	1.939892
C	-0.469516	-3.062568	1.024817
H	2.559188	-0.014365	-0.220205
O	-1.502853	-0.206359	2.701667
N	-1.312989	-2.353637	1.886877
H	-1.980083	-2.767622	2.521882
C	1.359410	-2.668780	-0.492523
C	-0.418276	-4.434301	0.810474
H	-1.100573	-5.111330	1.314404
C	1.422732	-4.051206	-0.706209
C	0.545936	-4.929431	-0.073061
H	0.615632	-5.993893	-0.265074
H	2.050910	-2.018247	-1.013063
Cl	2.639210	-4.688504	-1.806925
H	0.484060	2.169460	0.028232
C	-0.986740	-0.449879	-1.533912
H	-0.856646	0.167974	-2.429309
H	-0.578204	-1.433745	-1.754670
C	-1.638005	1.086469	0.159175
C	-2.480796	-0.544491	-1.270193
O	-3.275748	-1.272277	-1.823422
O	-1.632447	2.136737	0.755319
N	-2.793117	0.422229	-0.297600
C	-4.121574	0.780877	0.099771

C	-4.378619	1.068722	1.444444
C	-5.146479	0.840900	-0.849784
C	-5.669080	1.426297	1.830840
H	-3.576393	1.011686	2.170189
C	-6.434344	1.189822	-0.445738
H	-4.941045	0.601613	-1.885303
C	-6.699741	1.487019	0.891504
H	-5.867352	1.654581	2.873765
H	-7.230434	1.231817	-1.183023
H	-7.703807	1.762844	1.199826
C	1.141381	1.630120	-1.918484
C	1.969366	0.854112	-2.743166
C	0.573684	2.793803	-2.462768
C	2.226777	1.231301	-4.061856
H	2.427334	-0.052386	-2.359459
C	0.824857	3.171399	-3.782020
H	-0.070979	3.407817	-1.838875
C	1.655484	2.390978	-4.586973
H	2.875680	0.616440	-4.678726
H	0.374962	4.077397	-4.177511
H	1.857036	2.683755	-5.613010

Zero-point correction = 0.527438 Hartree
ZPE is included in the following quantities:
Electronic energy = -2160.946851 Hartree
Internal energy = -2160.914584 Hartree
Enthalpies= -2160.913640 Hartree
Gibbs energy = -2161.012267 Hartree.
Number of imaginary frequencies: 0

- **5f**

Coordinates (Angstroms)

Atom	X	Y	Z
C	4.061611	0.197077	0.170194
C	2.767597	0.249232	0.703864
C	2.553083	0.078311	2.062111
C	3.669636	-0.156783	2.873649
C	4.958637	-0.221654	2.344634
C	5.170050	-0.042472	0.971905
C	2.715464	0.697608	-1.639101
C	1.764818	0.486274	-0.410452
H	1.551337	0.134798	2.475759
H	5.799441	-0.408267	3.002922
H	6.173336	-0.087098	0.560396
N	4.000985	0.439903	-1.211714
H	4.799294	0.557496	-1.819278
O	2.407610	1.083915	-2.755093
C	1.465628	2.945607	-0.013507

C	-0.283055	1.451423	-1.072980
C	0.737817	3.997070	-0.864765
H	1.408602	3.229859	1.044841
C	-1.298332	2.556242	-0.833771
C	-0.757348	3.854568	-0.738539
H	1.063051	4.999644	-0.569897
C	-2.685195	2.394658	-0.778307
H	-3.135203	1.414311	-0.872255
C	-1.603683	4.948653	-0.552354
H	-1.175431	5.944259	-0.467555
C	-3.525873	3.496870	-0.604203
H	-4.600664	3.348602	-0.561094
C	-2.986474	4.775510	-0.483873
H	-3.636452	5.633785	-0.341635
N	0.878562	1.610109	-0.188526
H	2.528864	2.941199	-0.285770
H	1.032261	3.860607	-1.914582
C	0.757897	-0.723489	-0.562146
C	-0.652467	-0.052167	-0.884013
H	0.667583	-1.093664	0.458936
C	-1.484654	-0.625499	-2.042088
C	-1.532765	-0.305181	0.356968
C	-2.755238	-1.188787	-1.432234
C	1.209505	-1.910650	-1.393164
C	1.410428	-3.136084	-0.738968
C	1.426389	-1.861644	-2.782053
C	1.818019	-4.273411	-1.436415
H	1.247088	-3.198312	0.333807
C	1.831396	-2.999174	-3.481001
H	1.297411	-0.928420	-3.318179
C	2.028815	-4.208638	-2.813217
H	1.967152	-5.207480	-0.902776
H	1.992370	-2.936773	-4.553350
O	-1.253107	-0.041895	1.506872
N	-2.710549	-0.961699	-0.043229
O	-3.658992	-1.739893	-2.020473
C	-3.748401	-1.352322	0.863388
C	-4.344309	-2.611051	0.736545
C	-4.161891	-0.469709	1.866576
C	-5.359663	-2.980815	1.617100
H	-4.026702	-3.282337	-0.050970
C	-5.171670	-0.856360	2.746195
H	-3.689885	0.500001	1.961497
C	-5.774572	-2.108968	2.624553
H	-5.824572	-3.956582	1.513826
H	-5.487774	-0.171725	3.527405

H	-6.563109	-2.403679	3.310417
H	-1.761515	0.134298	-2.779806
H	-0.986814	-1.429197	-2.587981
H	0.024544	1.531249	-2.131979
H	2.342566	-5.091869	-3.361760
Cl	3.442291	-0.372670	4.605867

Zero-point correction = 0.527924 Hartree
ZPE is included in the following quantities:
Electronic energy = -2160.951755 Hartree
Internal energy = -2160.919534 Hartree
Enthalpies= -2160.918590 Hartree
Gibbs energy = -2161.017726 Hartree.
Number of imaginary frequencies: 0

- **5'f**

Coordinates (Angstroms)

Atom	X	Y	Z
C	1.855217	2.660059	1.349218
C	0.180128	1.524229	-0.188418
C	1.490508	3.884482	0.496431
H	1.679453	2.879281	2.409893
C	-0.654522	2.795992	-0.198379
C	0.016538	3.972222	0.189062
H	1.840517	4.793469	0.995037
C	-1.974009	2.887110	-0.648833
H	-2.498463	2.009582	-1.008211
C	-0.666031	5.190698	0.192667
H	-0.144048	6.090431	0.508617
C	-2.643727	4.111967	-0.658914
H	-3.671299	4.158291	-1.006700
C	-1.995655	5.265098	-0.221102
H	-2.515951	6.218225	-0.214455
N	1.061541	1.488993	0.978641
H	2.930795	2.461813	1.230162
H	2.037745	3.825085	-0.455792
C	0.384449	-0.706700	0.853172
C	-0.491380	0.097037	-0.196340
H	-0.139142	-0.538696	1.797954
C	-0.628069	-0.482854	-1.614202
C	-1.930209	0.037696	0.356420
C	-2.075054	-0.913077	-1.776927
C	0.501553	-2.208057	0.684273
C	-0.280531	-3.014745	1.528617
C	1.318483	-2.848580	-0.262644
C	-0.254637	-4.405244	1.431552
H	-0.914579	-2.540412	2.272549
C	1.348803	-4.241059	-0.358284

H	1.948880	-2.267432	-0.924920
C	0.562368	-5.024836	0.485658
H	-0.869828	-5.002274	2.098324
H	1.990812	-4.711358	-1.097459
O	-2.286879	0.384513	1.459658
N	-2.768466	-0.558641	-0.605148
O	-2.561299	-1.457753	-2.742942
C	-4.164317	-0.806312	-0.393694
C	-4.729190	-2.013583	-0.818376
C	-4.953729	0.161376	0.237098
C	-6.088641	-2.244028	-0.613748
H	-4.114884	-2.752826	-1.315287
C	-6.309751	-0.086966	0.443011
H	-4.507434	1.086823	0.577448
C	-6.882545	-1.286096	0.017839
H	-6.525085	-3.180179	-0.948579
H	-6.918329	0.663744	0.937996
H	-7.940069	-1.473043	0.178259
H	-0.410162	0.256164	-2.392675
H	0.006691	-1.350516	-1.805534
C	1.655829	0.168602	1.078195
C	2.921273	-0.080675	0.249859
C	2.171117	-0.101791	2.532077
C	3.965613	-0.448573	1.117129
H	0.769397	1.556097	-1.127749
O	1.528425	-0.019234	3.556234
N	3.495979	-0.477353	2.433549
H	4.045780	-0.712859	3.246619
C	3.186706	0.053927	-1.108616
C	5.251956	-0.709629	0.660577
H	6.046020	-0.998389	1.341938
C	4.482814	-0.203831	-1.573132
C	5.506566	-0.585715	-0.708985
H	6.498135	-0.782046	-1.100370
H	2.416690	0.337468	-1.815700
H	0.587334	-6.107750	0.407603
Cl	4.819890	-0.043456	-3.293849

Zero-point correction = 0.527574 Hartree
ZPE is included in the following quantities:
Electronic energy = -2160.942797 Hartree
Internal energy = -2160.910365 Hartree
Enthalpies= -2160.909421 Hartree
Gibbs energy = -2161.009305 Hartree.
Number of imaginary frequencies: 0

3. Experimental Section

3.1. General procedure for the preparation of cycloadducts **4–5**

A mixture of **1** (1.0 mmol), isatin **2a-c** or acenaphthenequinone **2d** and tetrahydroisoquinoline **3** (1.2 mmol) was refluxed in methanol (5 mL) for 4h. After completion of the reaction (TLC), the solvent was removed under vacuum. The residue was chromatographed on silica gel employing ethyl acetate-cyclohexane (3:7 v/v) as eluent to obtain the pure products **4–5**.

3.2. Spectroscopic data of compounds 4 and 5

(1'R,2'R*,3R*,10b'R*)-1',1''-diphenyl-1',5',6',10b'-tetrahydrodispiro[indoline-3,3'-pyrrolo[2,1-a]isoquinoline-2',3''-pyrrolidine]-2,2'',5''-trione (**4a**).*
White solid (0.26 g, 51%); mp 262-264°C; ^{1}H NMR: δ 2.47 (d, 1H, H-4″, J = 18.9Hz), 2.72-3.2 (m, 5H, H-4″, H-5' and H-6'), 4.38 (d, 1H, H-1', J = 9.3 Hz), 5.57 (d, 1H, H-10b', J = 9.3 Hz), 6.71-7.49 (m, 18H, Ar-H); ^{13}C NMR: δ 29.8, 37.4, 42.9, 55.7, 61.5, 63.2, 77.2, 110.1, 123.4, 124.8, 125.6, 126.3, 126.5, 127.6, 128, 128.5, 128.7, 128.8, 129.6, 130.3, 131.5, 134.3, 138.6, 141.7, 173.7, 177.7; Anal Calcd for $C_{34}H_{27}N_3O_3$: C, 77.70; H, 5.18; N, 7.99 %; found: C, 77.81; H, 5.1; N, 8.06 %.

(1'S,2'S*,3S*,10b'S*)-1'',2'-diphenyl-6',10b'-dihydro-2'H,5'H-dispiro[indoline-3,3'-pyrrolo[2,1-a]isoquinoline-1',3''-pyrrolidine]-2,2'',5''-trione (**5a**).*
White solid (0.17g, 34%); mp 212-214°C; ^{1}H NMR: δ 2.65-3.12 (m, 4H, H-5' and H-6'), 3.25 (d, 1H, H-4″, J = 19 Hz), 3.35 (d, 1H, H-4″, J = 19 Hz), 4.64 (s, 1H, H-2'), 5.74 (s, 1H, H-10b'), 6.71-7.64 (m, 18H, Ar-H); ^{13}C NMR: δ 30.3, 40.4, 41.8, 56.1, 61.7, 70.1, 74.6, 109.7, 123.2, 123.8, 125.2, 125.8, 126.2, 127, 127.7, 128.7, 128.8, 129.6, 129.9, 130.4, 131.8, 133.6, 134.1, 135.8, 141.3, 174.7, 178.5, 179.7; Anal Calcd for $C_{34}H_{27}N_3O_3$: C, 77.70; H, 5.18; N, 7.99 %; found: C, 77.75; H, 5.17; N, 8.00 %.

(1'R,2'R*,3R*,10b'R*)-1''-phenyl-1'-(p-tolyl)-1',5',6',10b'-tetrahydrodispiro[indoline-3,3'-pyrrolo [2,1-a]isoquinoline-2',3''-pyrrolidine]-2,2'',5''-trione (**4b**).*
White solid (0.3g, 57%); mp 252-254°C; ^{1}H NMR: δ 2.43 (s, 3H, CH_3), 2.52 (d, 1H, H-4″, J = 18.9 Hz), 2.72-3.1 (m, 5H, H-4″, H-5' and H-6'), 4.36 (d, 1H, H-1', J = 9.6 Hz), 5.55 (d, 1H, H-10b', J = 9.6 Hz), 6.76-7.43 (m, 17H, Ar-H), 7.83 (bs, 1H, NH); ^{13}C NMR: δ 20.6, 29.3, 36.8, 42.4, 54.9, 61.1, 62.5, 77.2, 109.4, 122.9, 124.3, 124.8, 125, 125.7, 126.3, 126, 127.1, 128, 129.7, 133.9, 135, 137.6, 141.1, 173.2, 177.2; Anal Calcd for $C_{35}H_{29}N_3O_3$: C, 77.90; H, 5.42; N, 7.79 %; found: C, 78.06; H, 5.48; N, 7.88 %.

(1'S,2'S*,3S*,10b'S*)-1''-phenyl-2'-(p-tolyl)-6',10b'-dihydro-2'H,5'H-dispiro[indoline-3,3'-pyrrolo[2,1-a]isoquinoline-1',3''-pyrrolidine]-2,2'',5''-trione (**5b**).*
White solid (0.16g, 31%); mp 208-210°C; ^{1}H NMR: δ 2.27 (s, 3H, CH_3), 2.71-3. 2 (m, 4H, H-5' and H-6'), 3.31 (dd, 2H, H-4″), 4.62 (s, 1H, H-2'), 5.73 (s, 1H, H-10b'), 6.72-7.65 (m, 17H, Ar-H); 7.71 (bs, 1H, NH); ^{13}C NMR: δ 20.4, 29.9, 39.9, 41.3, 55.6, 61, 69.6, 74.1, 109.1, 122.7, 123.2, 124.7, 125.3, 125.7, 126.4, 127.3, 127.9, 128.4, 129, 129.3, 129.8, 130, 131.4, 133.8, 135.3, 137.4, 140.8, 174.3, 178, 179.3; Anal Calcd for $C_{35}H_{29}N_3O_3$: C, 77.90; H, 5.42; N, 7.79 %; found: C, 78.02; H, 5.50; N, 7.59 %.

(1'R,2'R*,3R*,10b'R*)-1'-(p-anisyl)-1''-phenyl-1',5',6',10b'-tetrahydrodispiro[indoline-3,3'-pyrrolo [2,1-a]isoquinoline-2',3''-pyrrolidine]-2,2'',5''-trione (**4c**).*
White solid (0.32g, 59%); mp 232-234°C; ^{1}H NMR: δ 2.56 (d, 1H, H-4″, J = 18.9 Hz), 2.74-3.18 (m, 5H, H-4″, H-5' and H-6'), 3.86 (s, 3H, OCH_3), 4.32 (d, 1H, H-1', J = 9.9 Hz), 5.55 (d, 1H, H-10b', J = 9.9 Hz), 6.74-7.4 (m, 17H, Ar-H), 7.88 (bs, 1H, NH); ^{13}C NMR: δ 29.8, 37.3, 42.9, 55.1, 55.3, 61.5, 63.2, 77.2, 110, 123.4, 124.8, 125.6, 126.3, 126.5, 127.6, 128.5,

128.7, 128.8, 130.2, 131.5, 159.2, 173.8, 177.9; Anal Calcd for $C_{35}H_{29}N_3O_4$: C, 75.66; H, 5.26; N, 7.56 %; found: C, 75.72; H, 5.36; N, 7.45 %.

(1'S,2'S*,3S*,10b'S*)-2'-(p-anisyl)-1''-phenyl-6',10b'-dihydro-2'H,5'H-dispiro[indoline-3,3'-pyrrolo[2,1-a]isoquinoline-1',3''-pyrrolidine]-2,2'',5''-trione (**5c**).*
White solid (0.15g, 28%); mp 200-202°C; ^{1}H NMR: δ 2.6-3.2 (m, 4H, H-5' and H-6'), 3.32 (dd, 2H, H-4''), 3.73 (s, 3H, OCH_3), 4.59 (s, 1H, H-2'), 5.74 (s, 1H, H-10b'), 6.73-7.65 (m, 17H, Ar-H); ^{13}C NMR: δ 30.3, 40.4, 41.8, 55.1, 56.1, 61.1, 70, 74.8, 109.6, 114.2, 123.2, 123.7, 125.2, 125.8, 126.2, 127, 128.4, 128.9, 129.6, 129.9, 131.7, 131.8, 135.8, 141.3, 159.3, 174.8, 179.8; Anal Calcd for $C_{35}H_{29}N_3O_4$: C, 75.66; H, 5.26; N, 7.56 %; found: 75.78; H, 5.35; N, 7.33 %.

(1'R,2'R*,3R*,10b'R*)-1'-(p-chlorophenyl)-1''-phenyl-1',5',6',10b'-tetrahydrodispiro [indoline-3,3'-pyrrolo [2,1-a]isoquinoline-2',3''-pyrrolidine]-2,2'',5''-trione (**4d**).*
White solid (0.3g, 54%); mp 252-254°C; ^{1}H NMR: δ 2.44 (d, 1H, H-4'', J = 18.9 Hz), 2.71-3.18 (m, 5H, H-4'', H-5' and H-6'), 4.35 (d, 1H, H-1', J = 9.6 Hz), 5.55 (d, 1H, H-10b', J = 9.6 Hz), 6.68-7.49 (m, 17H, Ar-H), 8.03 (bs, 1H, NH); ^{13}C NMR: δ 29.7, 37.3, 42.9, 55.01, 61.3, 63.5, 77.2, 110.2, 123.5, 124.6, 125.6, 126.5, 127.4, 128.6, 128.9, 129.8, 130.4, 131.3, 134, 134.3, 137.4, 141.7, 173.4, 177.6; Anal Calcd for $C_{34}H_{26}ClN_3O_3$: C, 72.92; H, 4.68; N, 7.50 %; found: C, 73.08; H, 4.78; N, 7.59 %.

(1'S,2'S*,3S*,10b'S*)-2'-(p-chlorophenyl)-1''-phenyl-6',10b'-dihydro-2'H,5'H-dispiro[indoline-3,3'-pyrrolo[2,1-a]isoquinoline-1',3''-pyrrolidine]-2,2'',5''-trione (**5d**).*
White solid (0.14g, 26%); mp 208-210°C; ^{1}H NMR: δ 2.72-3.18 (m, 4H, H-5' and H-6'), 3.26 (dd, 2H, H-4''), 4.61 (s, 1H, H-2'), 5.77 (s, 1H, H-10b'), 6.72-7.65 (m, 17H, Ar-H); ^{13}C NMR: δ 30.3, 40.3, 41.7, 55.9, 61.3, 70.1, 74.7, 109.8, 110.1, 123.3, 123.5, 123.8, 124.5, 125.2, 125.6, 125.9, 126.2, 126.5, 127.1, 127.4, 128.5, 128.6, 129.1, 129.9, 130.4, 131.7, 131.9, 134.4, 141.2, 174.4, 179.5; Anal Calcd for $C_{34}H_{26}ClN_3O_3$: C, 72.92; H, 4.68; N, 7.50 %; found: C, 73.04; H, 4.77; N, 7.41 %.

(1'R,2'R*,3R*,10b'R*)-1''-phenyl-1'-(pyridin-2-yl)-1',5',6',10b'-tetrahydrodispiro [indoline-3,3'-pyrrolo [2,1-a]isoquinoline-2',3''-pyrrolidine]-2,2'',5''-trione (**4e**).*
White solid (0.24g, 48%); mp 260-262°C; ^{1}H NMR: δ 2.36 (d, 1H, H-4'', J = 18.6 Hz), 2.69-3.12 (m, 5H, H-4'', H-5' and H-6'), 4.64 (d, 1H, H-1', J = 9.6 Hz), 5.67 (d, 1H, H-10b', J = 9.6 Hz), 6.64-8.73 (m, 18H, Ar-H and NH); ^{13}C NMR: δ 29.6, 36.6, 43.1, 57.3, 60.8, 62.1, 77.2, 110, 122.7, 123.3, 124.5, 125.8, 126.6, 127.9, 128.5, 128.7, 128.8, 130.2, 131.6, 134, 134.6, 137.6, 137.7, 141.6, 149.9, 158.8, 173.7, 177.1, 177.4; Anal Calcd for $C_{33}H_{26}N_4O_3$: C, 75.27; H, 4.98; N, 10.64 %; found: C, 75.14; H, 5.08; N, 10.72 %.

(1'R,2'R*,3R*,10b'R*)-1''-phenyl-1'-(p-chlorophenyl)-1',5',6',10b'-tetrahydrodispiro [indoline-3,3'-pyrrolo [2,1-a]isoquinoline-2',3''-pyrrolidine]-2,2'',5''-trione (**4f**).*
White solid (0.38g, 68%); mp 252-254°C; ^{1}H NMR: δ 2.52 (d, 1H, H-4'', J = 18.6 Hz), 2.74-3.2 (m, 5H, H-4'', H-5' and H-6'), 4.4 (d, 1H, H-1', J = 9.6 Hz), 5.58 (d, 1H, H-10b', J = 9.6 Hz), 6.73-7.52 (m, 17H, Ar-H), 8.46 (bs, 1H, NH); ^{13}C NMR: δ 29.6, 37.3, 43, 55.7, 63.3, 77.1, 111.2, 124.7, 125.7, 126.2, 126.5, 126.8, 127.7, 128.2, 128.6, 128.8, 129, 129.7, 130.4, 131.3, 134.2, 137.7, 138.4, 140.3, 177.4, 177.7; Anal Calcd for $C_{35}H_{30}ClN_3O_3$: C, 72.97; H, 5.25; N, 7.29 %; found: C, 73.08; H, 5.11; N, 7.39 %.

(1'S,2'S*,3S*,10b'S*)-1''-phenyl-2'-(p-chlorophenyl)-6',10b'-dihydro-2'H,5'H-dispiro[indoline-3,3'-pyrrolo[2,1-a]isoquinoline-1',3''-pyrrolidine]-2,2'',5''-trione (**5f**).*
White solid (0.09g, 17%); mp 204-206 °C; ^{1}H NMR: δ 2.71-3.11 (m, 4H, H-5' and H-6'), 3.28 (dd, 2H, H-4''), 4.54 (s, 1H, H-2'), 5.77 (s, 1H, H-10b'), 6.73-7.50 (m, 18H, Ar-H and NH); ^{13}C NMR: δ 30.2, 37.2, 43, 55.8, 61.3, 69.9, 74.9, 111, 123.7, 124.5, 125.5, 125.7, 126.2, 126.6, 127.3, 127.6, 128.6, 129.9, 129, 129.1, 130, 131.6, 132, 134.1, 134.6, 139.8, 174.2, 179.2; Anal Calcd for $C_{35}H_{30}ClN_3O_3$: C, 72.97; H, 5.25; N, 7.29 %; found: C, 73.04; H, 4.97; N, 7.36 %.

(1'R,2'R*,3R*,10b'R*)-6-chloro-1''-phenyl-1'-(p-tolyl)-1',5',6',10b'-tetrahydrodispiro [indoline-3,3'-pyrrolo[2,1-a]isoquinoline-2',3''-pyrrolidine]-2,2'',5''-trione (**4g**).*
White solid (0.39g, 69%); mp 234-236 °C; ^{1}H NMR: δ 2.43 (s, 3H, CH_3), 2.65 (d, 1H, H-4'', J = 18.9 Hz), 2.77-3.15 (m, 5H, H-4'', H-5' and H-6'), 4.34 (d, 1H, H-1', J = 9.9 Hz), 5.58 (d, 1H, H-10b', J = 9.9 Hz), 6.73-7.44 (m, 16H, Ar-H); ^{13}C NMR: δ 21.2, 29.7, 37.1, 43.1, 54.9, 61.1, 63.2, 76.1, 109.3, 122.8, 124.3, 124.8, 125, 125.7, 126.3, 127.1, 128, 128.2, 129.7, 132.9, 135, 137.6, 141.1, 173.2, 177.7; Anal Calcd for $C_{35}H_{28}ClN_3O_3$: C, 73.23; H, 4.92; N, 7.32 %; found: C, 73.13; H, 5.02; N, 7.42 %.

(1'S,2'S*,3S*,10b'S*)-6-chloro-1''-phenyl-2'-(p-tolyl)-6',10b'-dihydro-2'H,5'H-dispiro[indoline-3,3'-pyrrolo[2,1-a]isoquinoline-1',3''-pyrrolidine]-2,2'',5''-trione (**5g**).*
White solid (0.08g, 14%); mp 200-202°C; ^{1}H NMR: δ 2.28 (s, 3H, CH_3), 2.89-3.17 (m, 4H, H-5' and H-6'), 3.30 (dd, 2H, H-4''), 4.57 (s, 1H, H-2'), 5.79 (s, 1H, H-10b'), 6.68-7.63 (m, 16H, Ar-H), ^{13}C NMR: δ 20.5, 30.3, 39.8, 41.4, 55.4, 61.7, 69.3, 74.3, 110.5, 123.2, 125, 125.5, 125.7, 126.7, 128.1, 128.5, 129.2, 129.5, 129.8, 131.2, 137.8, 139.6, 140.8, 174.1, 179.1, Anal Calcd for $C_{35}H_{28}ClN_3O_3$: C, 73.23; H, 4.92; N, 7.32 %; found: C, 73.19; H, 5.06; N, 7.44 %.

(1'R,2'R*,3R*,10b'R*)-1'-(p-anisyl)-6-chloro-1''-phenyl-1',5',6',10b'-tetrahydrodispiro [indoline-3,3'-pyrrolo[2,1-a]isoquinoline-2',3''-pyrrolidine]-2,2'',5''-trione (**4h**).*
White solid (0.44g, 76%); mp 266-268°C; ^{1}H NMR: δ 2.58 (d, 1H, H-4'', J = 18.9 Hz), 2.73-3.25 (m, 5H, H-4'', H-5' and H-6'), 3.89 (s, 3H, OCH_3), 4.34 (d, 1H, H-1', J = 9.3 Hz), 5.5 (d, 1H, H-10b', J = 9.3 Hz), 6.76-7.44 (m, 16H, Ar-H), 8.57 (bs, 1H, NH); ^{13}C NMR: δ 29.1, 36.7, 42.5, 54.8, 60.9, 62.8, 76.1, 110.7, 114.6, 124.2 125.2, 125.7, 125.9, 127.3, 128.1, 128.2, 128.4, 128.5, 129.9, 130.9, 133.6, 139.8, 158.9, 173.2, 177.3; Anal Calcd for $C_{35}H_{28}ClN_3O_4$: C, 71.24; H, 4.78; N, 7.12 %; found: C, 71.32; H, 4.87; N, 7.22 %.

(1'R,2'R*,3R*,10b'R*)-6-chloro-1''-phenyl-1'-(pyridin-2-yl)-1',5',6',10b'-tetrahydrodispiro [indoline-3,3'-pyrrolo [2,1-a]isoquinoline-2',3''-pyrrolidine]-2,2'',5''-trione (**4i**).*
White solid (0.38g, 68%); mp 262-263°C; ^{1}H NMR: δ 2.55 (d, 1H, H-4'', J = 18.9 Hz), 2.73-3.14 (m, 5H, H-4'', H-5' and H-6'), 4.34 (d, 1H, H-1', J = 9.6 Hz), 5 (d, 1H, H-10b', J = 9.6 Hz), 6.76-7.44 (m, 16H, Ar-H), 8.57 (bs, 1H, NH); ^{13}C NMR: δ 29.6, 36.7, 43.1, 57.3, 60.8, 62.8, 76.5, 110.7, 122.7, 123.3, 124.2, 124.5, 125.2, 125.7, 126.5, 126.6, 127.9, 128.5, 128.8, 130.2, 131.6, 134.6, 137.6, 137.8, 141.6, 149.9, 160.8, 173.2, 177.2, 177.4; Anal Calcd for $C_{33}H_{25}ClN_4O_3$: C, 70.65; H, 4.49; N, 9.99 %; found: C, 70.72; H, 4.59; N, 10.05 %.

(1'R,2'R*,3R*,10b'R*)-6-bromo-1',1''-diphenyl-1',5',6',10b'-tetrahydrodispiro[indoline-3,3'-pyrrolo[2,1-a]isoquinoline-2',3''-pyrrolidine]-2,2'',5''-trione (**4j**).*
White solid (0.38g, 60%); mp 232-234°C; ^{1}H NMR: δ 2.45 (d, 1H, H-4'', J = 18.9Hz), 2.7-3.2 (m, 5H, H-4'', H-5' and H-6'), 4.35 (d, 1H, H-1', J = 9.6 Hz), 5.49 (d, 1H, H-10b', J = 9.6

Hz), 6.69-7.75 (m, 17H, Ar-H); ^{13}C NMR: δ 29.7, 37.3, 43, 54.9, 61.4, 63.7, 77.2, 111.5, 116.5, 124.5, 125.7, 126.2, 126.5, 126.6, 127.1, 128, 128.6, 128.9, 129, 130.1, 130.5, 131.3, 131.7, 133.4, 134.2, 137.2, 137.5, 140.5, 173.2, 176.9, 177.5; Anal Calcd for $C_{34}H_{26}BrN_3O_3$: C, 67.56; H, 4.34; N, 6.95%; found: C, 67.68; H, 4.22; N, 7.01 %.

.

(1'R,2'R*,3R*,10b'R*)-6-bromo-1''-phenyl-1'-(p-tolyl)-1',5',6',10b'-tetrahydrodispiro [indoline-3,3'-pyrrolo[2,1-a]isoquinoline-2',3''-pyrrolidine]-2,2'',5''-trione (**4k**).*

White solid (0.45g, 75%); mp 245-247 °C; ^{1}H NMR: δ 2.42 (s, 3H, CH_3), 2.51 (d, 1H, H-4″, *J* = 18.9 Hz), 2.68-3.18 (m, 5H, H-4″, H-5' and H-6'), 4.33 (d, 1H, H-1', *J* = 9.2 Hz), 5.51 (d, 1H, H-10b', *J* = 9.2 Hz), 6.71-7.55 (m, 16H, Ar-H), 8.16 (bs, 1H, NH); ^{13}C NMR: δ 20.6, 29.2, 36.8, 42.5, 54.9, 61, 62.7, 76.6, 111.1, 115.8, 124.2, 125.1, 125.8, 128.1, 128.2, 128.5, 130, 130.9, 132.7, 137.3, 140.2, 173.2, 177.2; Anal Calcd for $C_{35}H_{28}BrN_3O_3$: C, 67.97; H, 4.56; N, 6.79 %; found: C, 68.02; H, 4.5; N, 6.71 %.

(1'S,2'S*,3S*,10b'S*)-6-bromo-1''-phenyl-2'-(p-tolyl)-6',10b'-dihydro-2'H,5'H-dispiro[indoline-3,3'-pyrrolo[2,1-a]isoquinoline-1',3''-pyrrolidine]-2,2'',5''-trione (**5k**).*

White solid (0.08g, 13%); mp 200-202°C; ^{1}H NMR: δ 2.26 (s, 3H, CH_3), 2.66-3.16 (m, 4H, H-5' and H-6'), 3.28 (dd, 2H, H-4″), 4.52 (s, 1H, H-2'), 5.7 (s, 1H, H-10b'), 6.57-7.78 (m, 16H, Ar-H), 8.1 (bs, 1H, NH);^{13}C NMR: δ 20.5, 28.8, 39.7, 41.3, 55.4, 61.2, 69.4, 74.2, 110.8, 115.4, 123.1, 125.4, 125.7, 126.6, 127.7, 128, 128.5, 129.1, 129.5, 129.6, 131.3, 132.1, 135.3, 137.7, 139.9, 174.1, 177.6, 179.1, Anal Calcd for $C_{35}H_{28}BrN_3O_3$: C, 67.97; H, 4.56; N, 6.79 %; found: C, 68.07; H, 4.66; N, 6.85 %.

(1'R,2'R*,3R*,10b'R*)-1'-(p-anisyl)-6-bromo-1''-phenyl-1',5',6',10b'-tetrahydrodispiro [indoline-3,3'-pyrrolo[2,1-a]isoquinoline-2',3''-pyrrolidine]-2,2'',5''-trione (**4l**).*

White solid (0.46g, 73%); mp 255-257°C; ^{1}H NMR: δ 2.57 (d, 1H, H-4″, *J* = 18.9 Hz), 2.72-3.2 (m, 5H, H-4″, H-5' and H-6'), 3.89 (s, 3H, OCH_3), 4.34 (d, 1H, H-1', *J* = 9.6 Hz), 5.5 (d, 1H, H-10b', *J* = 9.6 Hz), 6.7-7.57 (m, 16H, Ar-H), 8.75 (bs, 1H, NH); ^{13}C NMR: δ 29.2, 37.2, 42.5, 54.6, 54.9, 60.9, 62.8, 76.6, 110.7, 114.6, 124.2 125.2, 125.7, 125.9, 127.3, 128.1, 128.2, 128.4, 128.5, 129.8, 130.9, 133.9, 139.8, 160.9, 173.2, 177.8; Anal Calcd for $C_{35}H_{28}BrN_3O_4$: C, 66.25; H, 4.45; N, 6.62 %; found: C, 66.35; H, 4.51; N, 6.52 %.

(1'S,2'S*,3S*,10b'S*)-2'-(p-anisyl)-6-bromo-1''-phenyl-6',10b'-dihydro-2'H,5'H-dispiro [indoline-3,3'-pyrrolo[2,1-a]isoquinoline-1',3''-pyrrolidine]-2,2'',5''-trione (**5l**).*

White solid (0.07g, 12%); mp 210-212 °C; ^{1}H NMR: δ 2.65-3.27 (m, 4H, H-5' and H-6'), 3.28 (dd, 2H, H-4″), 3.74 (s, 3H, OCH_3), 4.48 (s, 1H, H-2'), 5.69 (s, 1H, H-10b'), 6.62-7.8(m, 16H, Ar-H), 7.93 (bs, 1H, NH); ^{13}C NMR: 30.3, 40.2, 40.9, 55.2, 55.9, 65.8, 75, 109.6, 114.3, 123.7, 125.2, 125.8, 126.2, 128.4, 128.9, 129.6, 129.9, 131.7, 135.8, 141.3, 159.5, 174.1, 179.1, Anal Calcd for $C_{35}H_{28}BrN_3O_4$: C, 66.25; H, 4.45; N, 6.62 %; found: C, 66.45; H, 4.54; N, 6.72 %.

(1'R,2'R*,3R*,10b'R*)-6-bromo-1''-phenyl-1'-(pyridin-2-yl)-1',5',6',10b'-tetrahydrodispiro [indoline-3,3'-pyrrolo [2,1-a]isoquinoline-2',3''-pyrrolidine]-2,2'',5''-trione (**4m**).*

White solid (0.41g, 69%); mp 260-262 °C; ^{1}H NMR: δ 2.58 (d, 1H, H-4″, *J* = 18.9 Hz), 2.61-3.17 (m, 5H, H-4″, H-5' and H-6'), 4.34 (d, 1H, H-1', *J* = 9.6 Hz), 5.5 (d, 1H, H-10b', *J* = 9.6 Hz), 6.7-7.57 (m, 16H, Ar-H), 8.75 (bs, 1H, NH); ^{13}C NMR: δ 29.2, 37.3, 43, 54.9, 61.4, 63.7, 77.2, 109.7, 114.3, 123.3, 123.7, 125.2, 125.7, 126.2, 127, 128.4, 128.9, 129.6, 130.2, 131.6, 131.7, 135.8, 141.6, 160.9, 174.8, 179.8; Anal Calcd for $C_{33}H_{25}BrN_4O_3$: C, 65.46; H, 4.16; N, 9.25 %; found C, 65.58; H, 4.26; N, 9.14 %.

(1'R,2'R*,3R*,10b'R*)-1',1''-diphenyl-1',5',6',10b'-tetrahydro-2H-dispiro[acenaphthylene-1,3'-pyrrolo[2,1-a]isoquinoline-2',3''-pyrrolidine]-2,2'',5''-trione (**4n**).*
White solid (0.31g, 55%); mp 258-260°C; ^{1}H NMR: δ 2.42 (dd, 2H, H-4''), 2.54-3 (m, 4H, H-5' and H-6'), 4.4 (d, 1H, H-1', *J* = 9.6 Hz), 5.6 (d, 1H, H-10b', *J* = 9.6 Hz), 6.58-8.12 (m, 20H, Ar-H); ^{13}C NMR: δ 29.8, 36.6, 42.9, 56.7, 61.7, 63.6, 77.9, 121.3, 124.2, 124.8, 125.7, 126, 126.3, 128, 128.4, 128.7, 128.8, 129, 129.7, 130.2, 130.6, 131.3, 131.5, 132.5, 134.6, 134.9, 138, 138.6, 143.3, 173.3, 178.3, 207.1; Anal Calcd for $C_{38}H_{28}N_2O_3$: C, 81.41; H, 5.03; N, 5.00 %; found C, 81.31; H, 4.93; N, 5.14 %.

(1'S,2'S*,3S*,10b'S*)-1'',2'-diphenyl-6',10b'-dihydro-2H,2'H,5'H-dispiro[acenaphthylene-1,3'-pyrrolo[2,1-a]isoquinoline-1',3''-pyrrolidine]-2,2'',5''-trione (**5n**).*
White solid (0.09g, 17%); mp 214-216°C; ^{1}H NMR: δ 2.54-3.03 (m, 4H, H-5' and H-6'), 3.23 (dd, 2H, H-4''), 4.85 (s, 1H, H-2'), 5.72 (s, 1H, H-10b'), 6.8-8.01 (m, 20H, Ar-H); ^{13}C NMR: δ 30.2, 40.7, 42, 57.3, 61.2, 71, 78.4, 121.1, 123.9, 125.4, 125.9, 126.2, 127.1, 127.8, 128.2, 128.4, 128.7, 128.8, 128.97, 129.8, 130.4, 131.7, 133.9, 132.4, 133.9, 135.7, 142.6, 174.8, 179.9, 206.2; Anal Calcd for $C_{38}H_{28}N_2O_3$: C, 81.41; H, 5.03; N, 5.00 %; found C, 81.29; H, 5.13; N, 5.09 %.

(1'S,2'S*,3S*,10b'S*))-1''-phenyl-2'-(p-tolyl)-6',10b'-dihydro-2H,2'H,5'H-dispiro [acenaphthylene-1,3'-pyrrolo[2,1-a]isoquinoline-1',3''-pyrrolidine]-2,2'',5''-trione (**5o**).*
White solid (0.09g, 17%); mp 200-202°C; ^{1}H NMR: δ 1.64 (s, 3H, CH_3), 2.27 (m, 4H, H-5' and H-6'), 3.31 (dd, 2H, H-4''), 4.62 (s, 1H, H-2'), 5.73 (s, 1H, H-10b'), 6.72-7.71 (m, 19H, Ar-H); ^{13}C NMR: δ 20.4, 29.9, 39.9, 41.3, 55.6, 61, 69.6, 74.1, 109.1, 122.7, 123.2, 124.7, 125.3, 125.7, 126.4, 127.3, 127.9, 128.4, 129, 129.3, 129.8, 130, 131.4, 133.8, 135.3, 137.4, 140.8, 174.3, 178, 206.1; Anal Calcd for $C_{39}H_{30}N_2O_3$: C, 81.51; H, 5.26; N, 4.87 %; found C, 81.61; H, 5.20; N, 4.94 %.

(1'R,2'R*,3R*,10b'R*)-1'-(p-anisyl)-1''-phenyl-1',5',6',10b'-tetrahydro-2H-dispiro [acenaphthylene-1,3'-pyrrolo[2,1-a]isoquinoline-2',3''-pyrrolidine]-2,2'',5''-trione (**4p**).*
White solid (0.3g, 52%); mp 251-253 °C; ^{1}H NMR: δ 2.54 (dd, 2H, H-4''), 2.63-3 (m, 4H, H-5' and H-6'), 3.9 (s, 3H, OCH_3), 4.68 (d, 1H, H-1', *J* = 9.6 Hz), 5.63 (d, 1H, H-10b', *J* = 9.6 Hz), 6.7-8.21 (m, 19H, Ar-H); ^{13}C NMR: δ 29.4, 36, 42.3, 54.8, 55.7, 61.2, 63.1, 79.3, 114.6, 120.8, 123.7, 124.2, 125.1, 125.8, 127.9, 128.2, 128.3, 128.5, 130, 130.1, 130.8, 131.1, 132, 134.1, 134.5, 137.7, 142.8, 158.5, 172.9, 177.9, 207.2; Anal Calcd for $C_{39}H_{30}N_2O_4$: C, 79.30; H, 5.12; N, 4.74 %; found 79. 50; H, 5; N, 4.81 %.

(1'S,2'S*,3S*,10b'S*)-2'-(p-anisyl)-1''-phenyl-6',10b'-dihydro-2H,2'H,5'H-dispiro [acenaphthylene-1,3'-pyrrolo[2,1-a]isoquinoline-1',3''-pyrrolidine]-2,2'',5''-trione (**5p**).*
White solid (0.09g,15%); mp 206-208°C; ^{1}H NMR: δ 2.6-3.07 (m, 4H, H-5' and H-6'), 3.38 (dd, 2H, H-4''), 3.75 (s, 3H, OCH_3), 4.88 (s, 1H, H-2'), 5.79 (s, 1H, H-10b'), 6.61-8.09 (m, 19H, Ar-H); ^{13}C NMR: δ 33.8, 40.1, 41.5, 54.5, 54.9, 56.7, 70.4, 78, 113.6, 114.2, 121.1, 123.4, 125.4, 125.7, 125.9, 127.7, 127.9, 128.3, 128.4, 128.5, 129.3, 129.8, 131.1, 131.4, 131.6, 131.9, 134.9, 158.5, 174.3, 179.6, 204.1; Anal Calcd for $C_{39}H_{30}N_2O_4$: C, 79.30; H, 5.12; N, 4.74 %; found 79.41; H, 5.22; N, 4.69 %.

(1'R,2'R*,3R*,10b'R*)-1'-(p-chlorophenyl)-1''-phenyl-1',5',6',10b'-tetrahydro-2H-dispiro [acenaphthylene-1,3'-pyrrolo[2,1-a]isoquinoline-2',3''-pyrrolidine]-2,2'',5''-trione (**4q**).*
White solid (0.33g, 57%); mp 248-250°C; ^{1}H NMR: δ 2.34 (dd, 2H, H-4″), 2.48-3 (m, 4H, H-5' and H-6'), 4.39 (d, 1H, H-1', *J* = 9.3 Hz), 5.55 (d, 1H, H-10b', *J* = 9.3 Hz), 6.6-8.14 (m, 19H, Ar-H); ^{13}C NMR: δ 29.3, 36.1, 42.3, 55.5, 61, 63.4, 79.3, 120.9, 123.6, 124.1, 125.2, 125.6, 125.8, 126, 128, 128.3, 128.5, 129.4, 130.1, 130.7, 130.9, 131.1, 132.1, 133.5, 134.1, 136.9, 137.3, 142.9, 172.5, 177.6, 206.7; Anal Calcd for $C_{38}H_{27}ClN_2O_3$: C, 76.70; H, 4.57; N, 4.71 %; found C, 76.85; H, 4.61; N, 4.8 %.

(1'S,2'S*,3S*,10b'S*)-2'-(p-chlorophenyl)-1''-phenyl-6',10b'-dihydro-2H,2'H,5'H-dispiro [acenaphthylene-1,3'-pyrrolo[2,1-a]isoquinoline-1',3''-pyrrolidine]-2,2'',5''-trione (**5q**).*
White solid (0.04g, 6%); mp 198-200°C; ^{1}H NMR: δ 2.43-3.11 (m, 6H, H-5', H-6' and H-4″), 4.6 (s, 1H, H-2'), 5.9 (s, 1H, H-10b'), 6.73-8.47 (m, 19H, Ar-H); ^{13}C NMR: δ 30.8, 40.3, 40.7, 56.1, 54.9, 68, 72.3, 76.5, 120.8, 123.2, 124.4, 124.7, 125.8, 126.5, 127.1, 127.9, 128.5, 129, 129.6, 129.9, 130.2, 131, 131.3, 131.7, 131.9, 132.7, 133.4, 136.3, 1371, 137.5, 140.6, 142.4, 169.6, 176.2, 209.3; Anal Calcd for $C_{38}H_{27}ClN_2O_3$: C, 76.70; H, 4.57; N, 4.71 %; found C, 76.82; H, 4.65; N, 4.81 %.

(1'S,2'S*,3S*,10b'S*)-1''-phenyl-2'-(pyridin-2-yl)-6',10b'-dihydro-2H,2'H,5'H-dispiro [acenaphthylene-1,3'-pyrrolo[2,1-a]isoquinoline-1',3''-pyrrolidine]-2,2'',5''-trione (**5r**).*
White solid (0.07g, 13%); mp 204-206 °C; ^{1}H NMR: δ 2.53-2.99 (m, 4H, H-5' and H-6'), 3.18 (dd, 2H, H-4″), 5 (s, 1H, H-2'), 5.6 (s, 1H, H-10b'), 6.55-8.13 (m, 19H, Ar-H); ^{13}C NMR: δ 30.1, 41.4, 42.4, 56.8, 52.9, 71.2, 76.3, 120.9, 121.4, 122.1, 122.7, 123.5, 125.5, 125.8, 126.3, 126.9, 128.3, 128.4, 128.5, 129, 129.7, 130.5, 130.9, 132.3, 133.5, 134.7, 135.2, 136.6, 137, 142.2, 149.1, 155, 175.2, 180.1, 204.2; Anal Calcd for $C_{37}H_{27}N_3O_3$: C, 79.13; H, 4.85; N, 7.48 %; found C, 79.23; H, 4.96; N, 7.4 %.

3.3. ^{1}H- and ^{13}C-NMR Spectra of compounds 4a-r and 5a-r (Fig. S1 to S36)

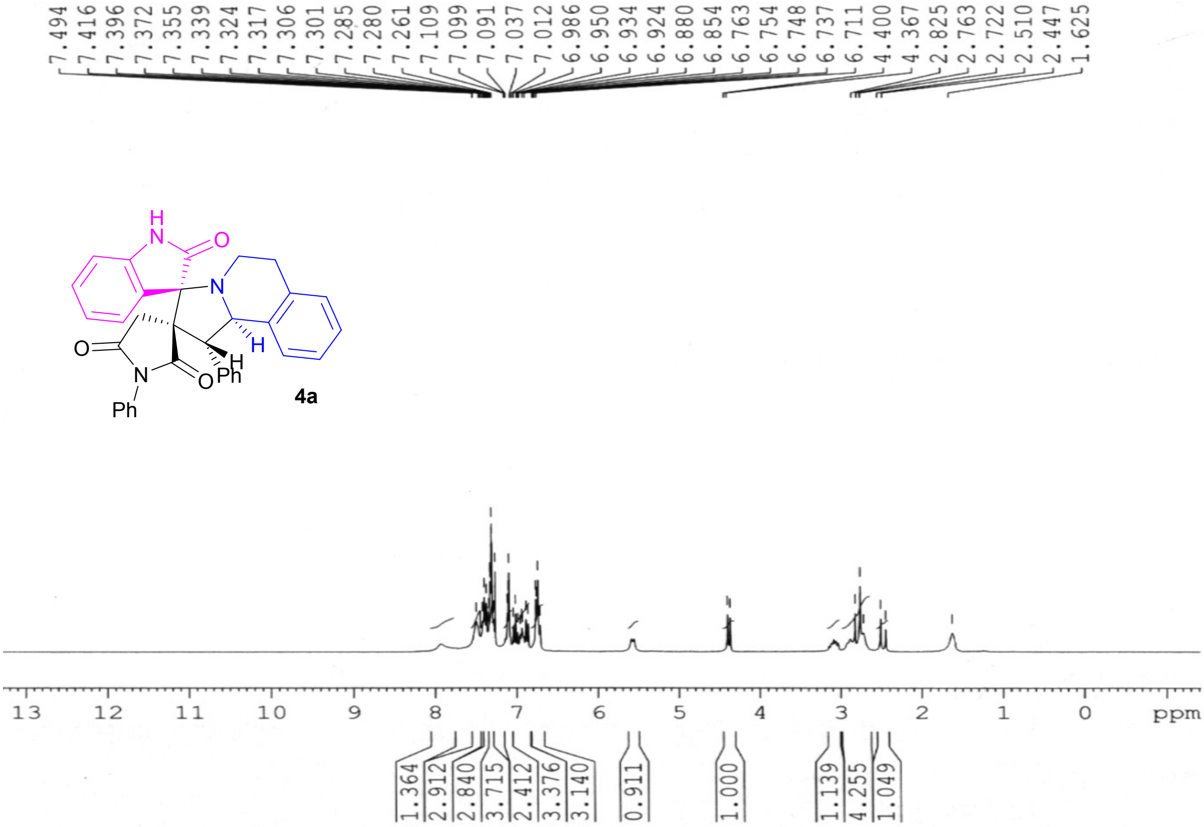

Fig. S1.^{1}H NMR spectrum of **4a** in $CDCl_3$

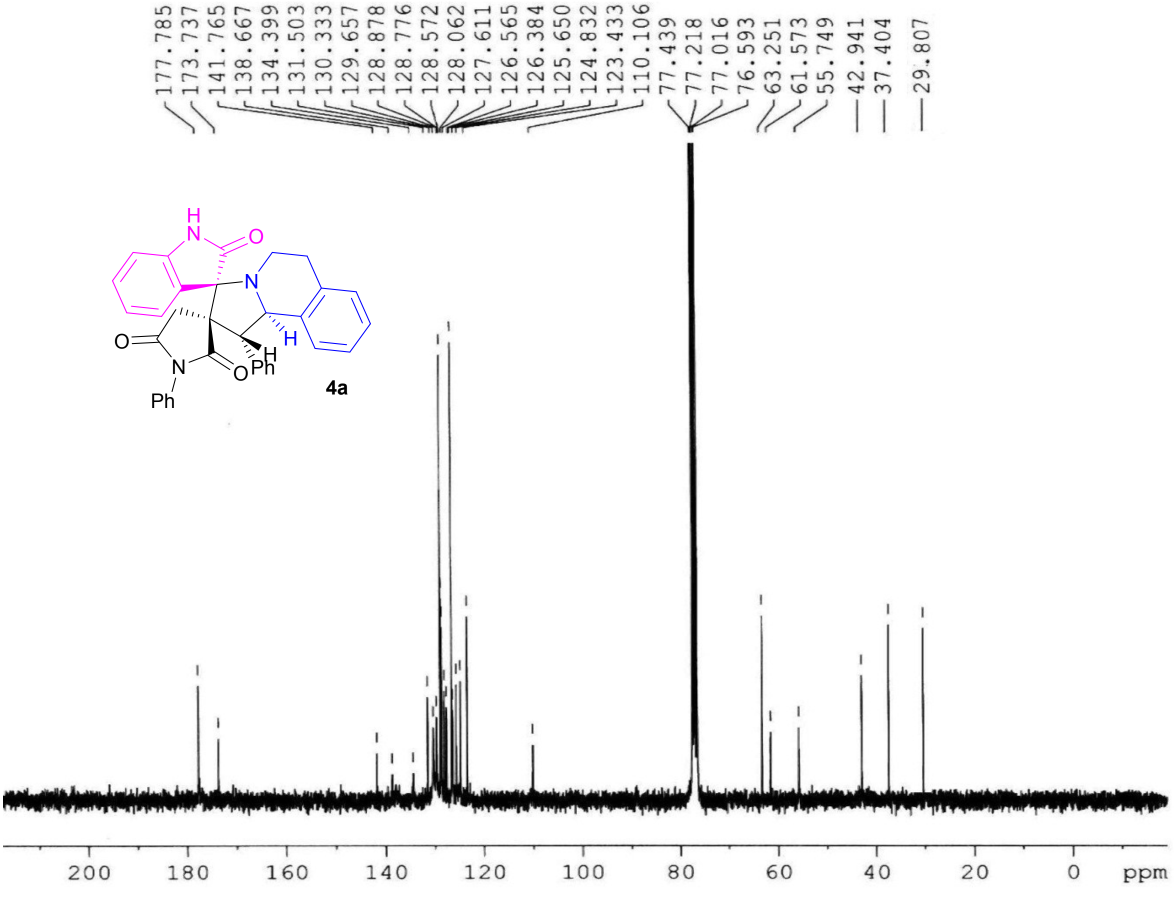

Fig. S2.^{13}C NMR spectrum of **4a** in $CDCl_3$

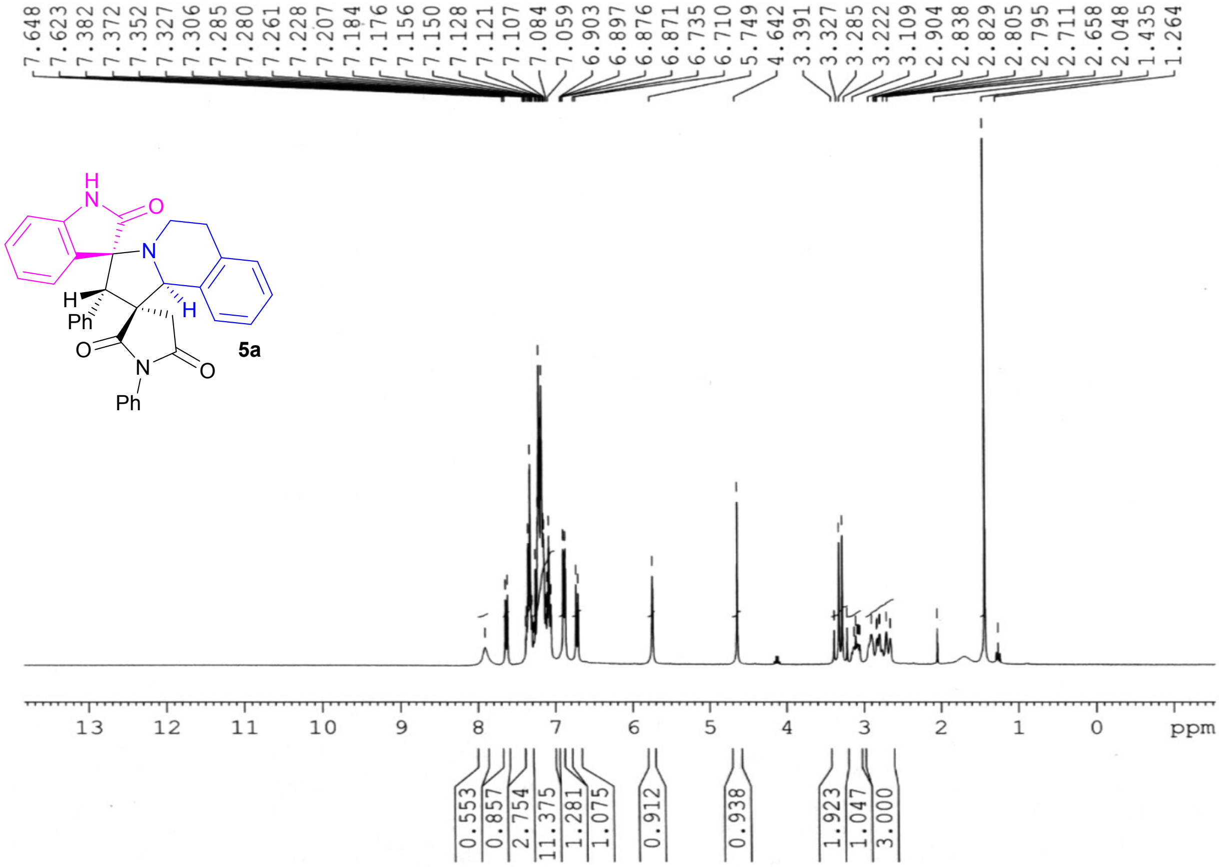

Fig. S3. ^{1}H NMR spectrum of **5a** in $CDCl_3$

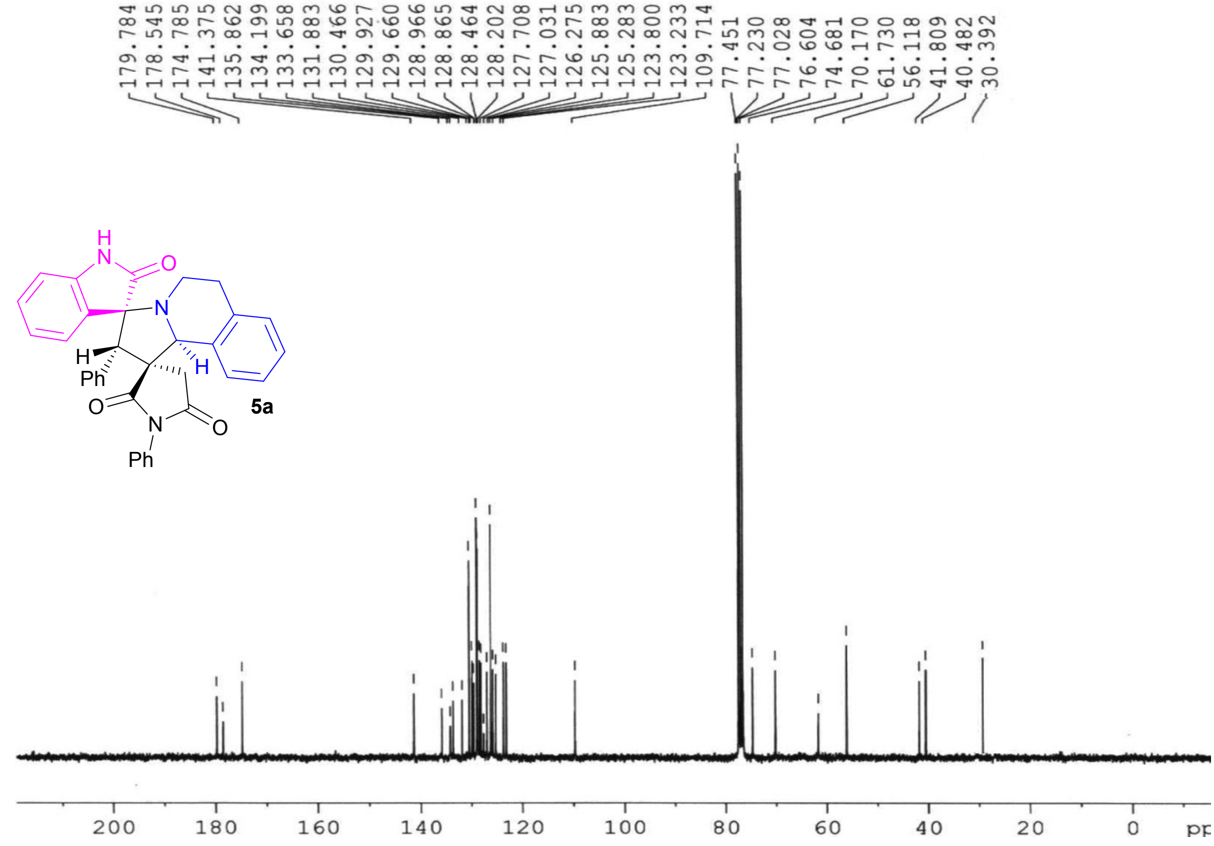

Fig. S4. ^{13}C NMR spectrum of **5a** in $CDCl_3$

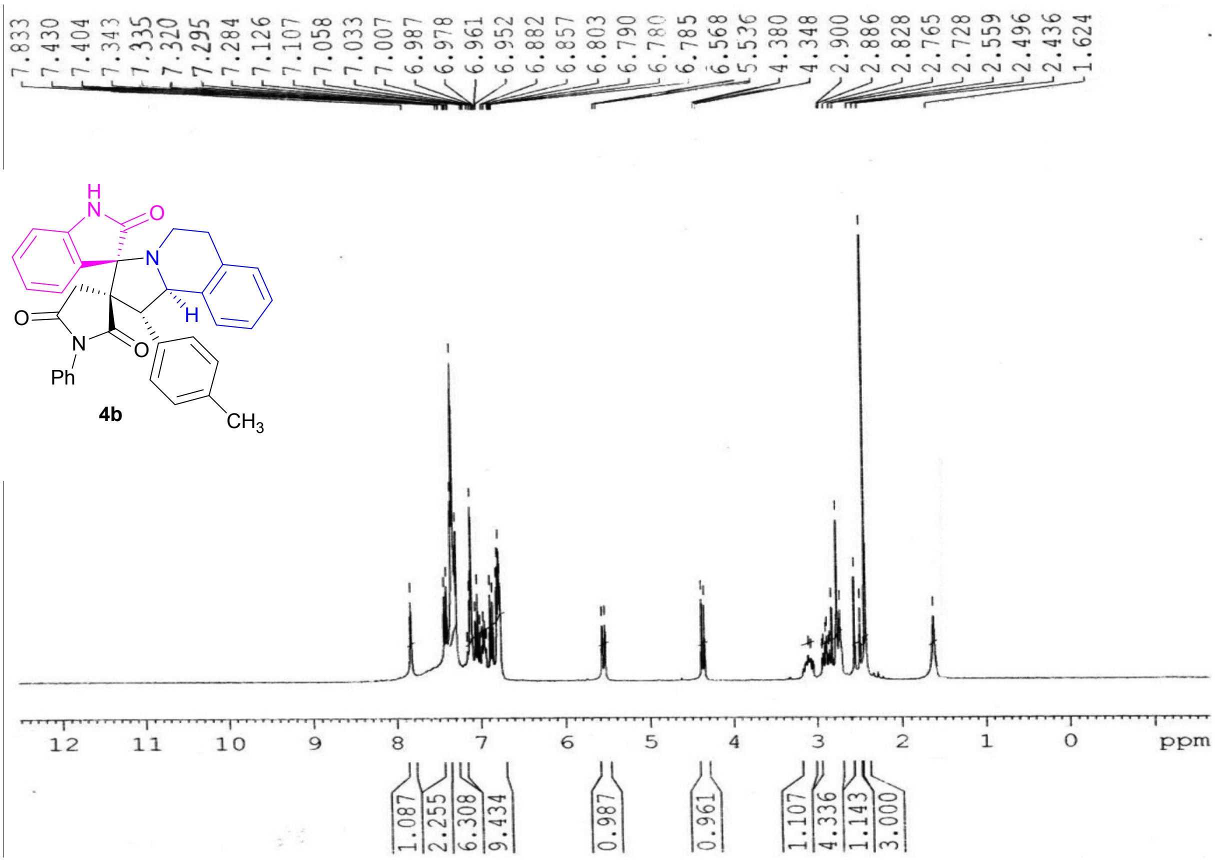

Fig. S5. ^{1}H NMR spectrum of **4b** in $CDCl_3$

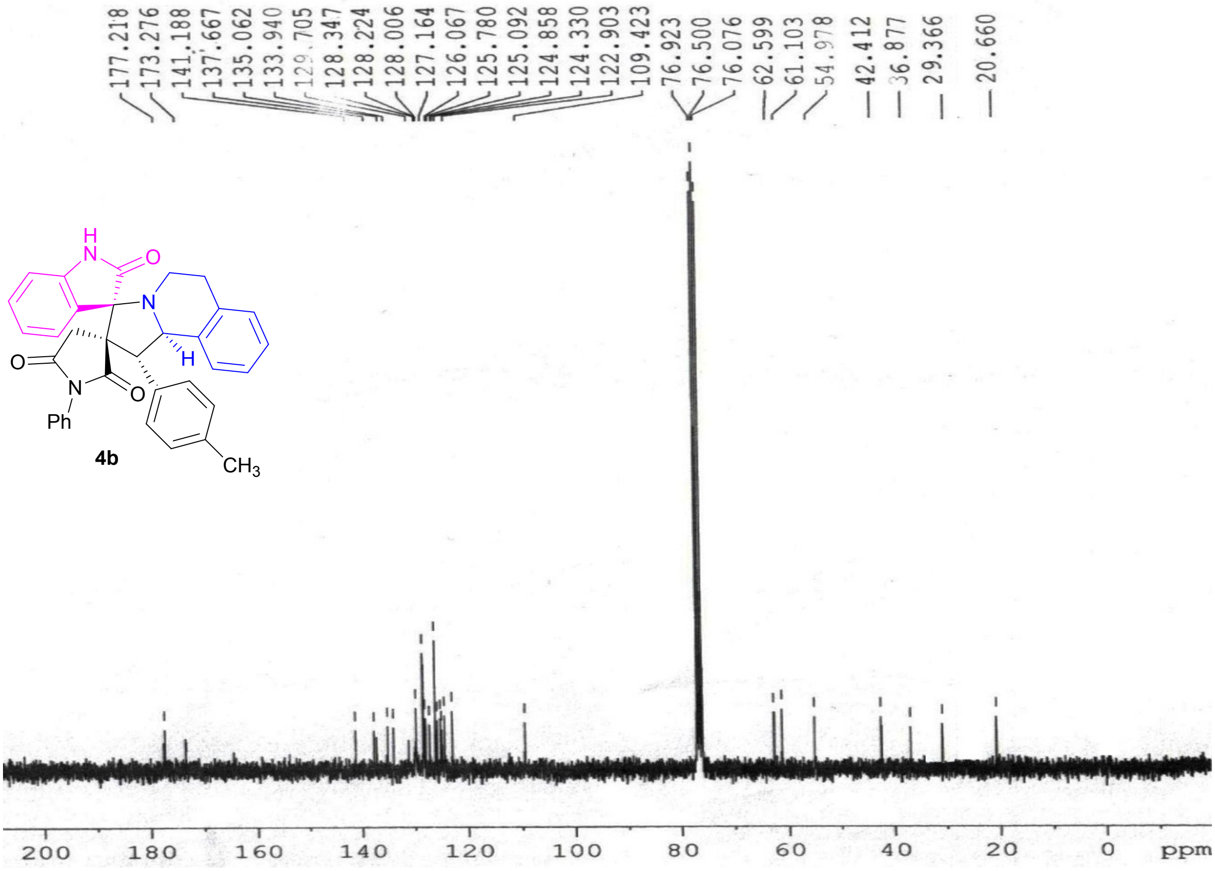

Fig. S6. ^{13}C NMR spectrum of **4b** in $CDCl_3$

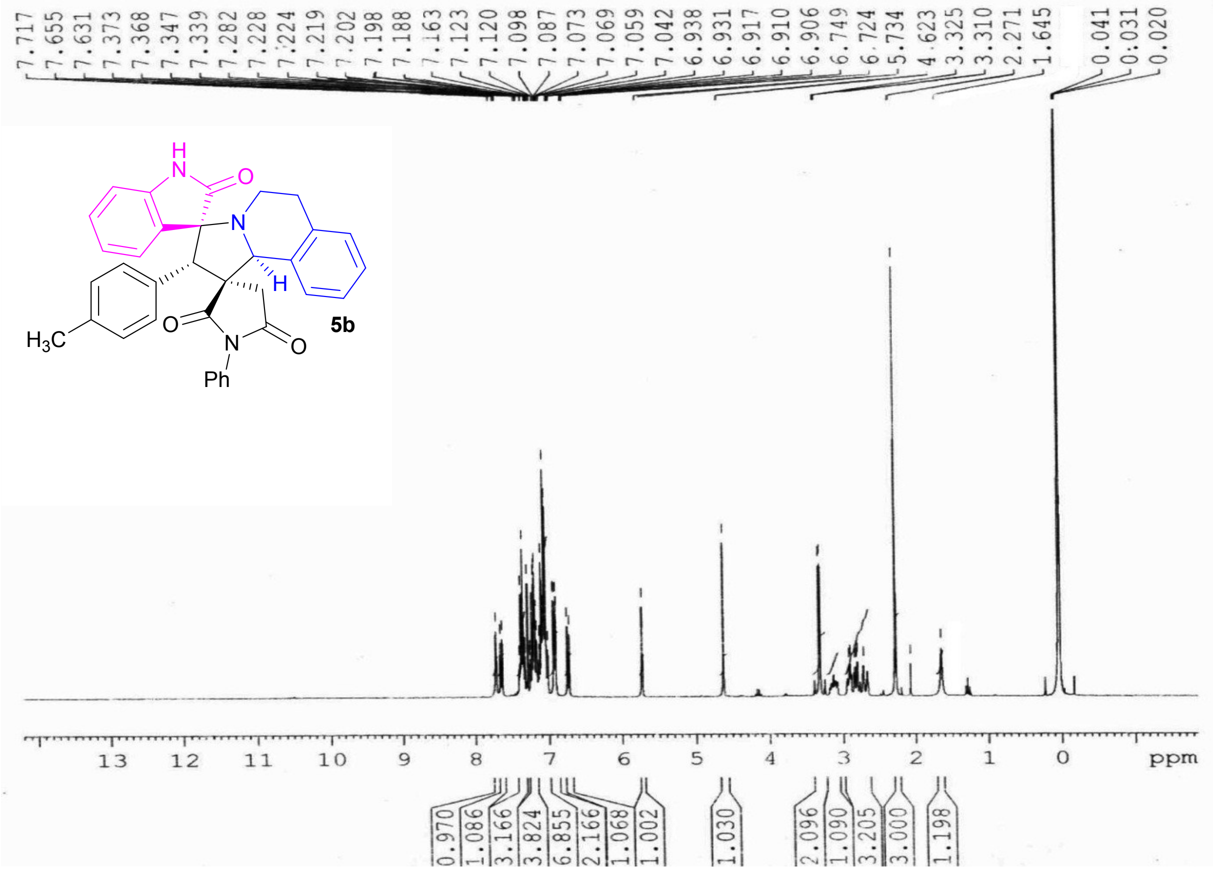

Fig. S7. ^{1}H NMR spectrum of **5b** in $CDCl_3$

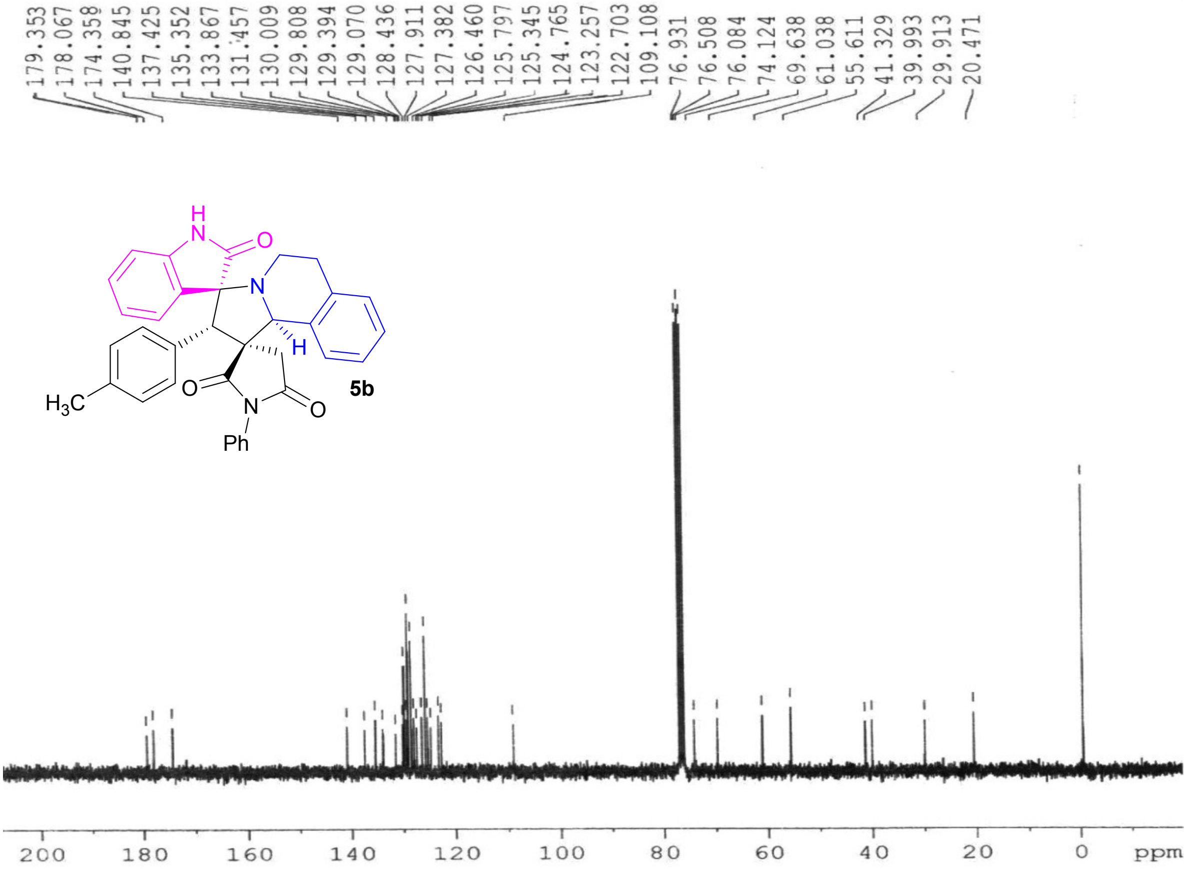

Fig. S8. ^{13}C NMR spectrum of **5b** in $CDCl_3$

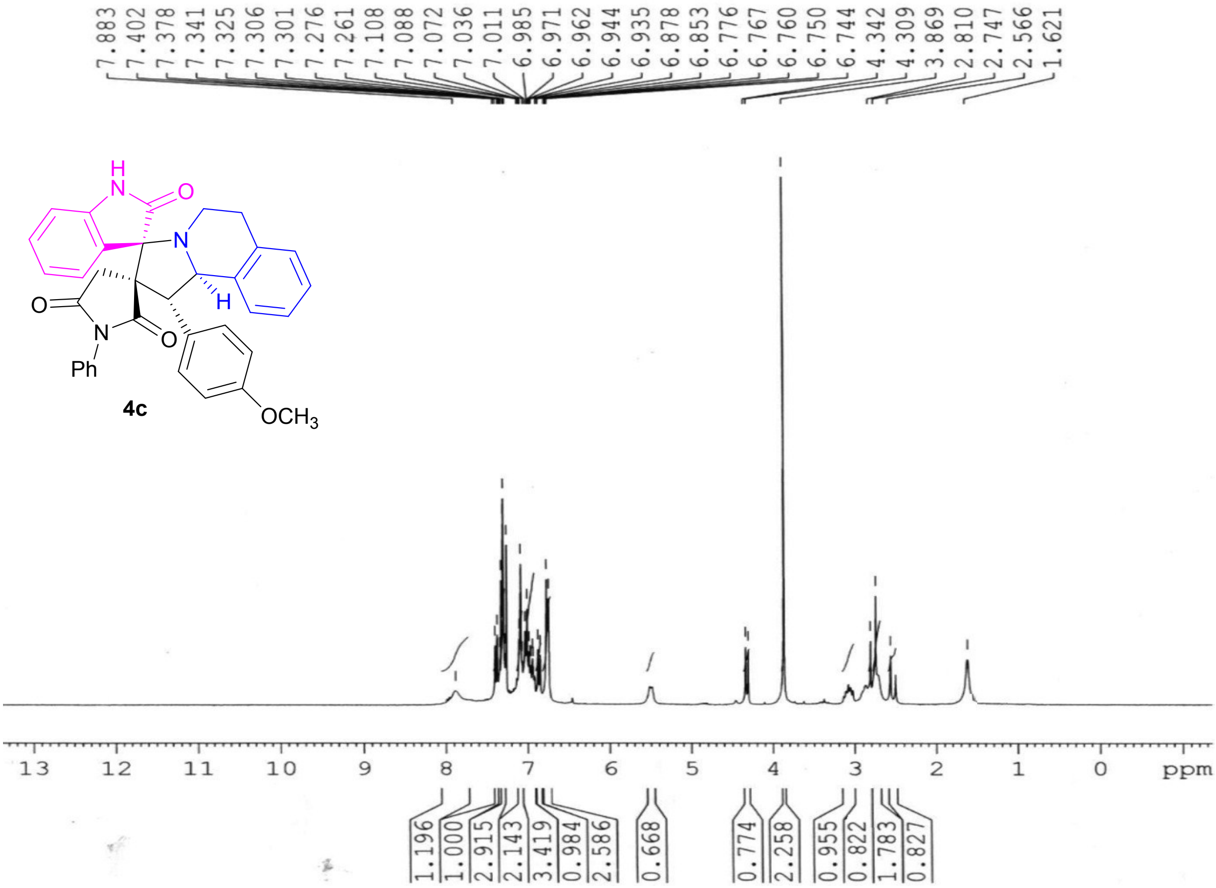

Fig. S9. ^{1}H NMR spectrum of **4c** in $CDCl_3$

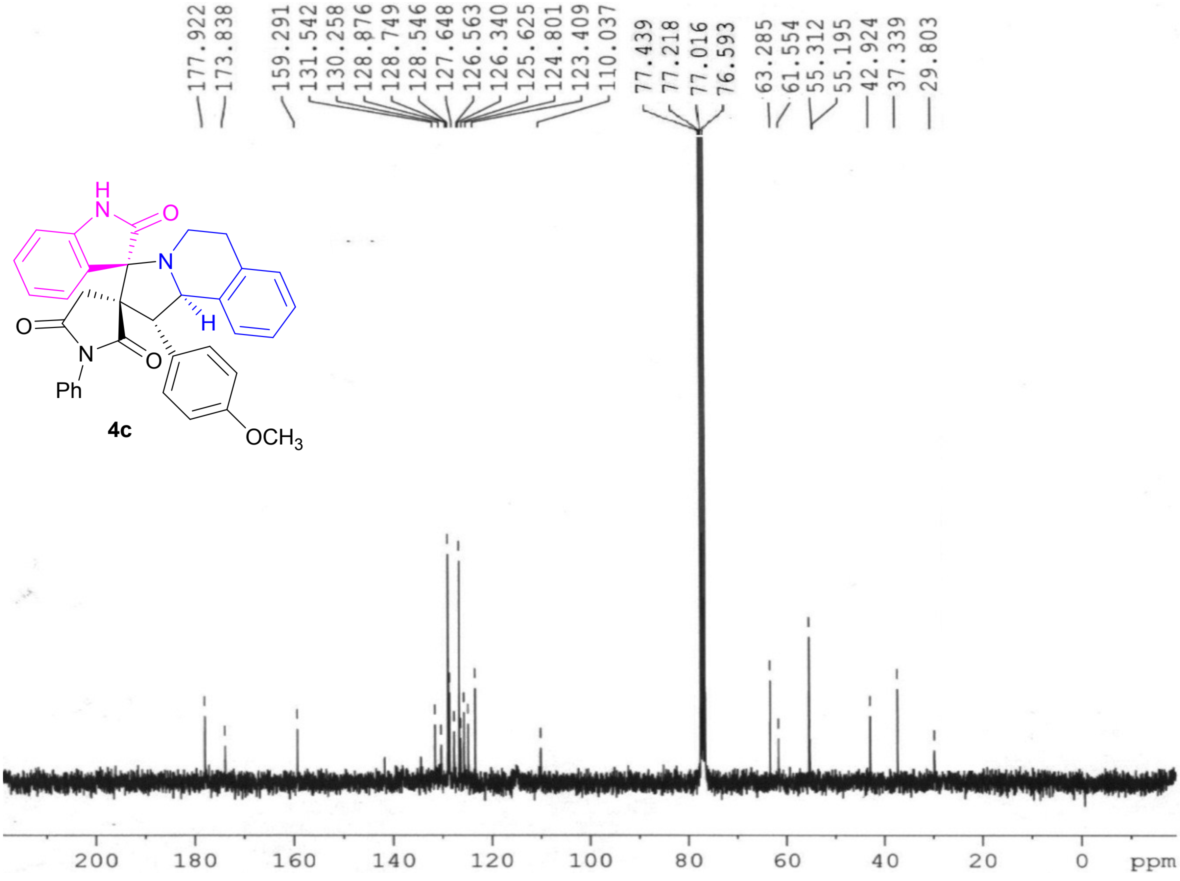

Fig. S10. ^{13}C NMR spectrum of **4c** in $CDCl_3$

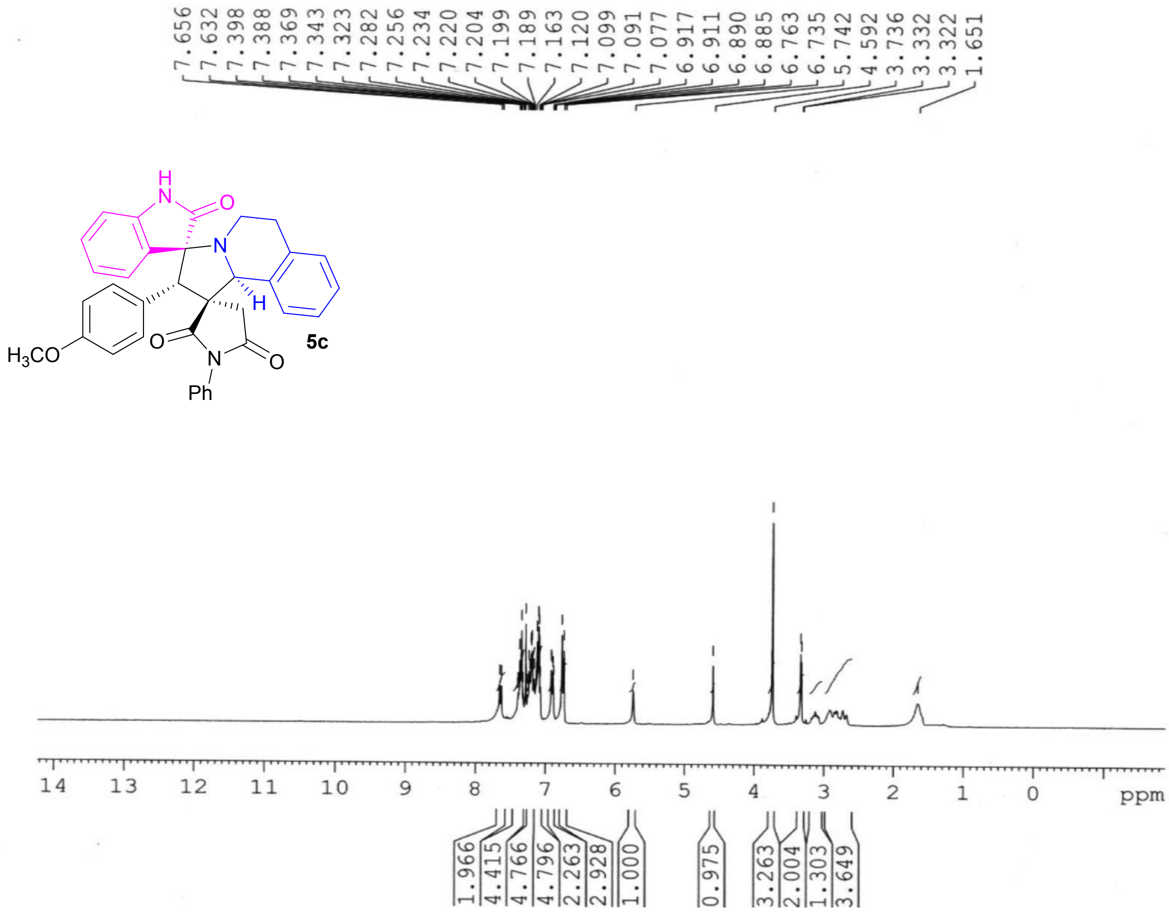

Fig. S11. ^{1}H NMR spectrum of **5c** in $CDCl_3$

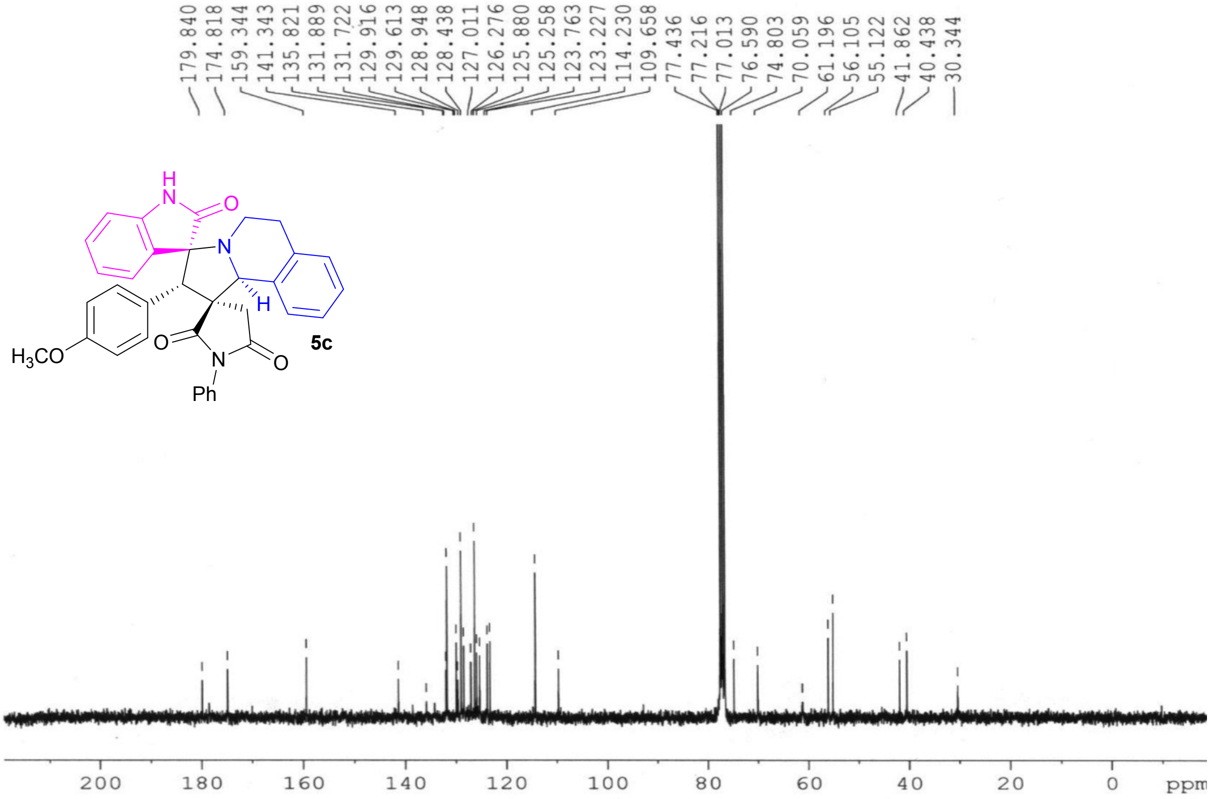

Fig. S12. ^{13}C NMR spectrum of **5c** in $CDCl_3$

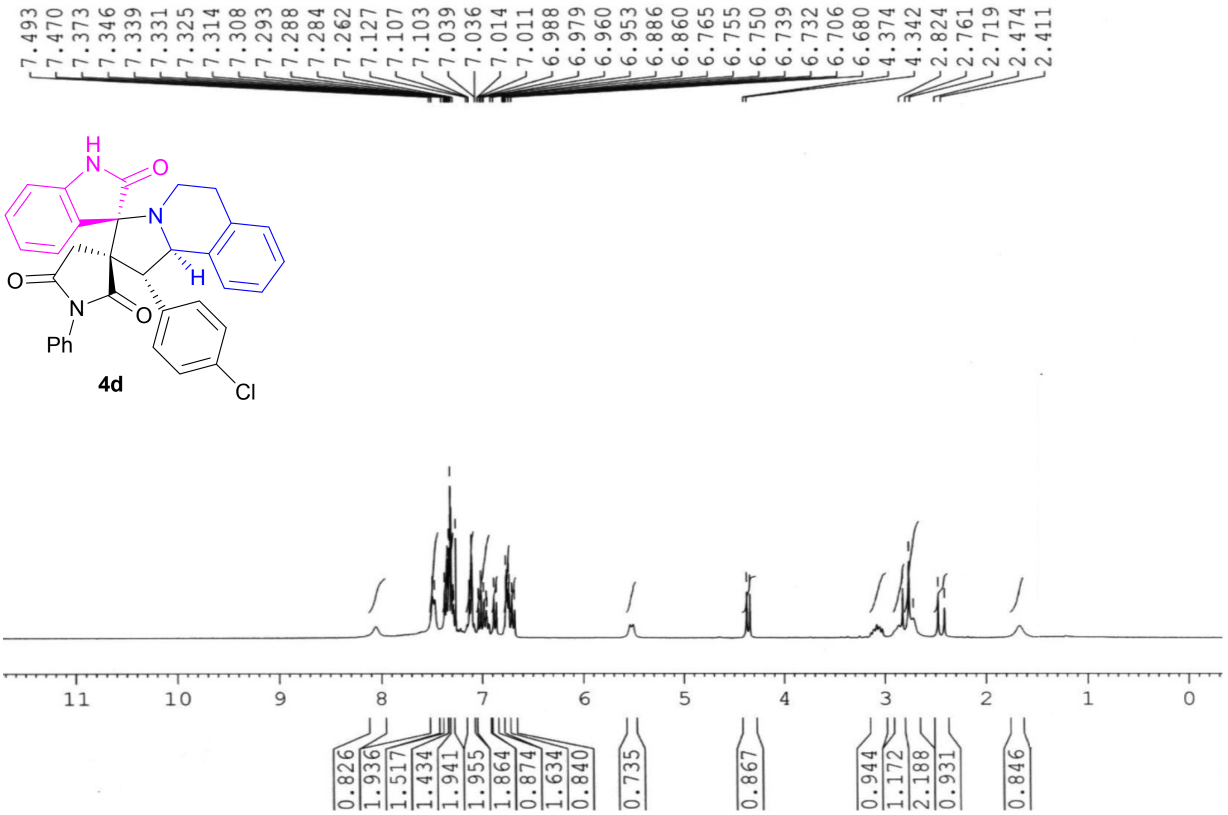

Fig. S13. ^{1}H NMR spectrum of **4d** in $CDCl_3$

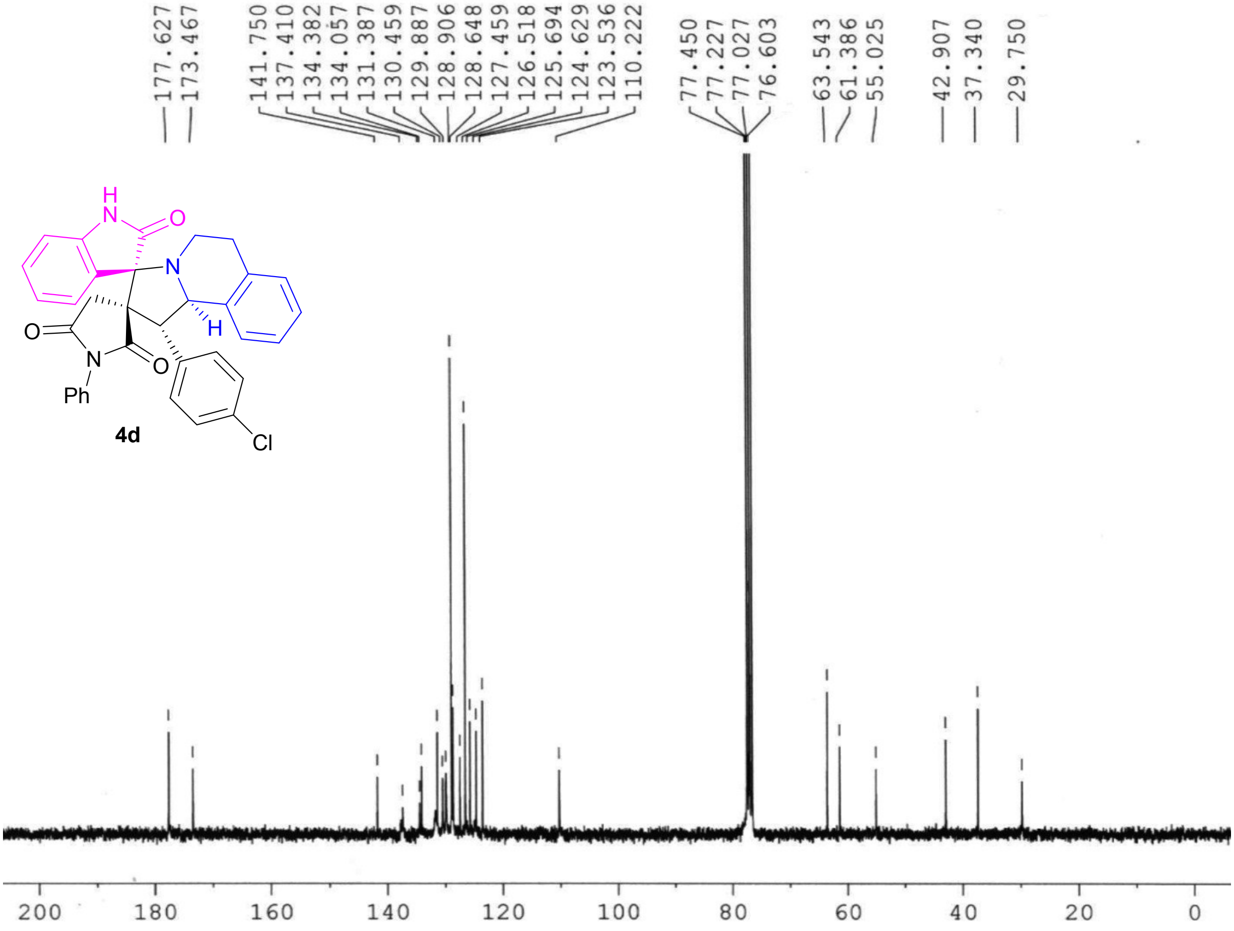

Fig. S14. ^{13}C NMR spectrum of **4d** in $CDCl_3$

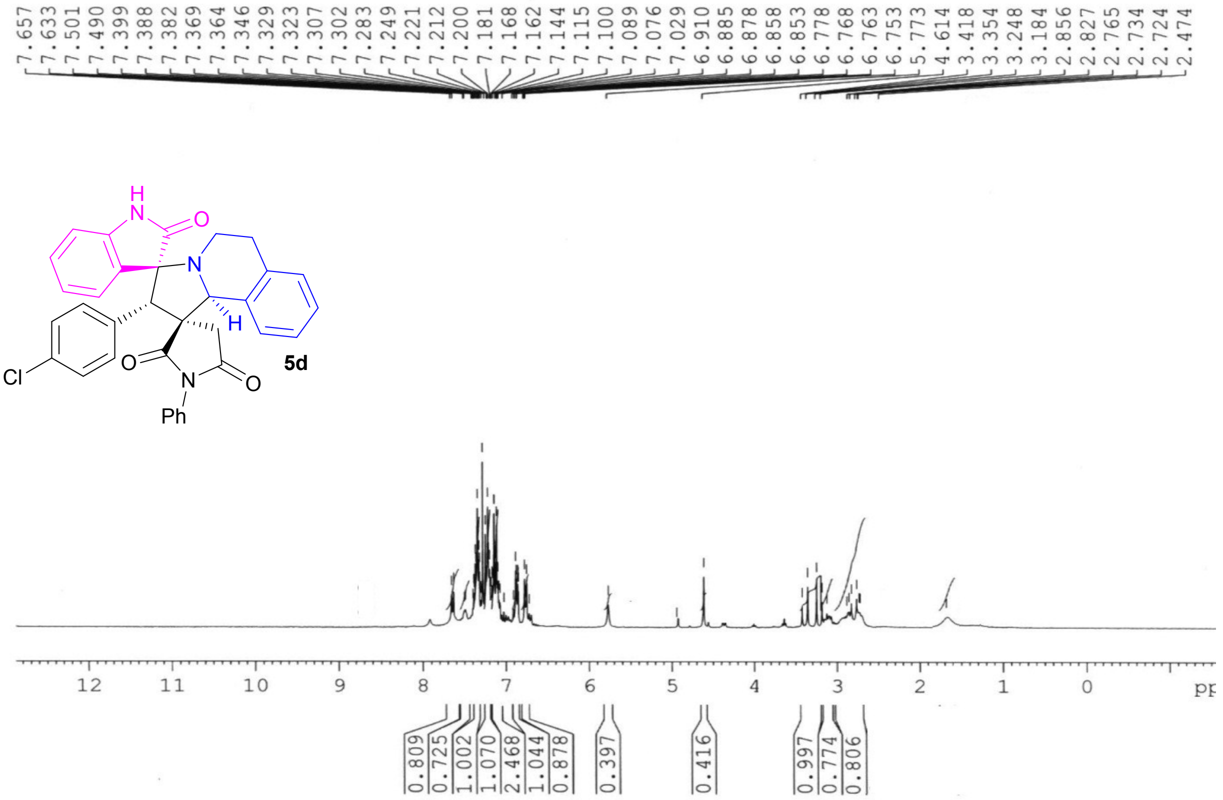

Fig. S15. ^{1}H NMR spectrum of **5d** in $CDCl_3$

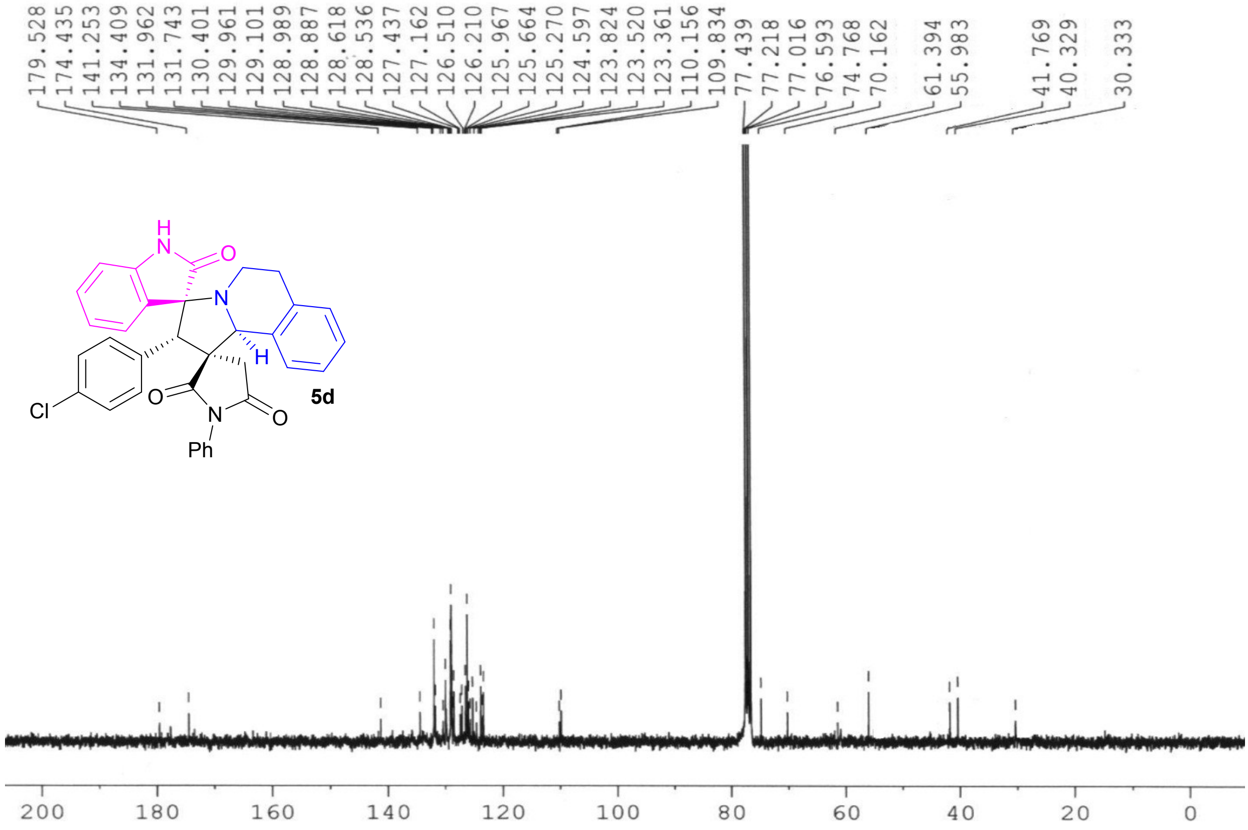

Fig. S16. ^{13}C NMR spectrum of **5d** in $CDCl_3$

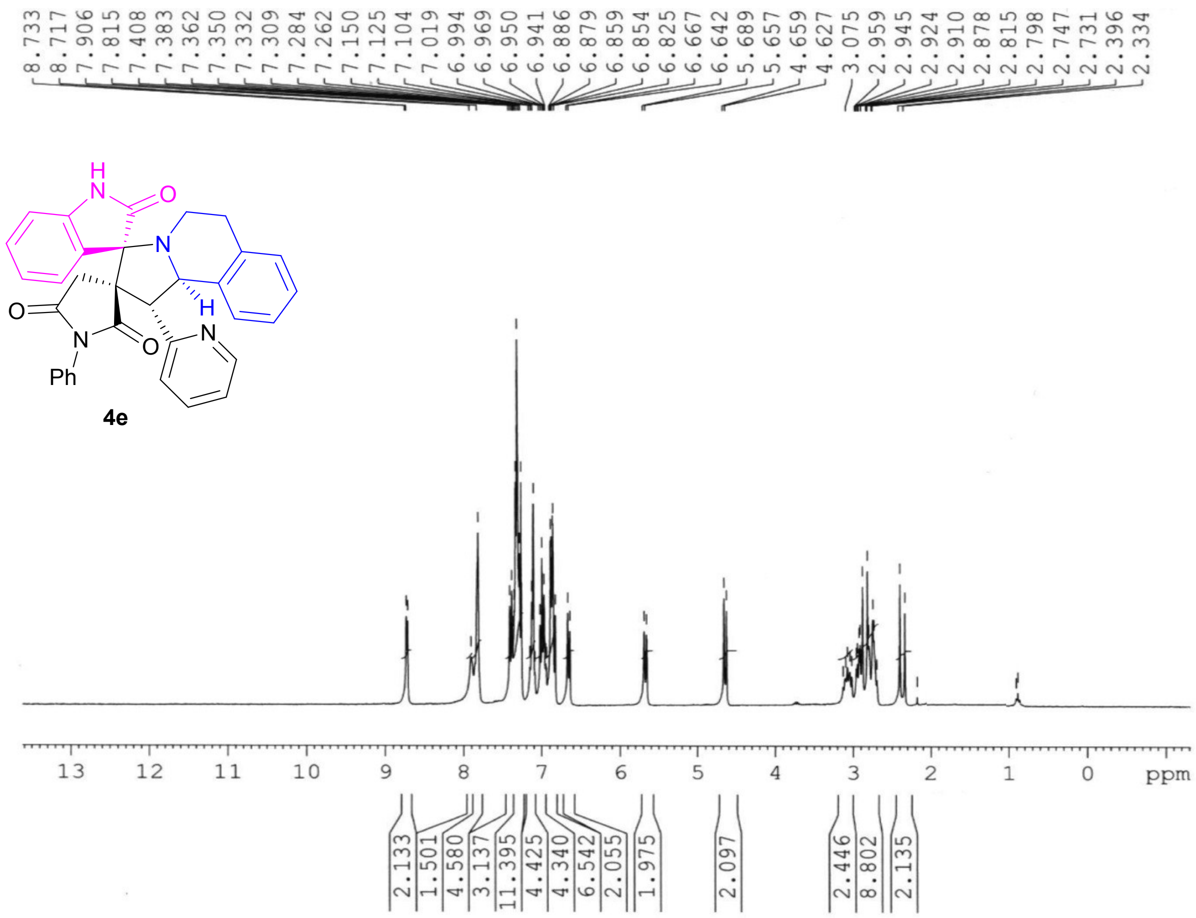

Fig. S17. ^{1}H NMR spectrum of **4e** in $CDCl_3$

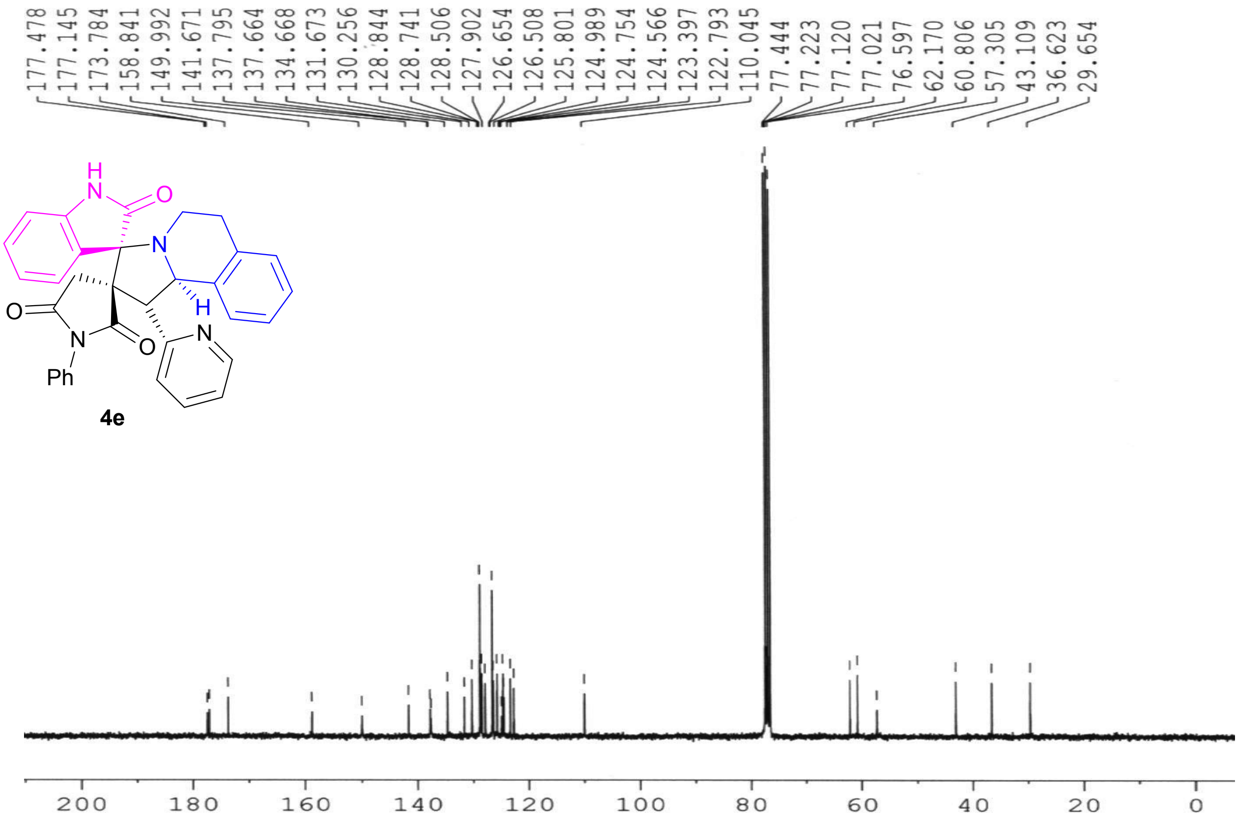

Fig. S18. ^{13}C NMR spectrum of **4e** in $CDCl_3$

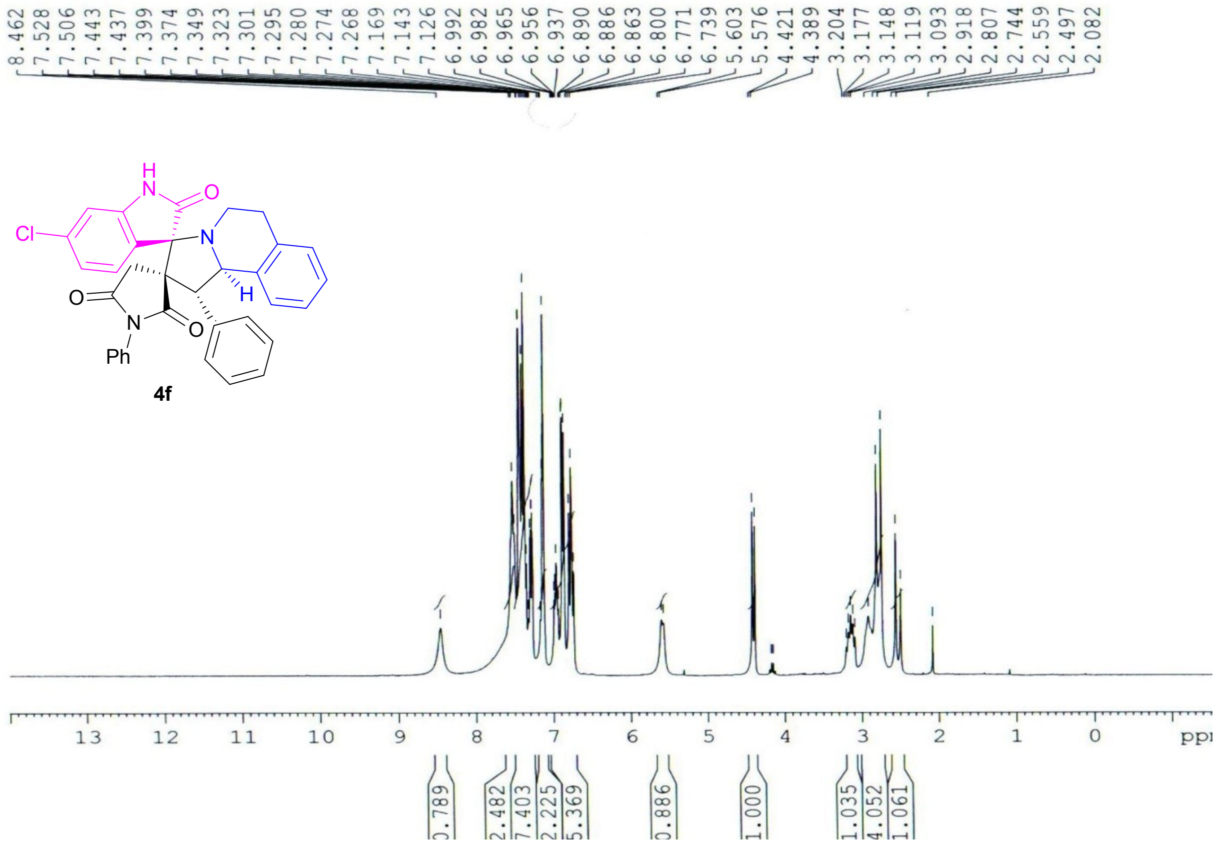

Fig. S19. ^{1}H NMR spectrum of **4f** in $CDCl_3$

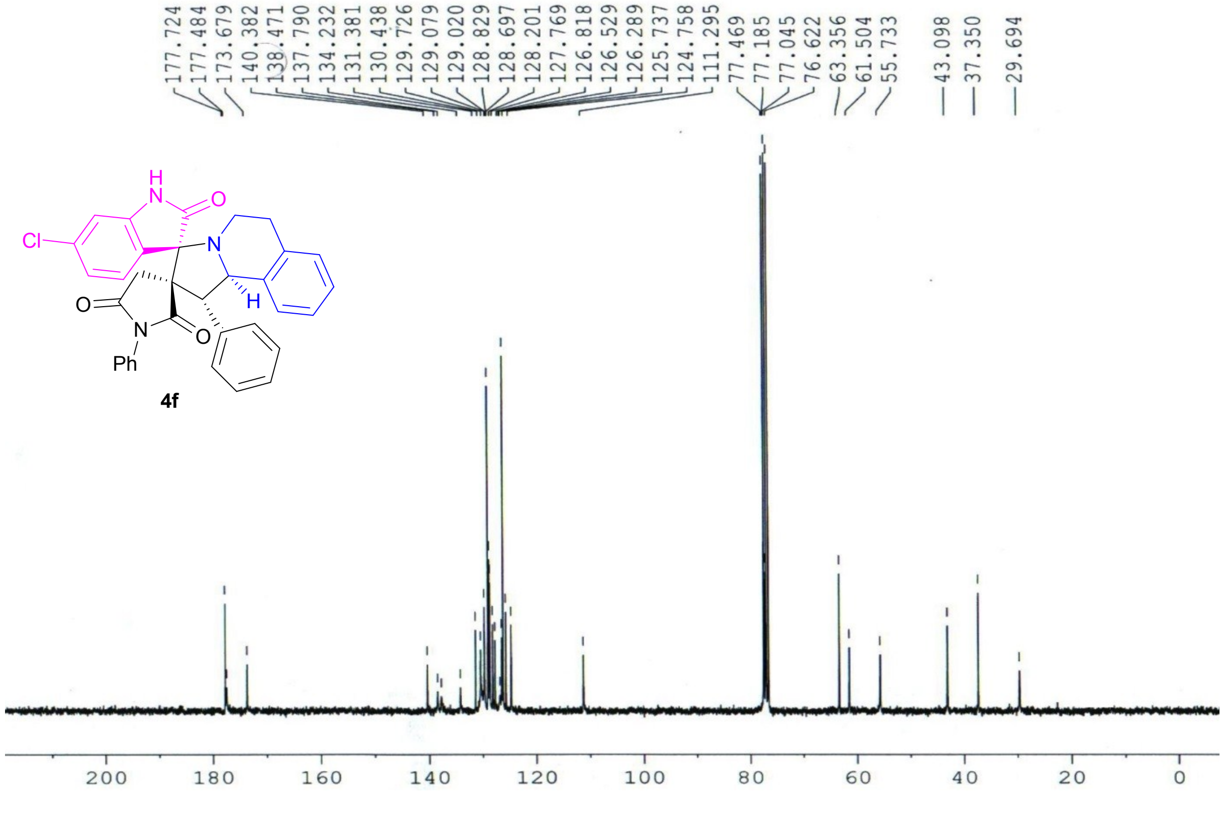

Fig. S20. ^{13}C NMR spectrum of**4f** in $CDCl_3$

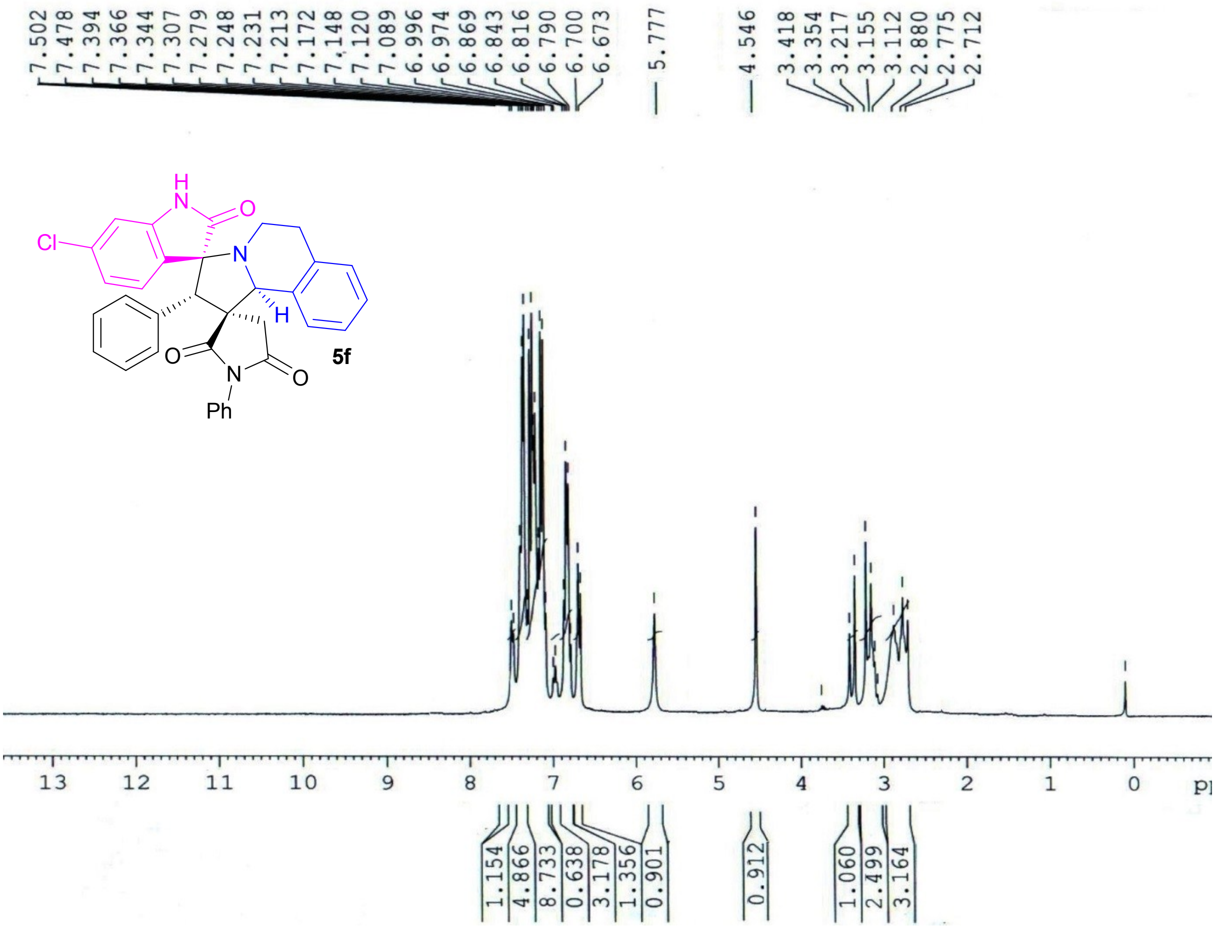

Fig. S21. ^{1}H NMR spectrum of **5f** in $CDCl_3$

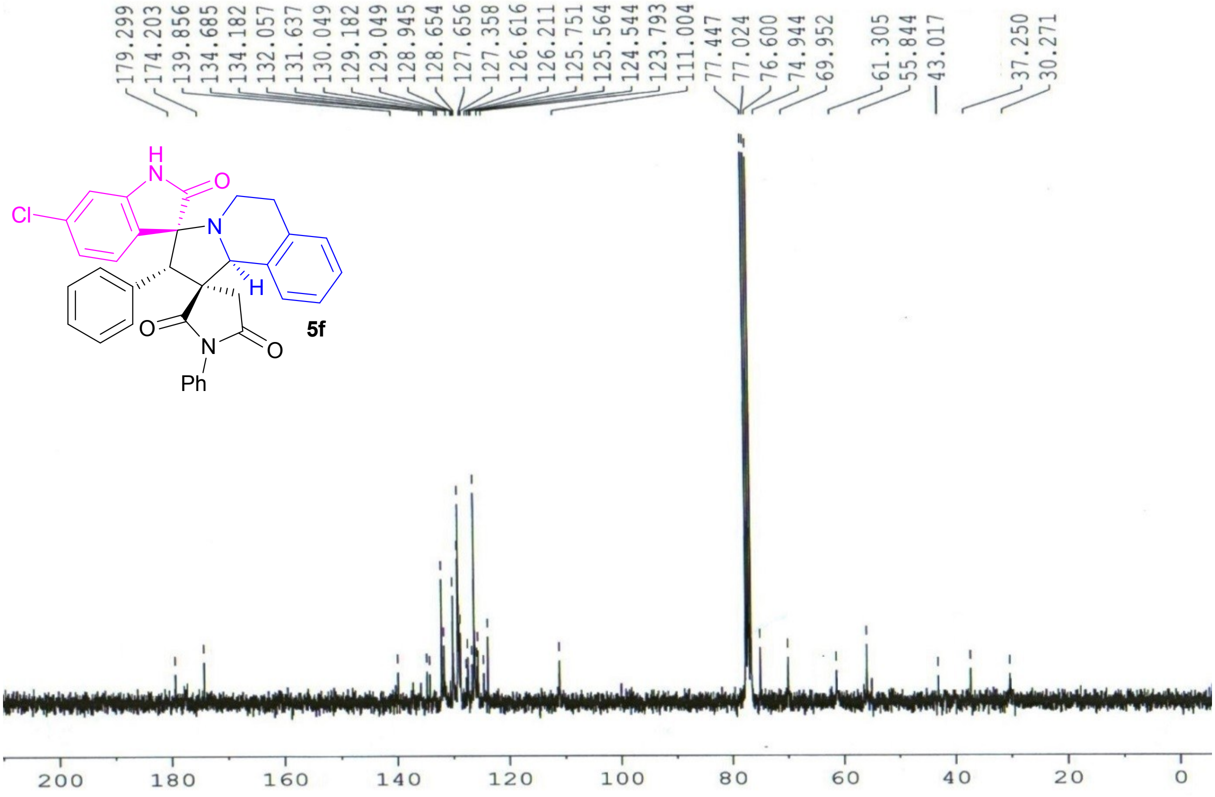

Fig. S22. ^{13}C NMR spectrum of **5f** in $CDCl_3$

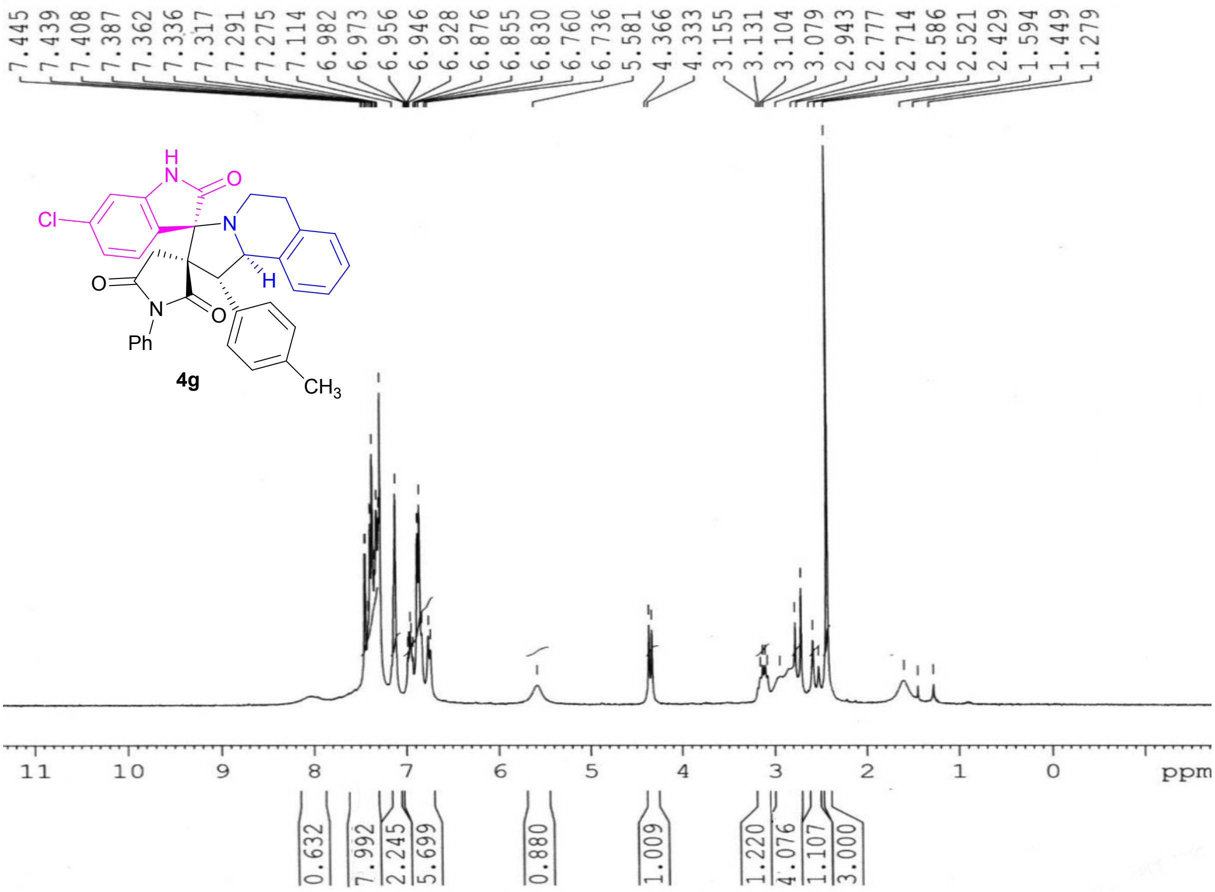

Fig. S23. ^{1}H NMR spectrum of **4g** in $CDCl_3$

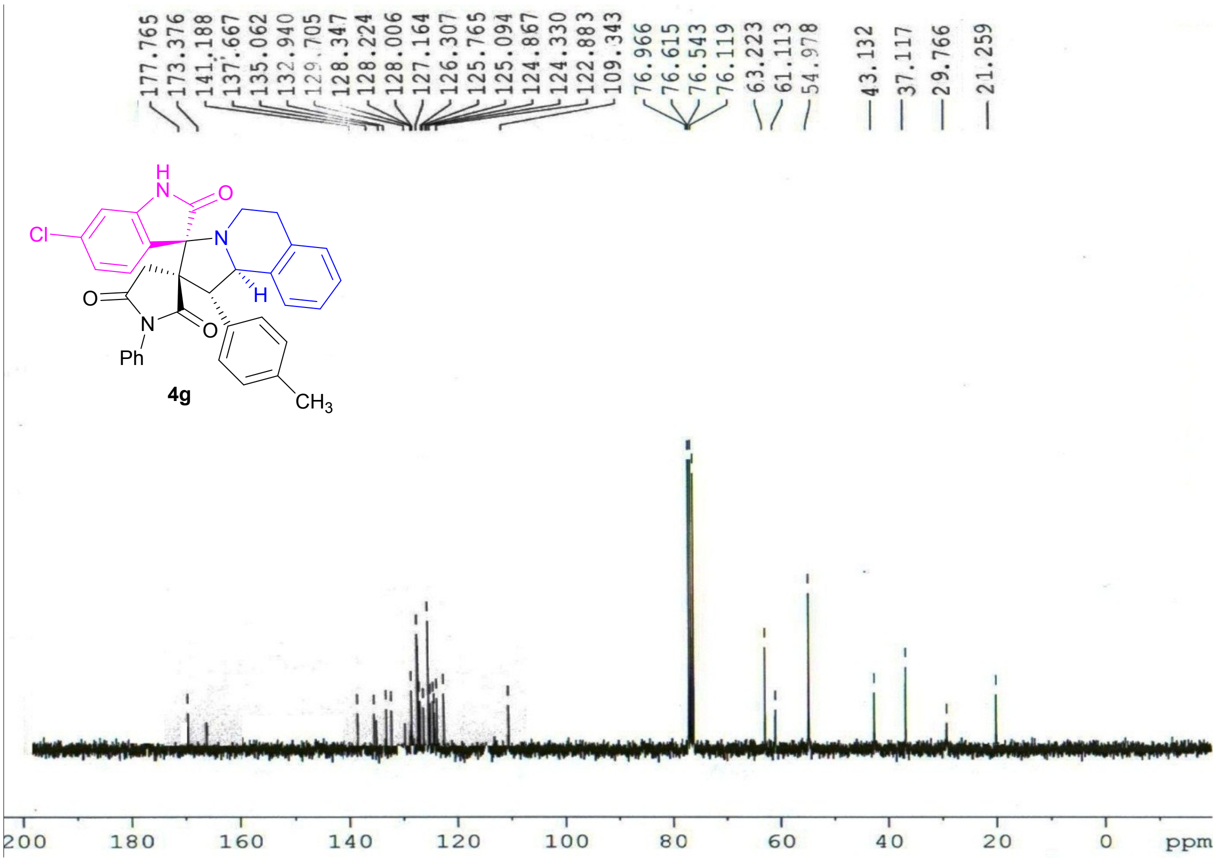

Fig. S24. ^{13}C NMR spectrum of **4g** in $CDCl_3$

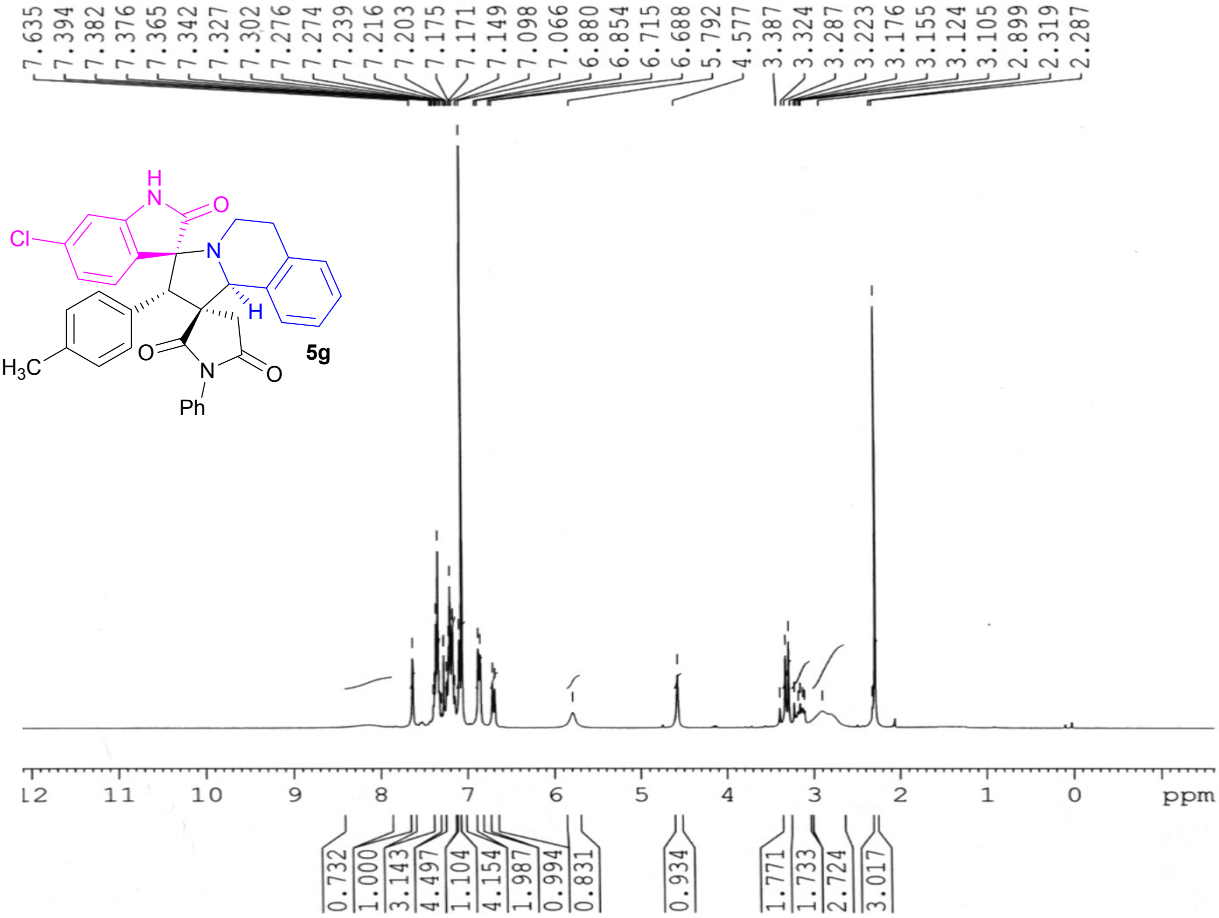

Fig. S25. ^{1}H NMR spectrum of **5g** in $CDCl_3$

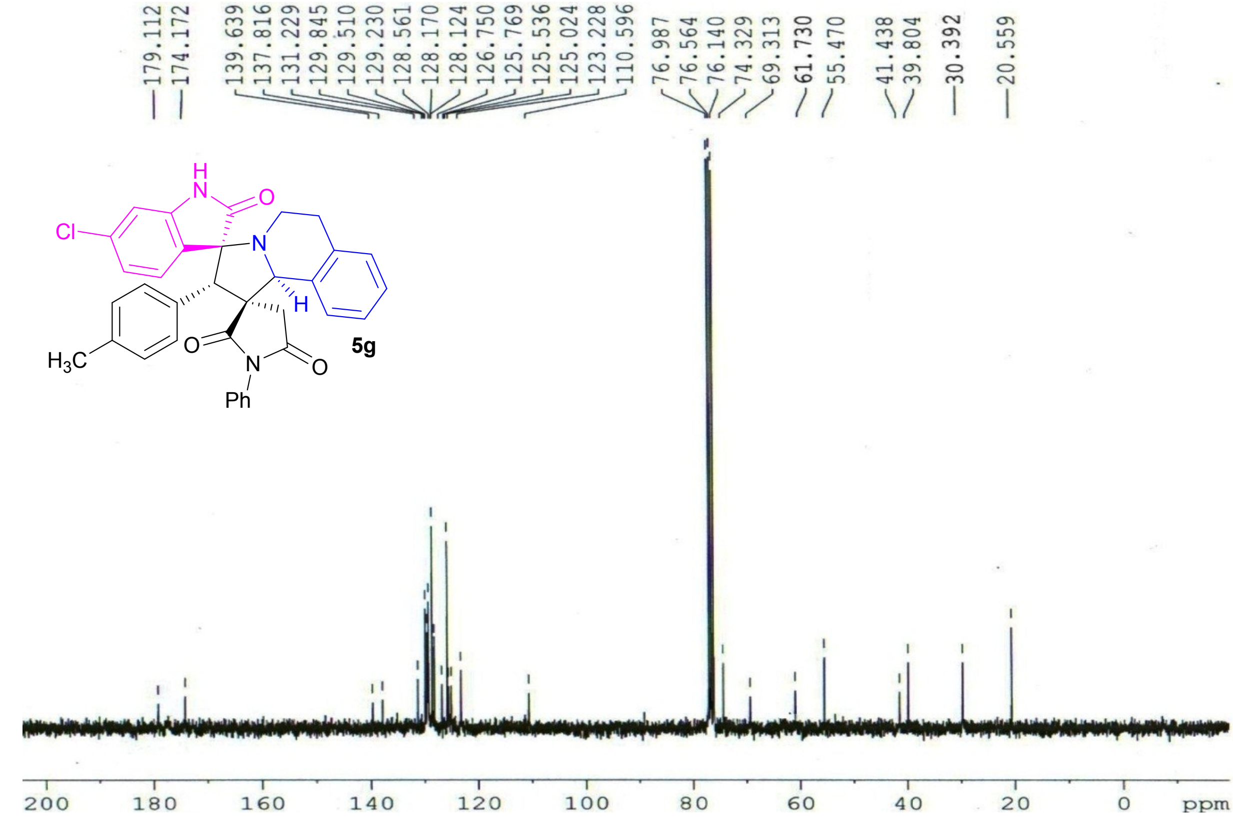

Fig. S26. ^{13}C NMR spectrum of **5g** in $CDCl_3$

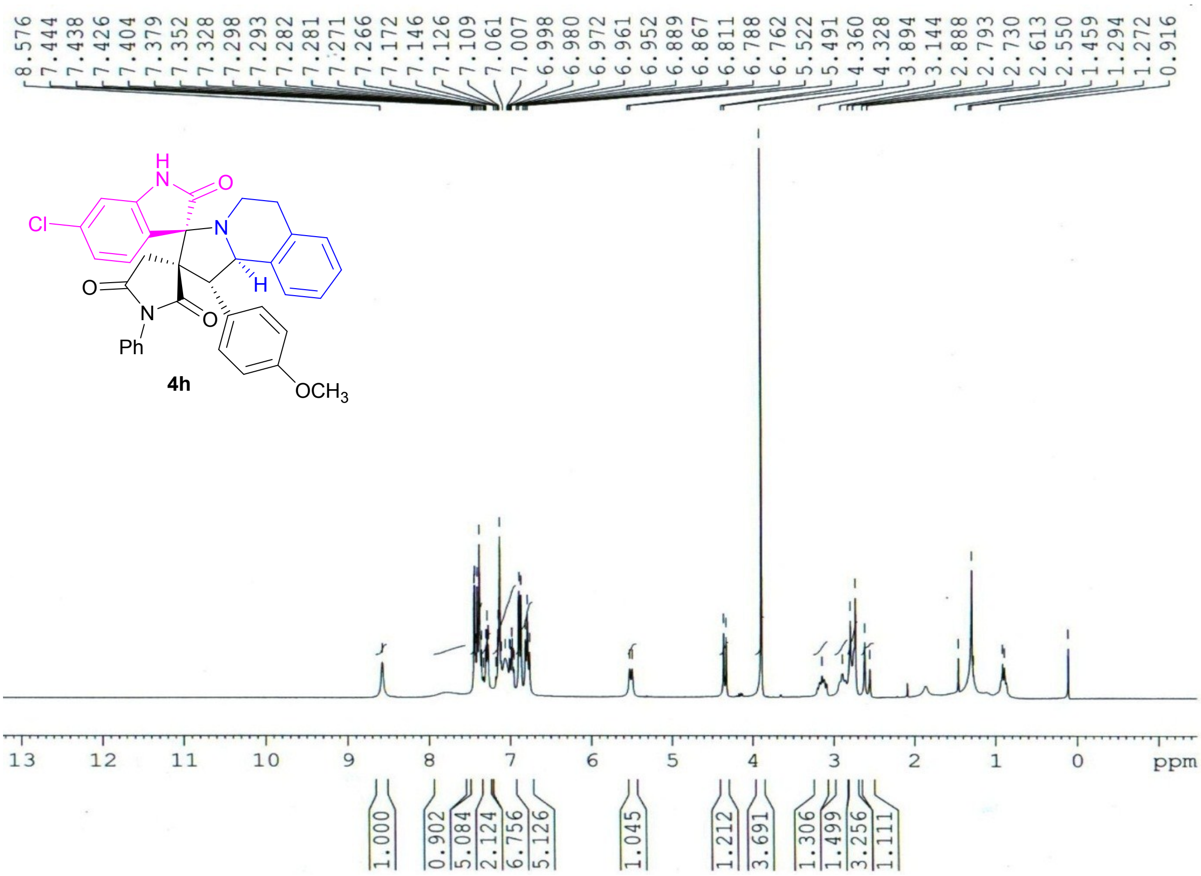

Fig. S27. ^{1}H NMR spectrum of **4h** in $CDCl_3$

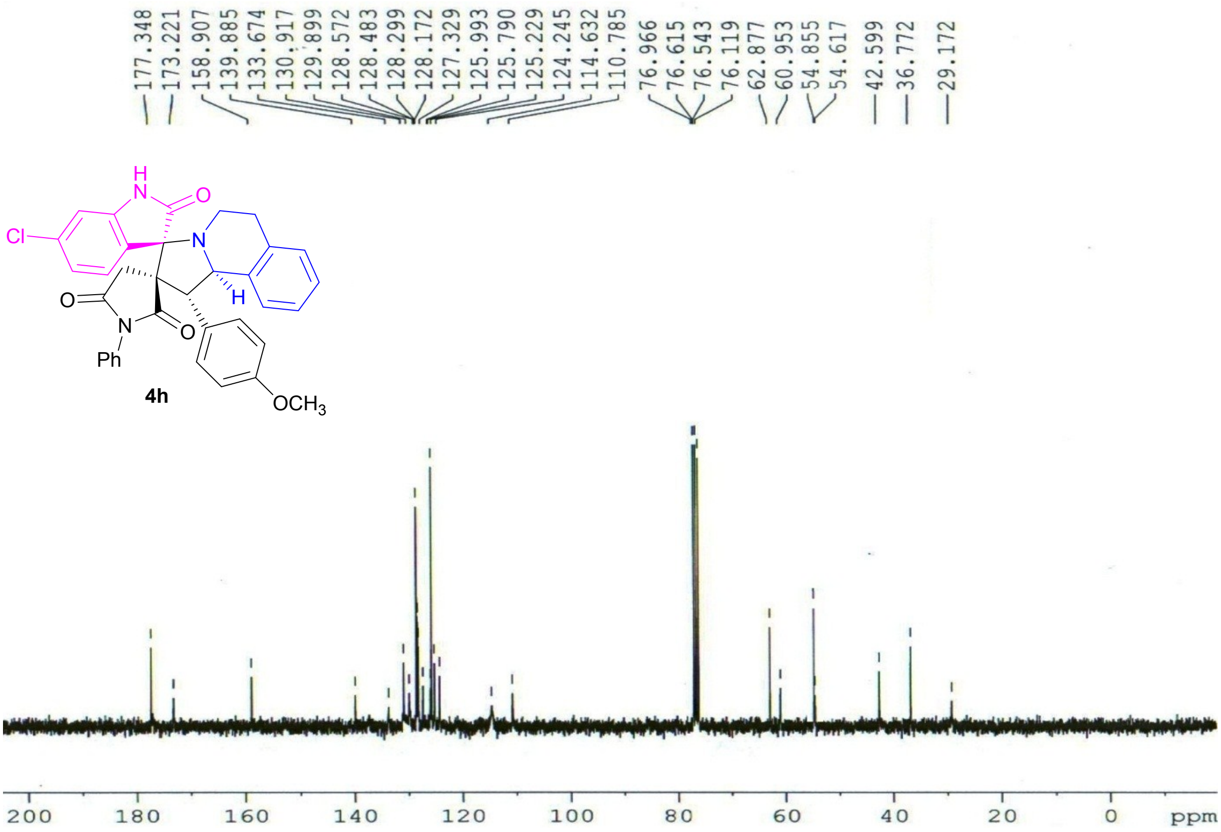

Fig. S28. ^{13}C NMR spectrum of **4h** in $CDCl_3$

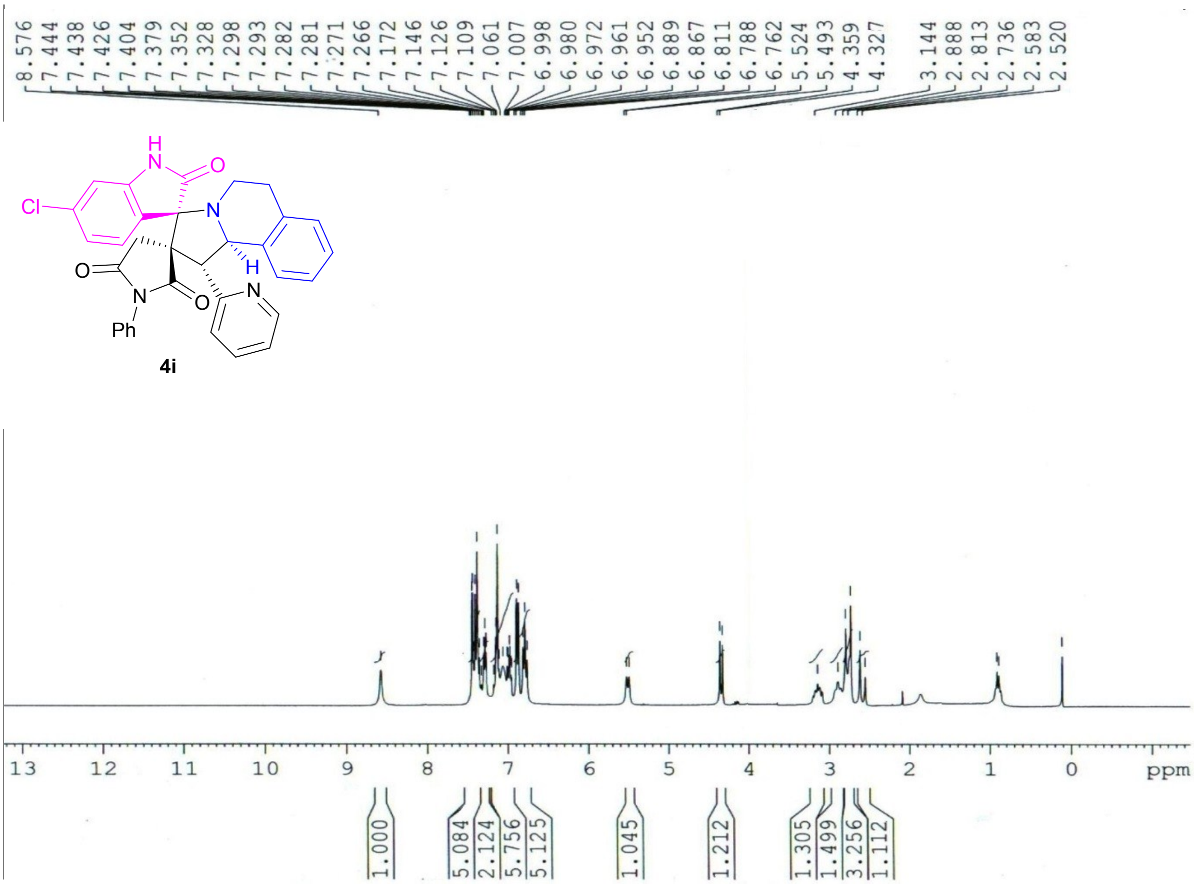

Fig. S29. ^{1}H NMR spectrum of **4i** in $CDCl_3$

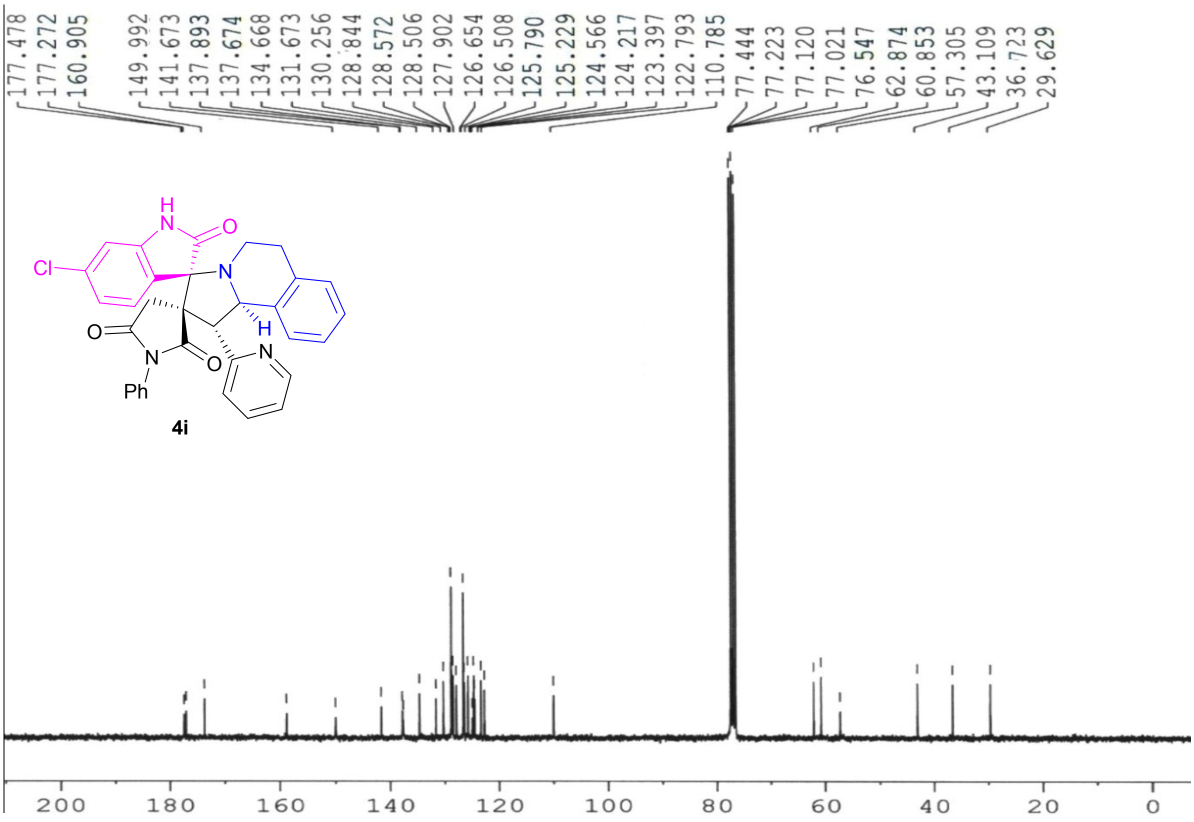

Fig. S30. ^{13}C NMR spectrum of **4i** in $CDCl_3$

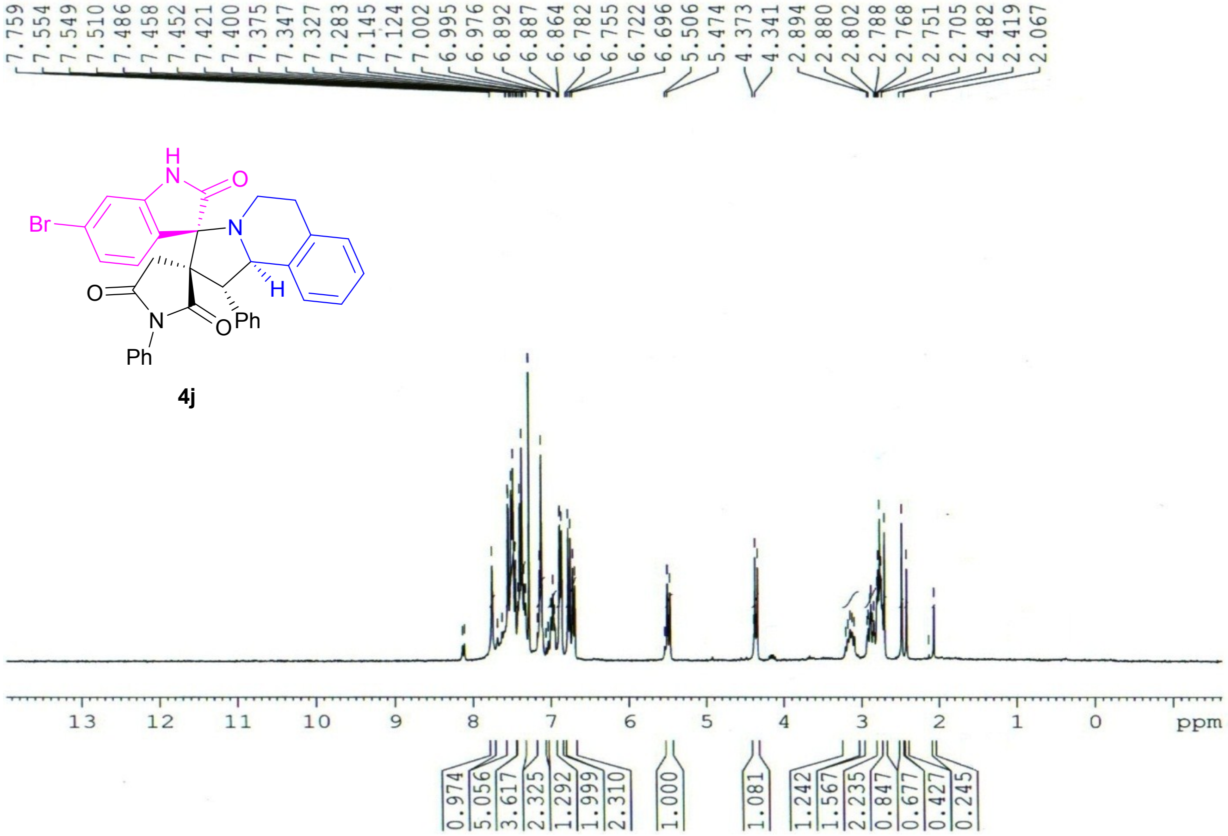

Fig. S31. ^{1}H NMR spectrum of **4j** in $CDCl_3$

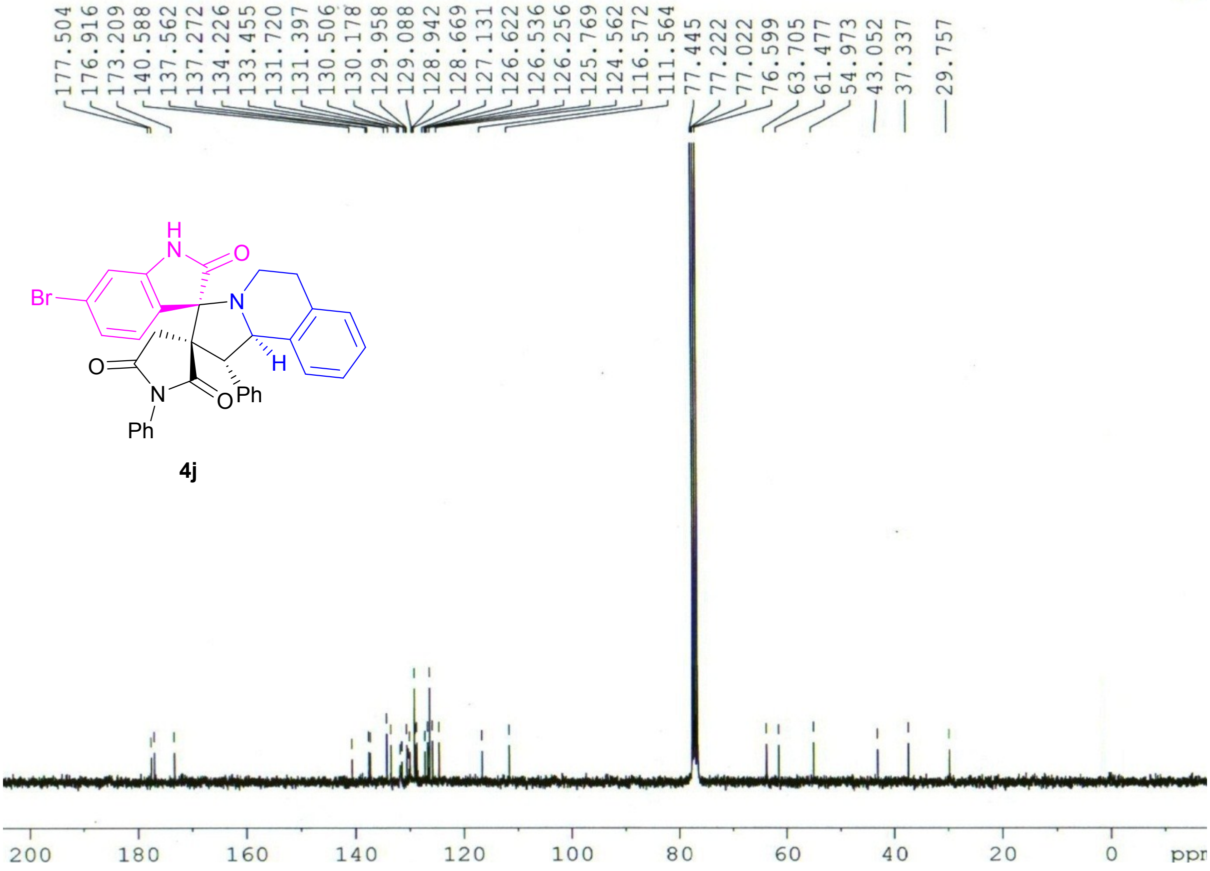

Fig. S32. ^{13}C NMR spectrum of **4j** in $CDCl_3$

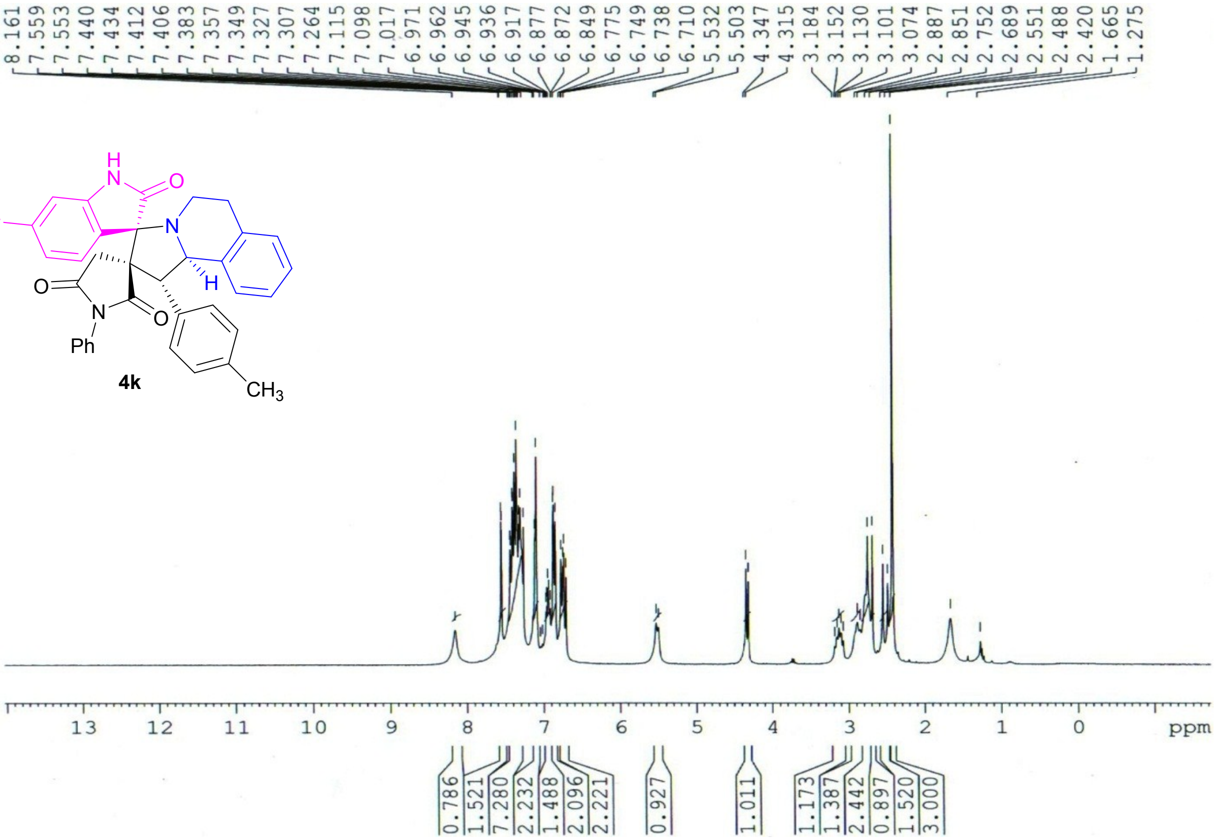

Fig. S33. ^{1}H NMR spectrum of **4k** in $CDCl_3$

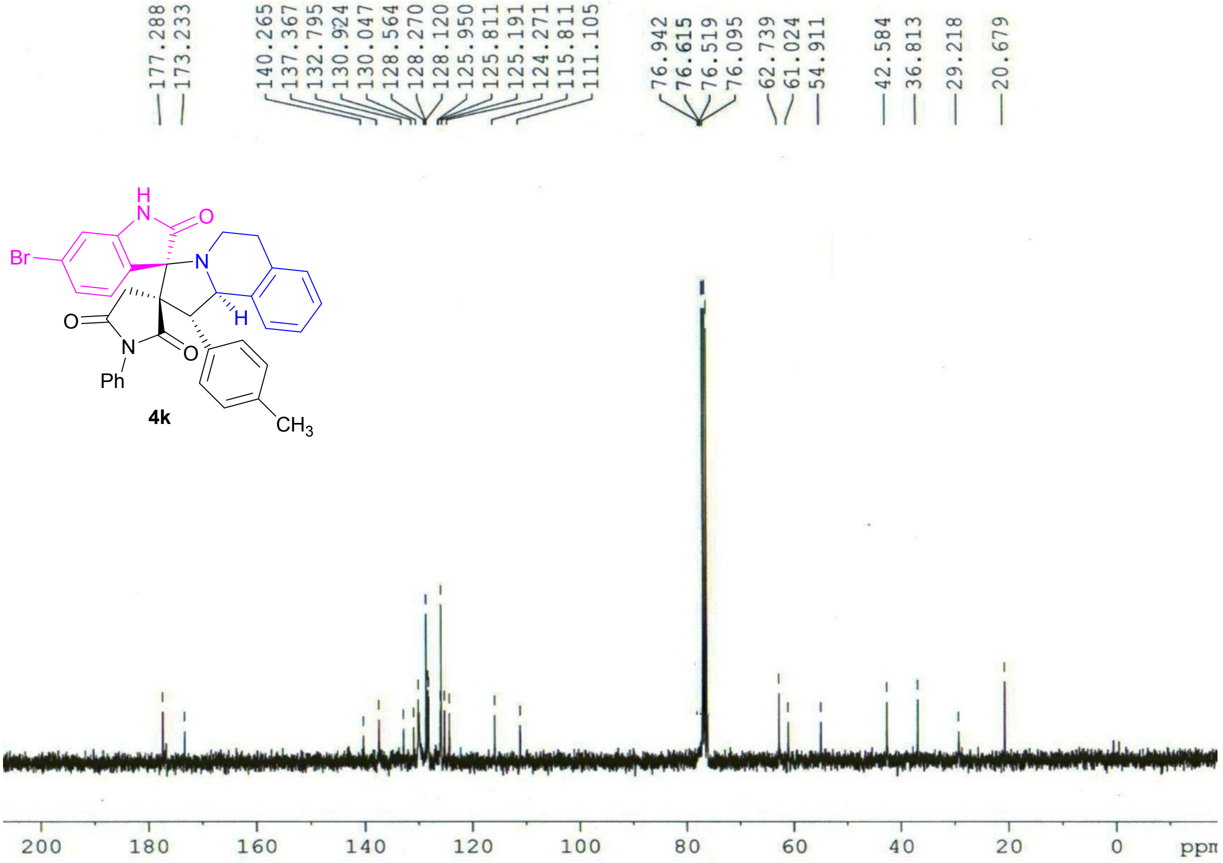

Fig. S34. ^{13}C NMR spectrum of **4k** in $CDCl_3$

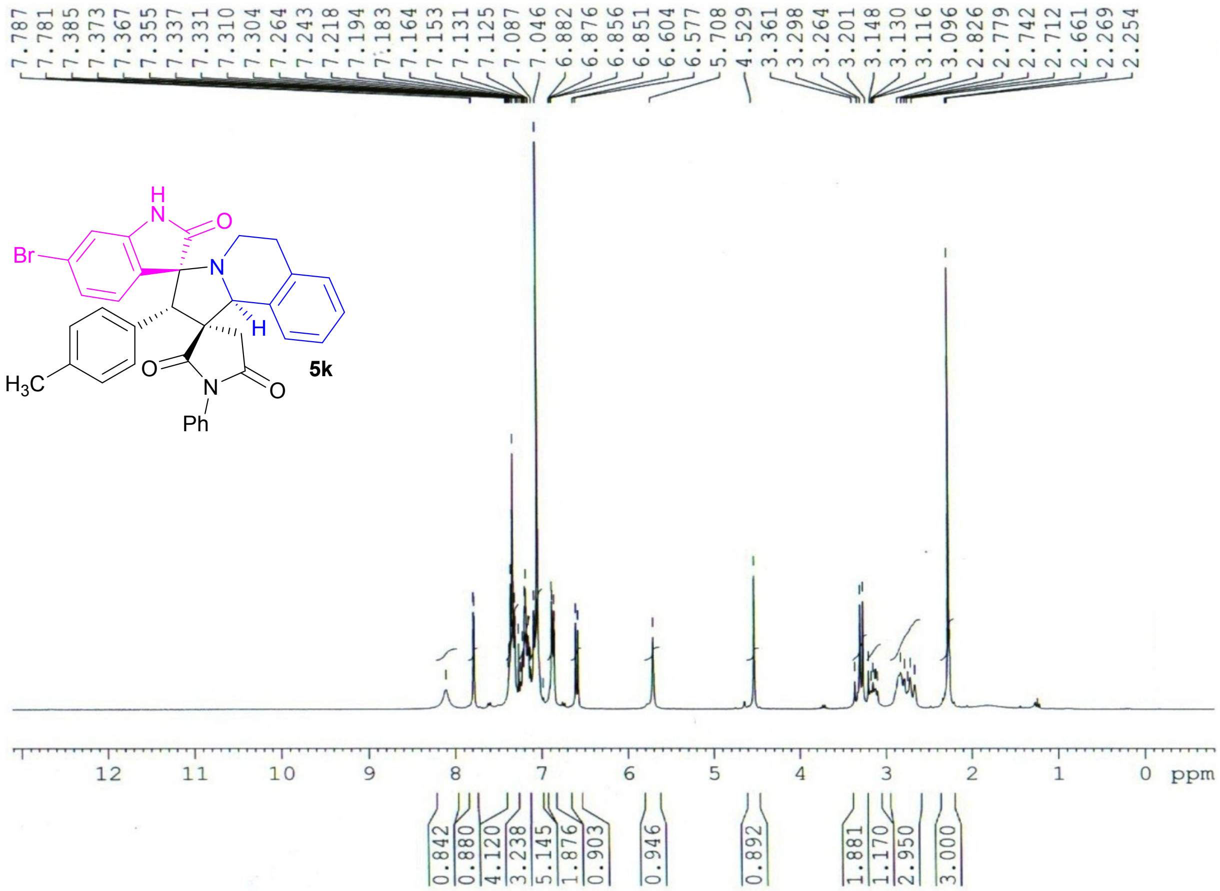

Fig. S35. ^{1}H NMR spectrum of **5k** in $CDCl_3$

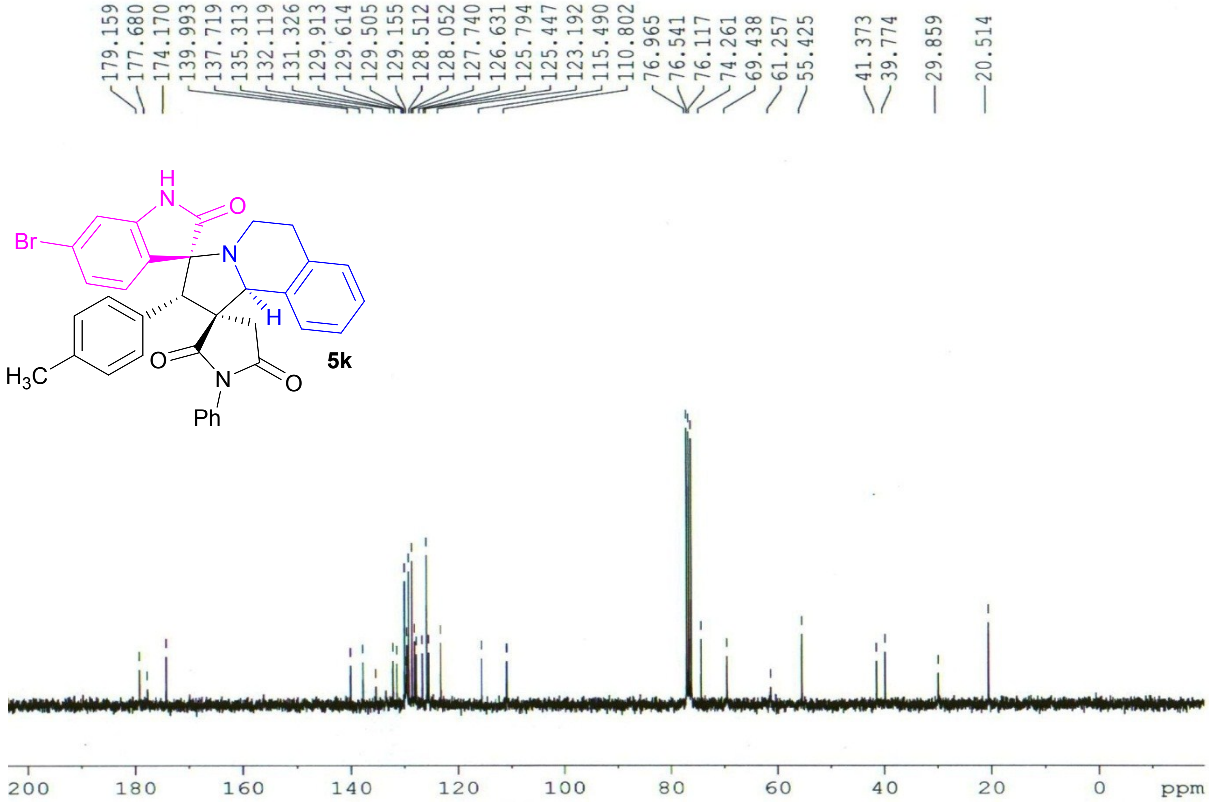

Fig. S36. ^{13}C NMR spectrum of **5k** in $CDCl_3$

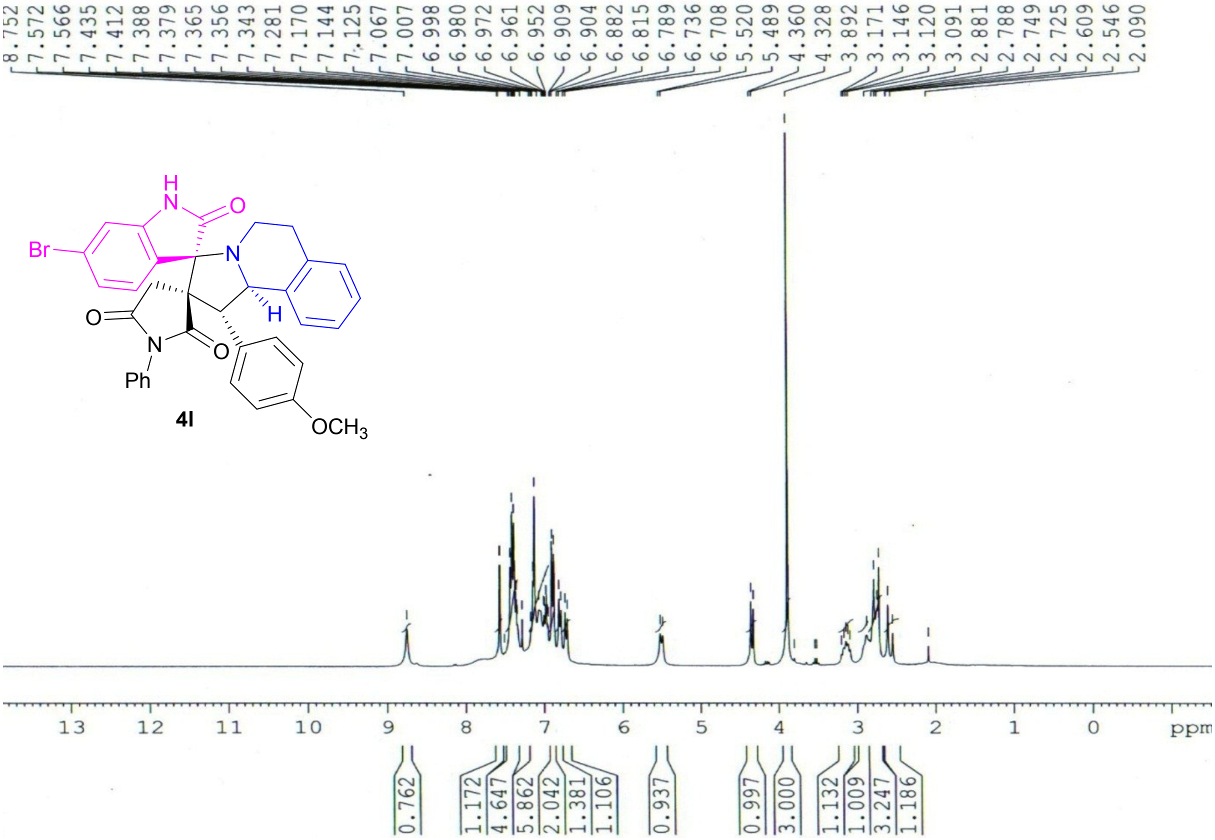

Fig. S37. ^{1}H NMR spectrum of **4l** in $CDCl_3$

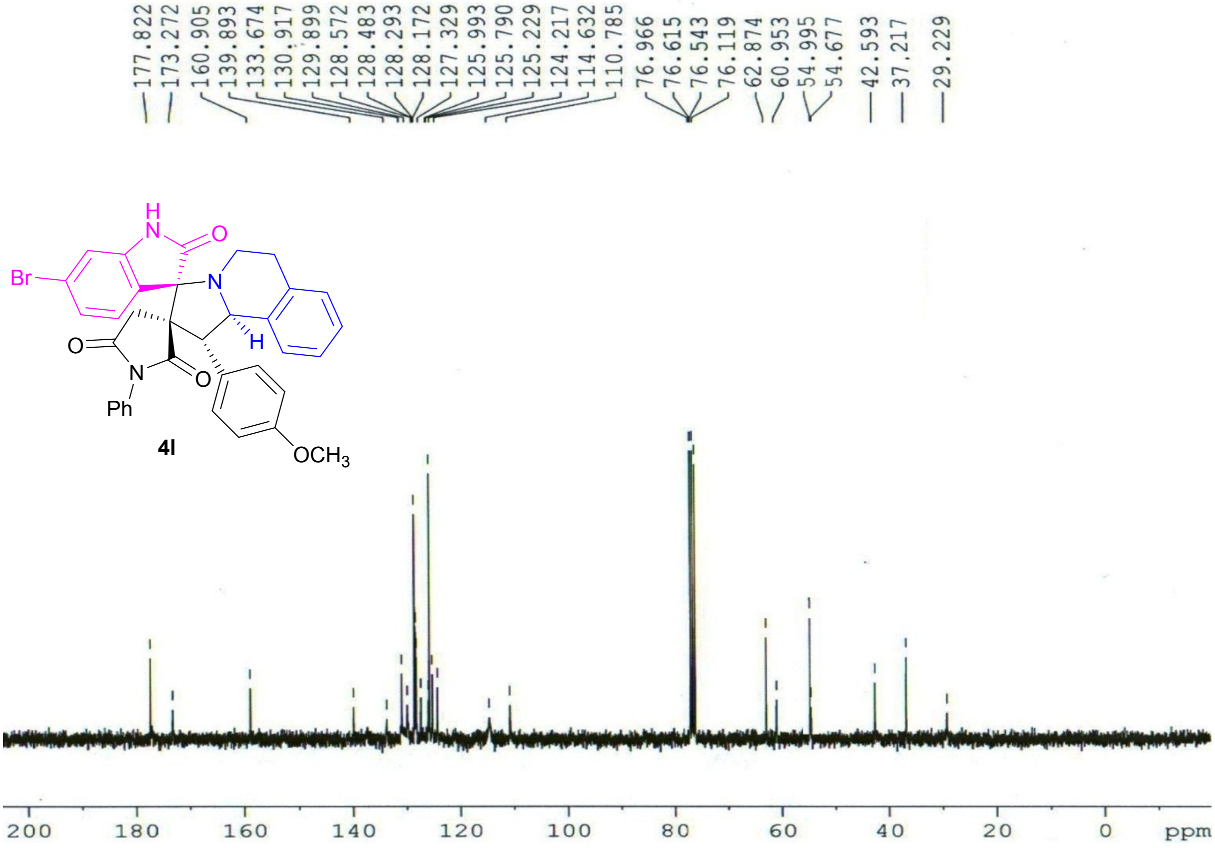

Fig. S38. ^{13}C NMR spectrum of **4l** in $CDCl_3$

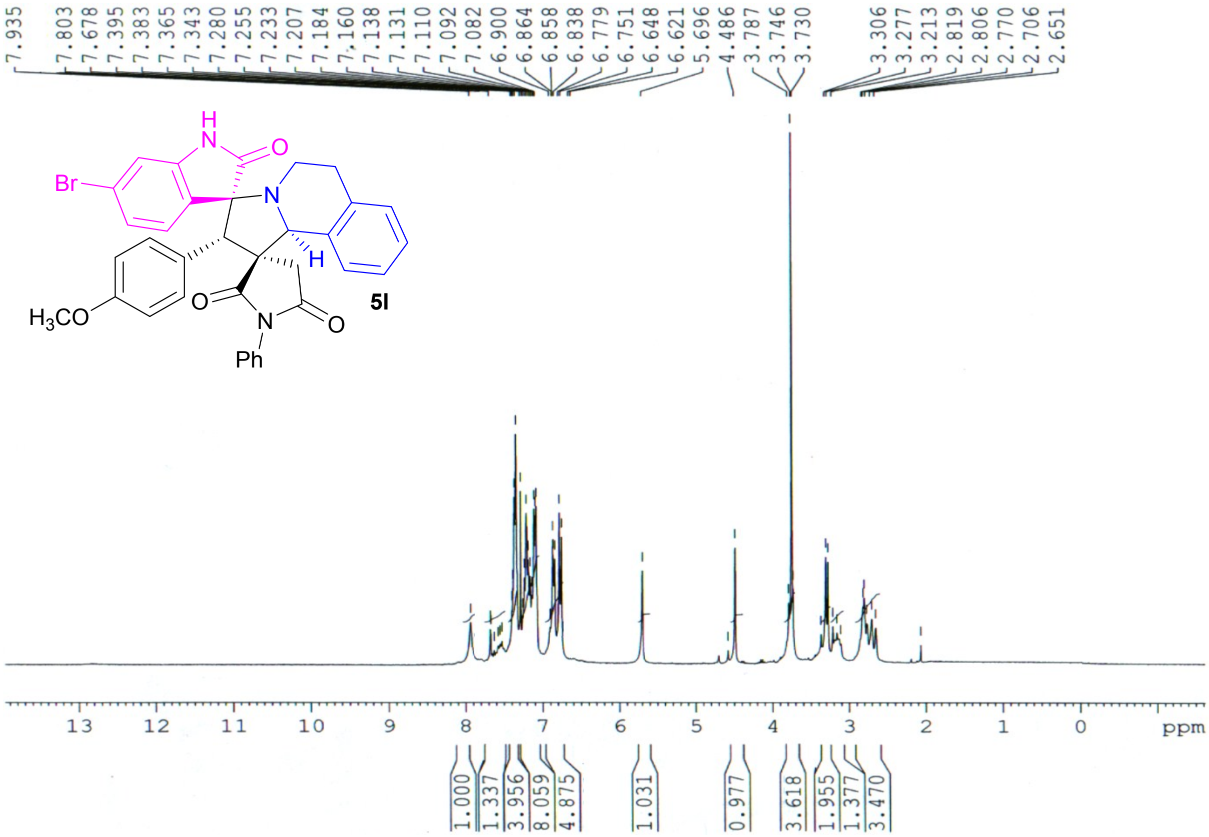

Fig. S39. ^{1}H NMR spectrum of **5l** in $CDCl_3$

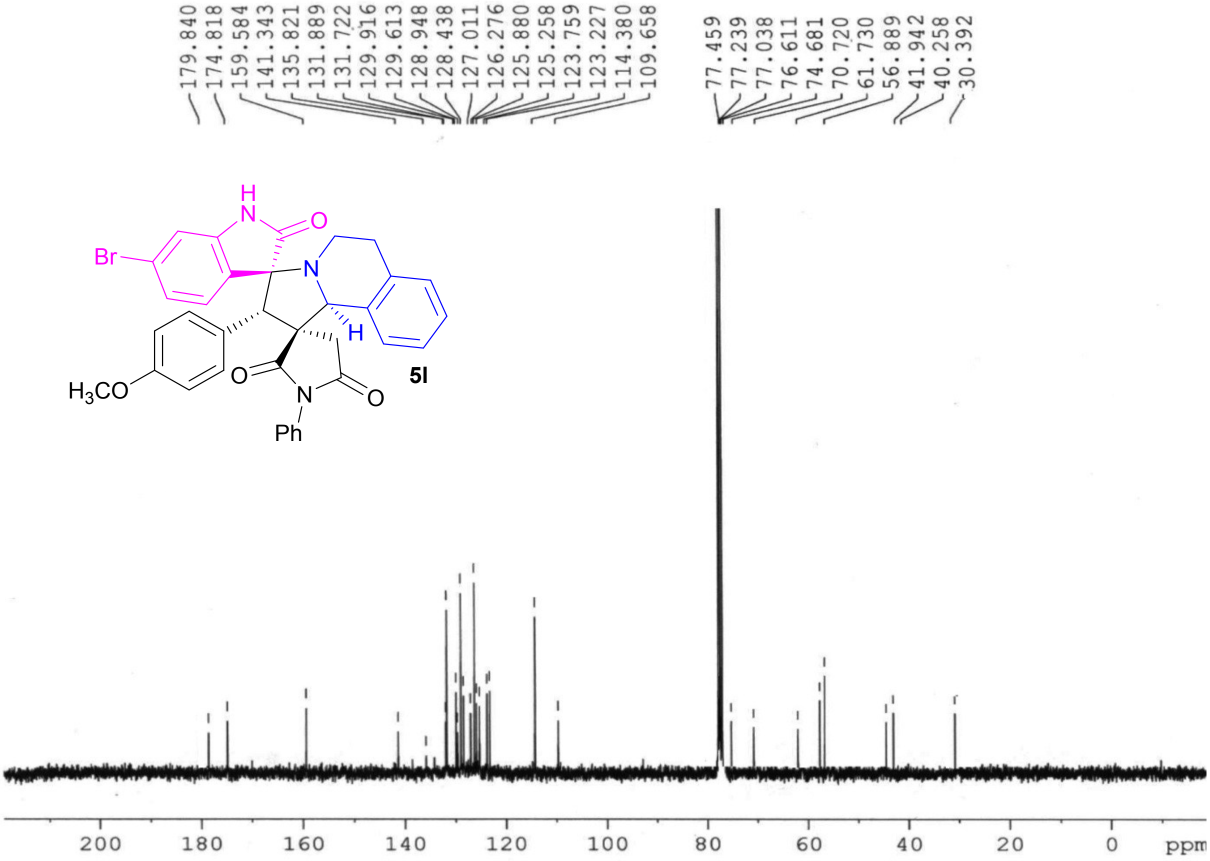

Fig. S40. ^{13}C NMR spectrum of **5l** in $CDCl_3$

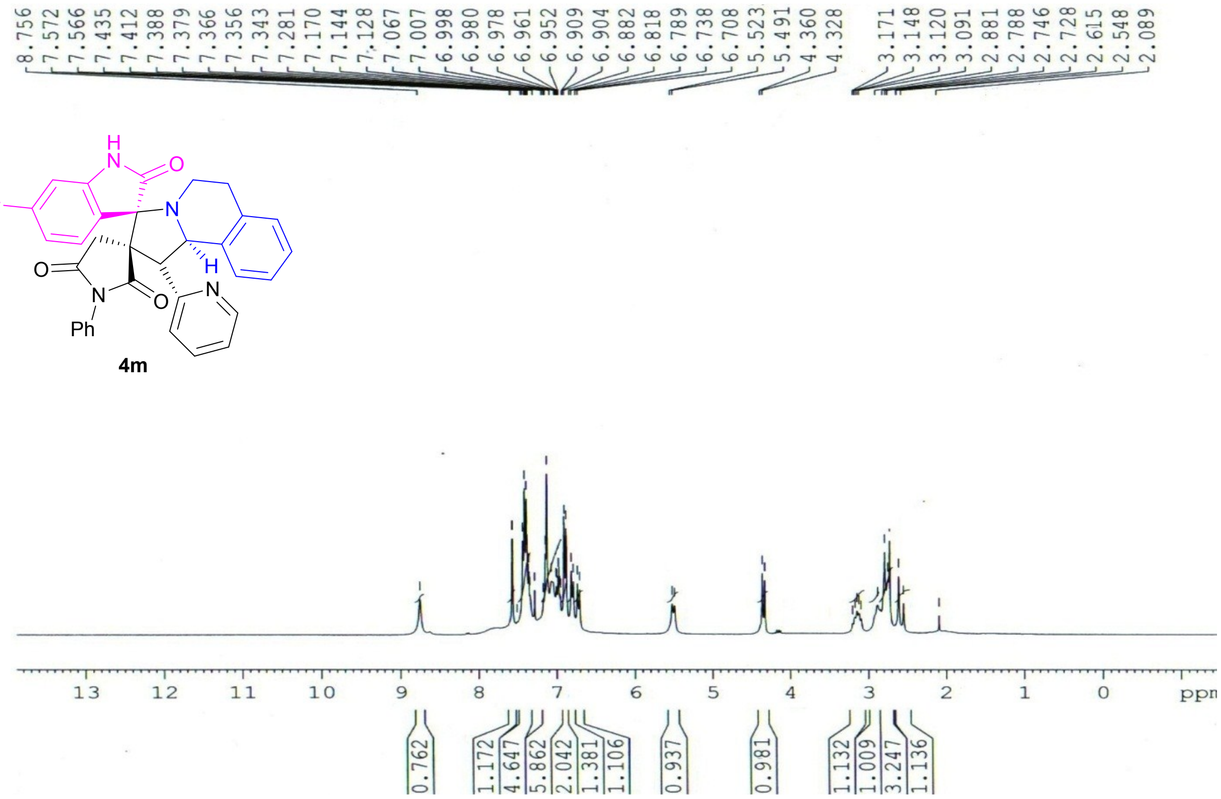

Fig. S41. ^{1}H NMR spectrum of **4m** in $CDCl_3$

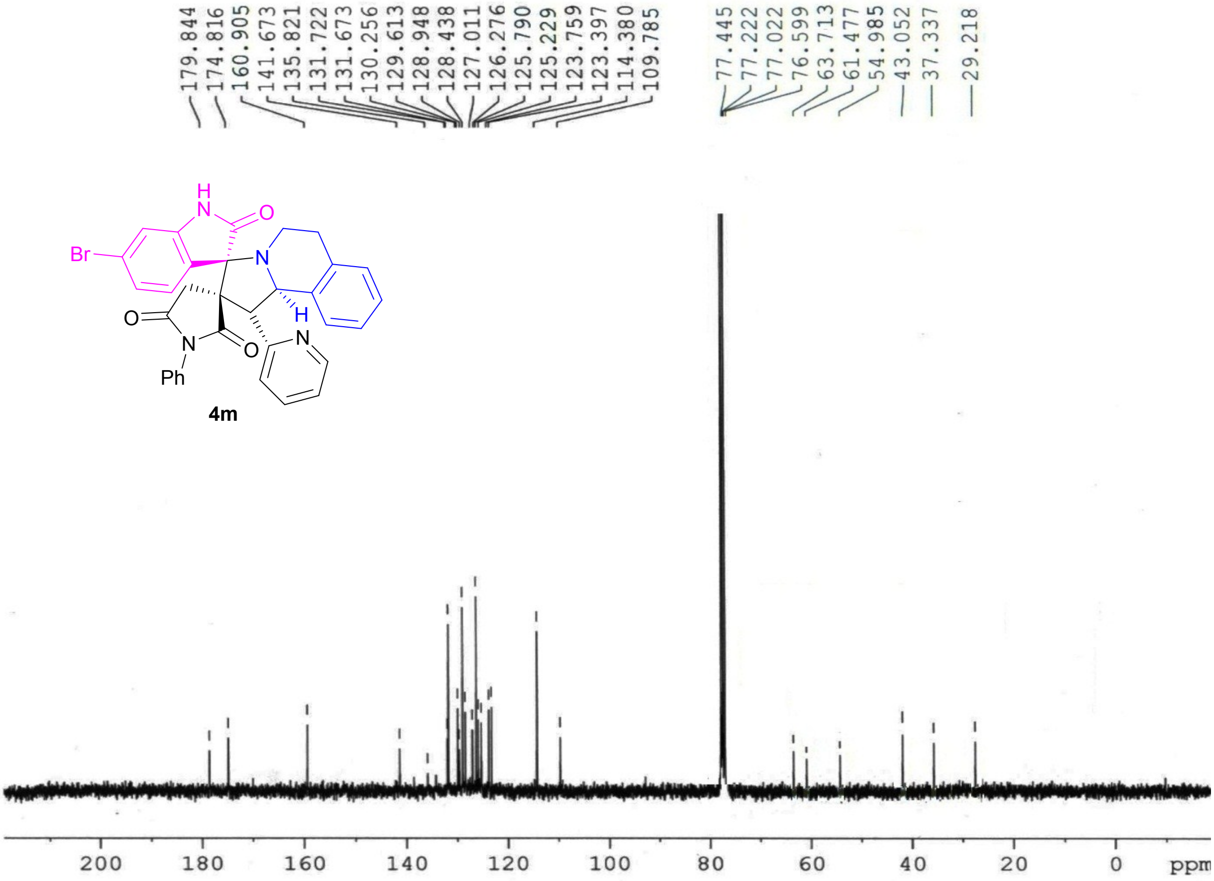

Fig. S42. ^{13}C NMR spectrum of **4m** in $CDCl_3$

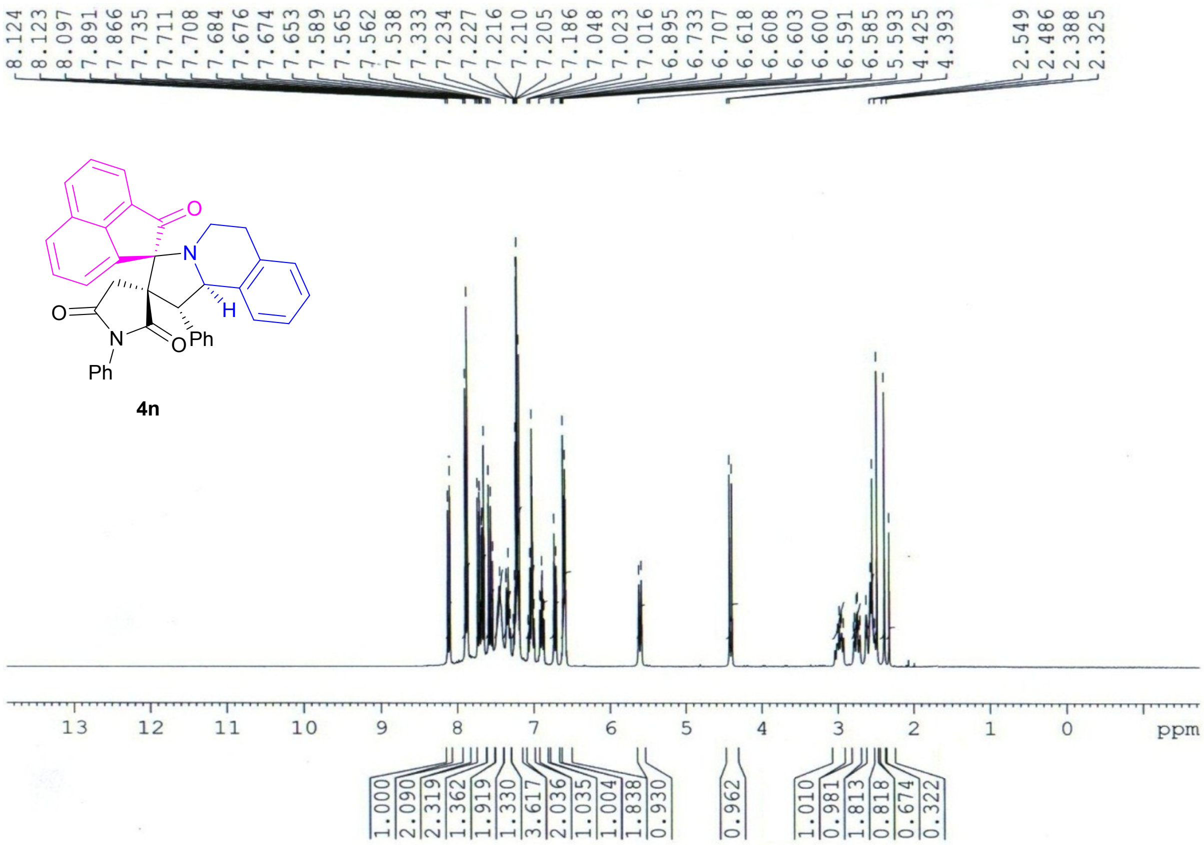

Fig. S43. ^{1}H NMR spectrum of **4n** in $CDCl_3$

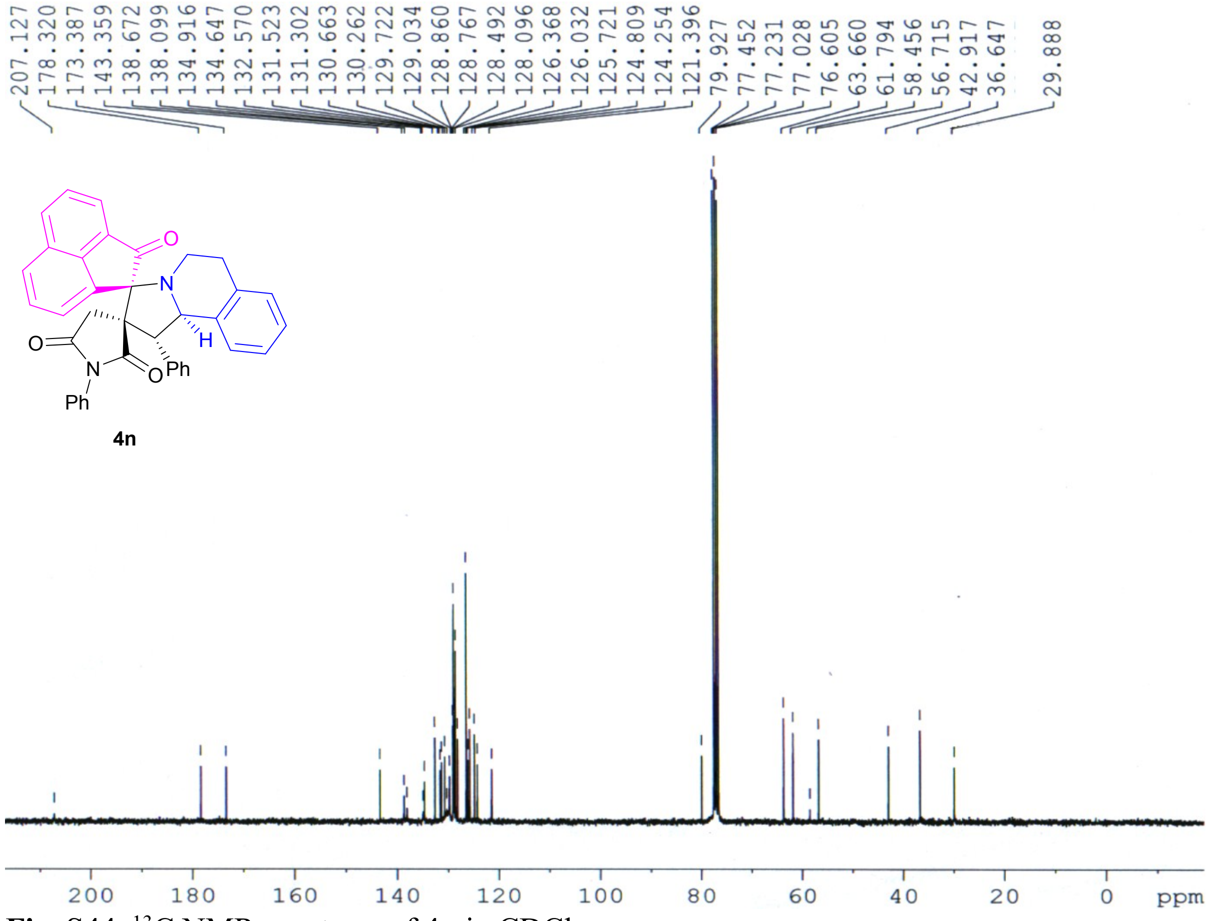

Fig. S44. ^{13}C NMR spectrum of **4n** in $CDCl_3$

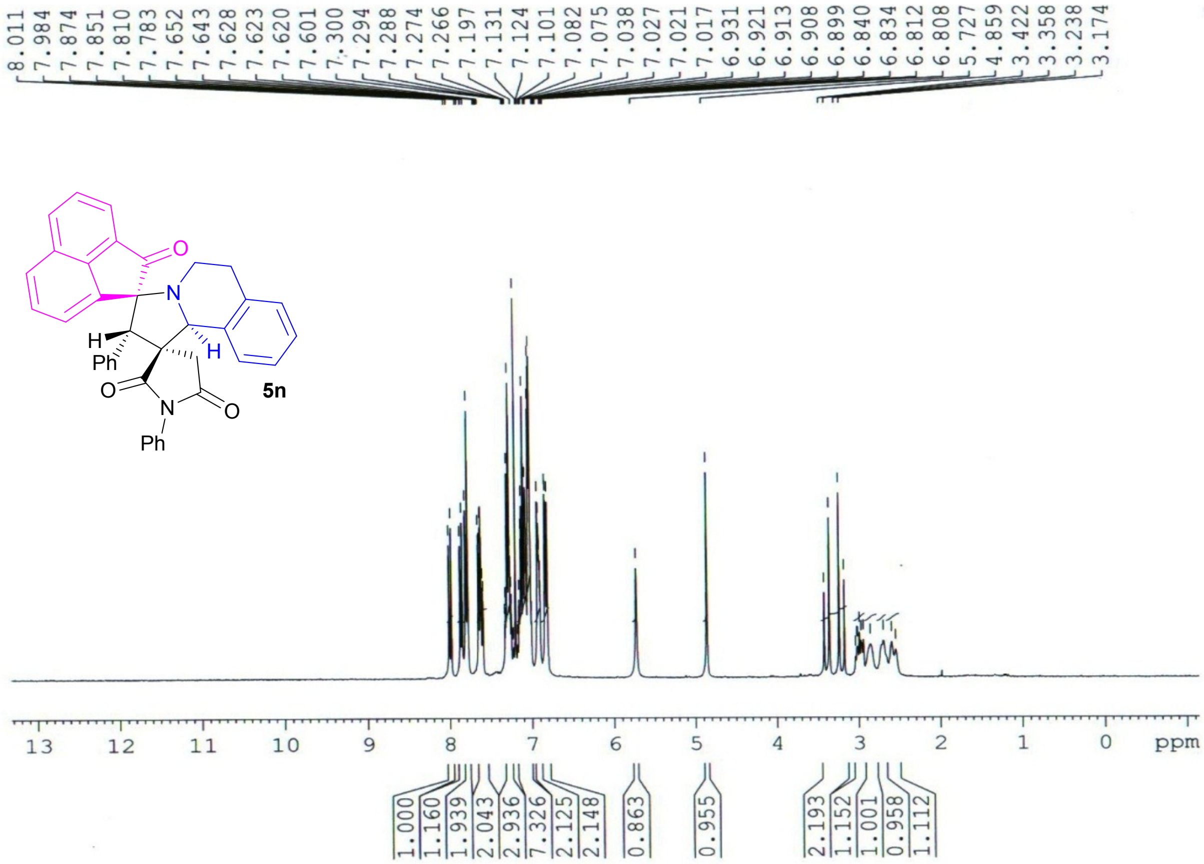

Fig. S45. ^{1}H NMR spectrum of **5n** in $CDCl_3$

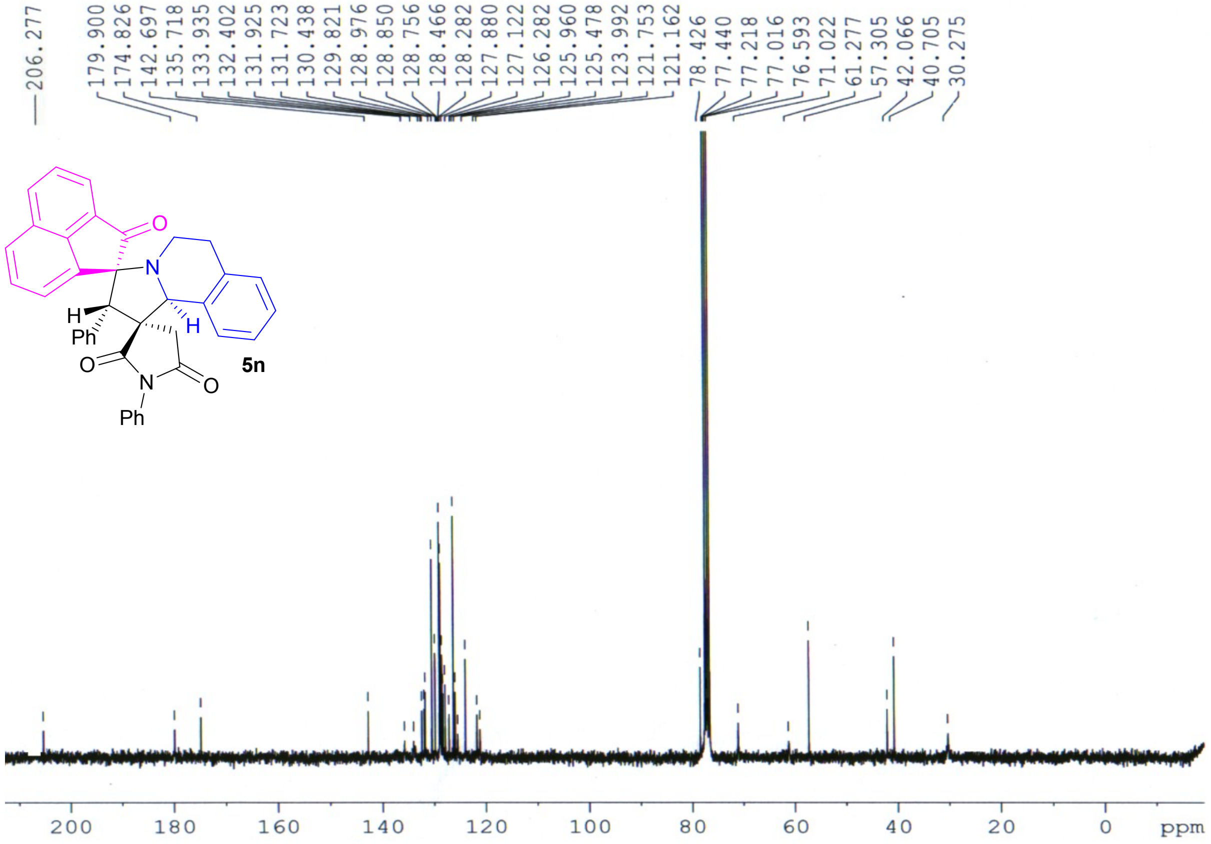

Fig. S46. ^{13}C NMR spectrum of **5n** in $CDCl_3$

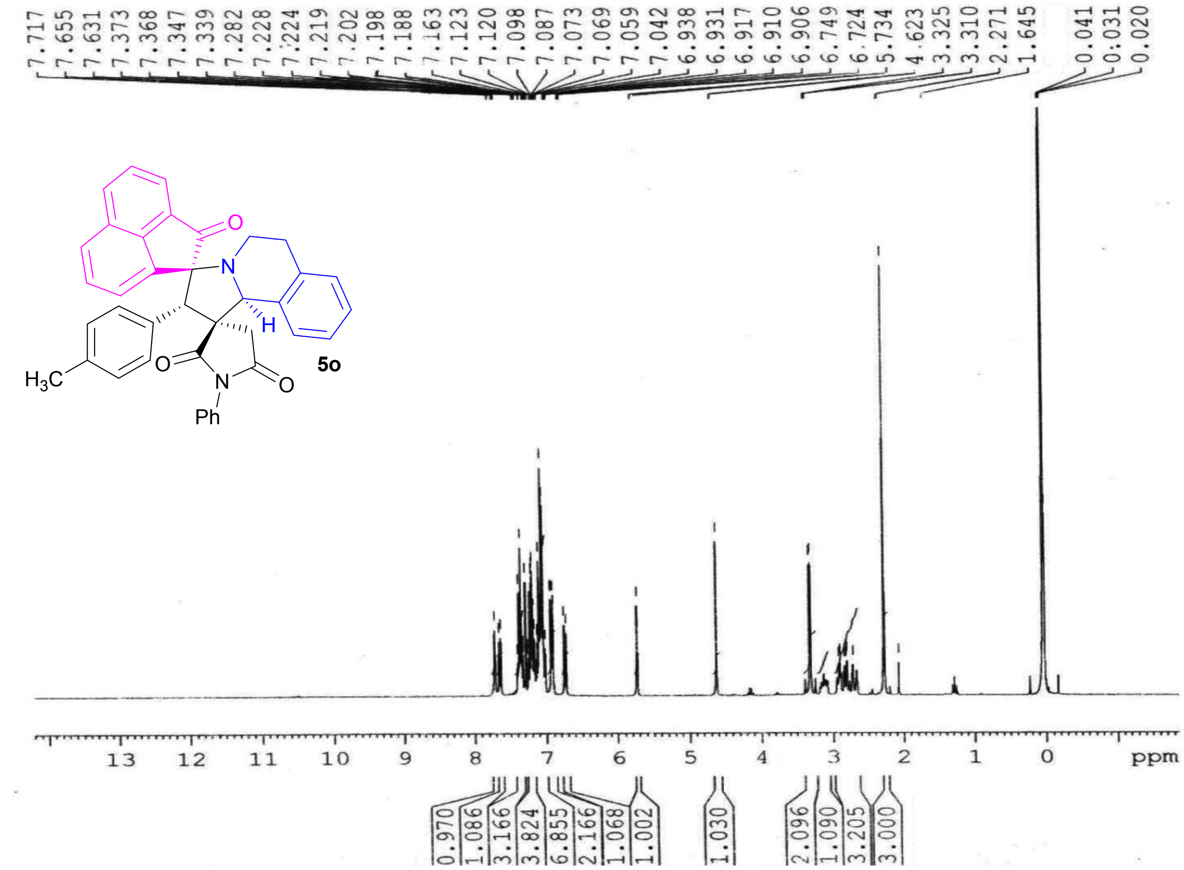

Fig. S47. ^{1}H NMR spectrum of **5o** in $CDCl_3$

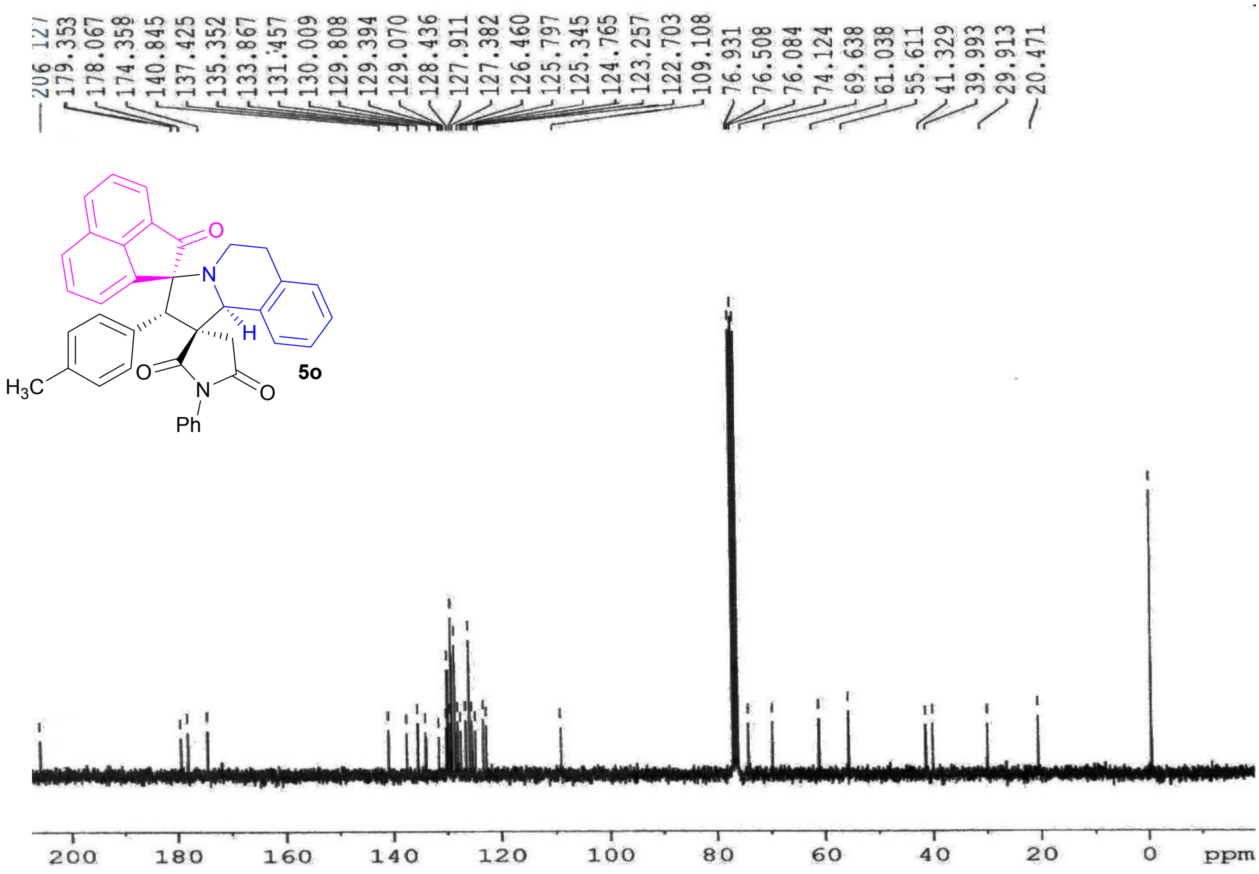

Fig. S48. ^{13}C NMR spectrum of **5o** in $CDCl_3$

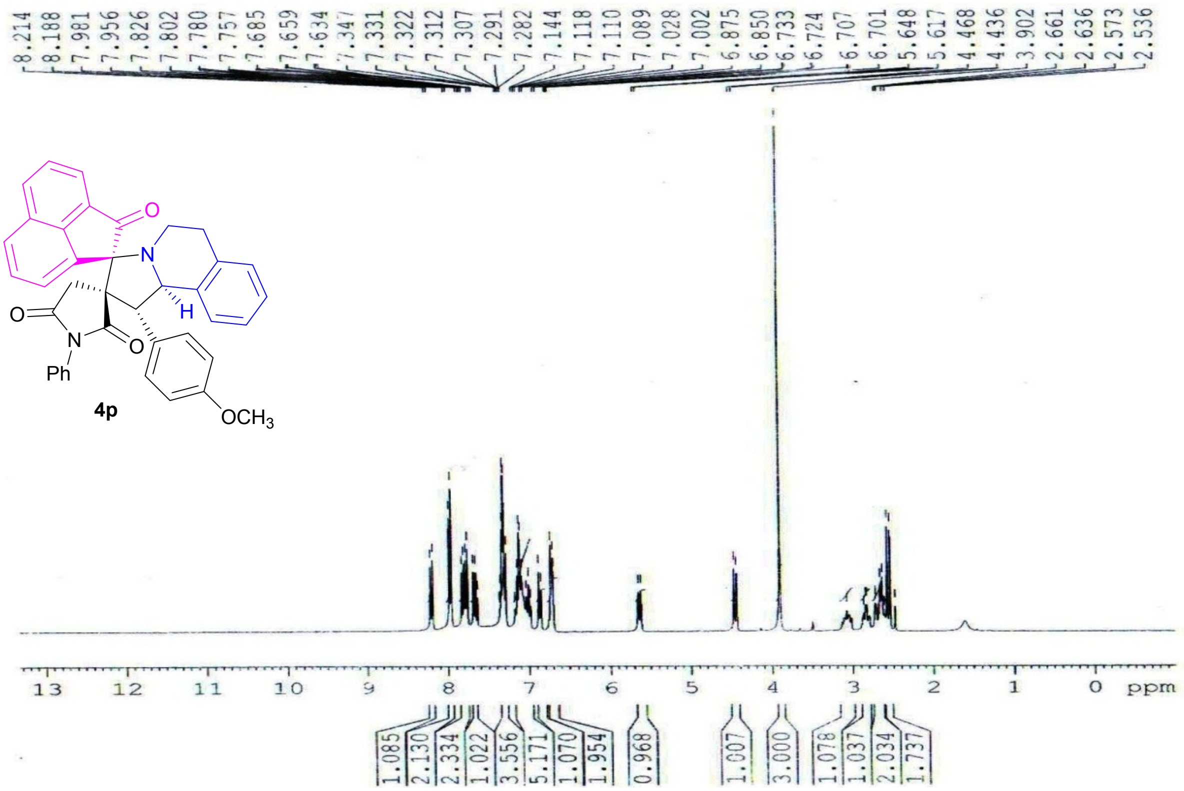

Fig. S49. ^{1}H NMR spectrum of **4p** in $CDCl_3$

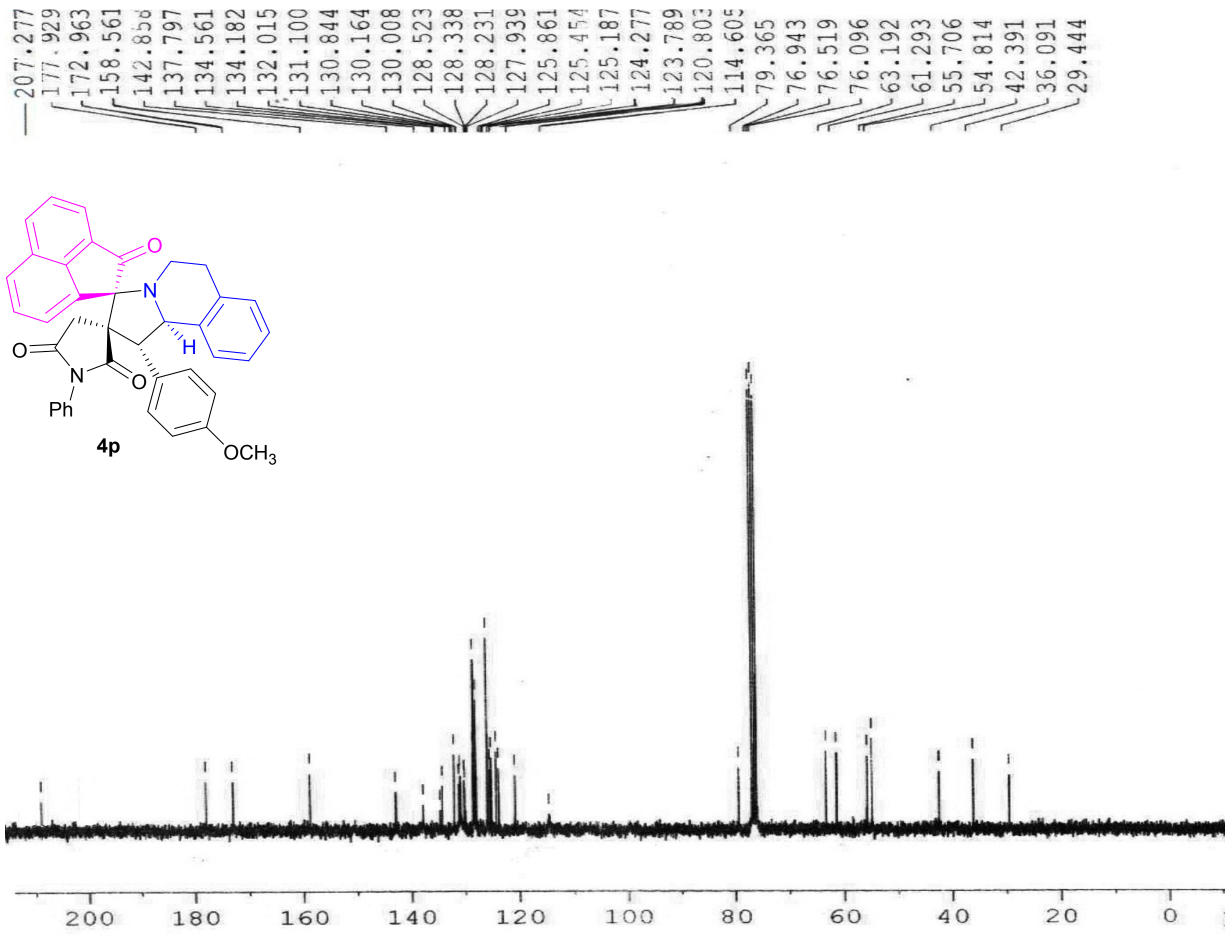

Fig. S50. ^{13}C NMR spectrum of **4p** in $CDCl_3$

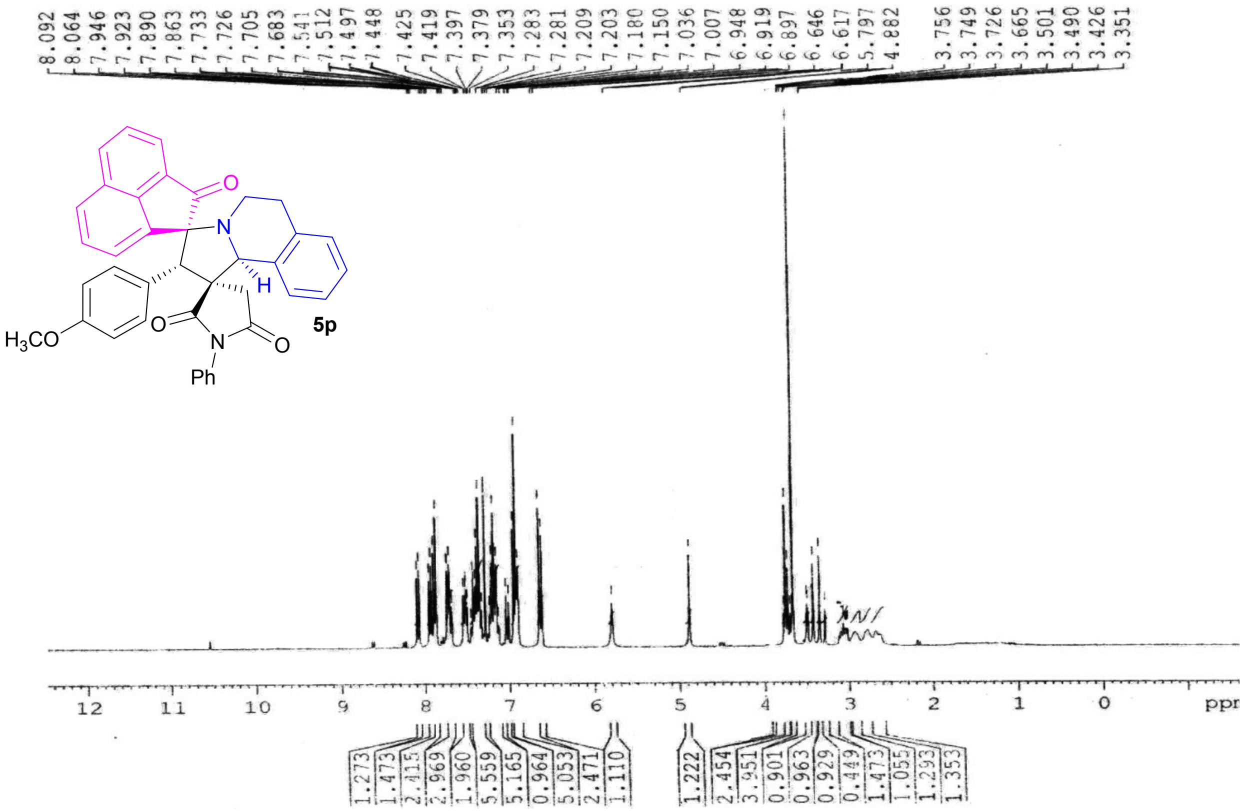

Fig. S51. ^{1}H NMR spectrum of **5p** in $CDCl_3$

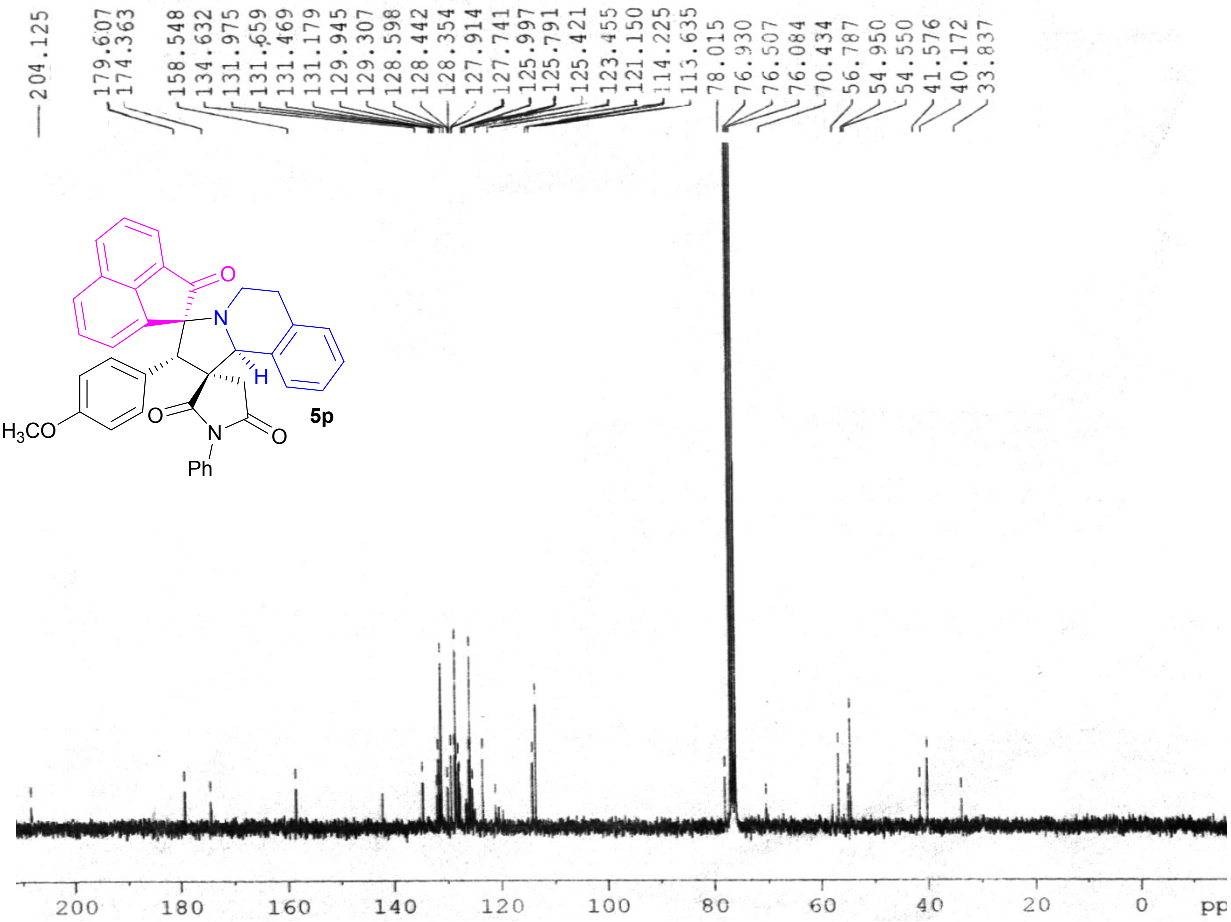

Fig. S52. ^{13}C NMR spectrum of **5p** in $CDCl_3$

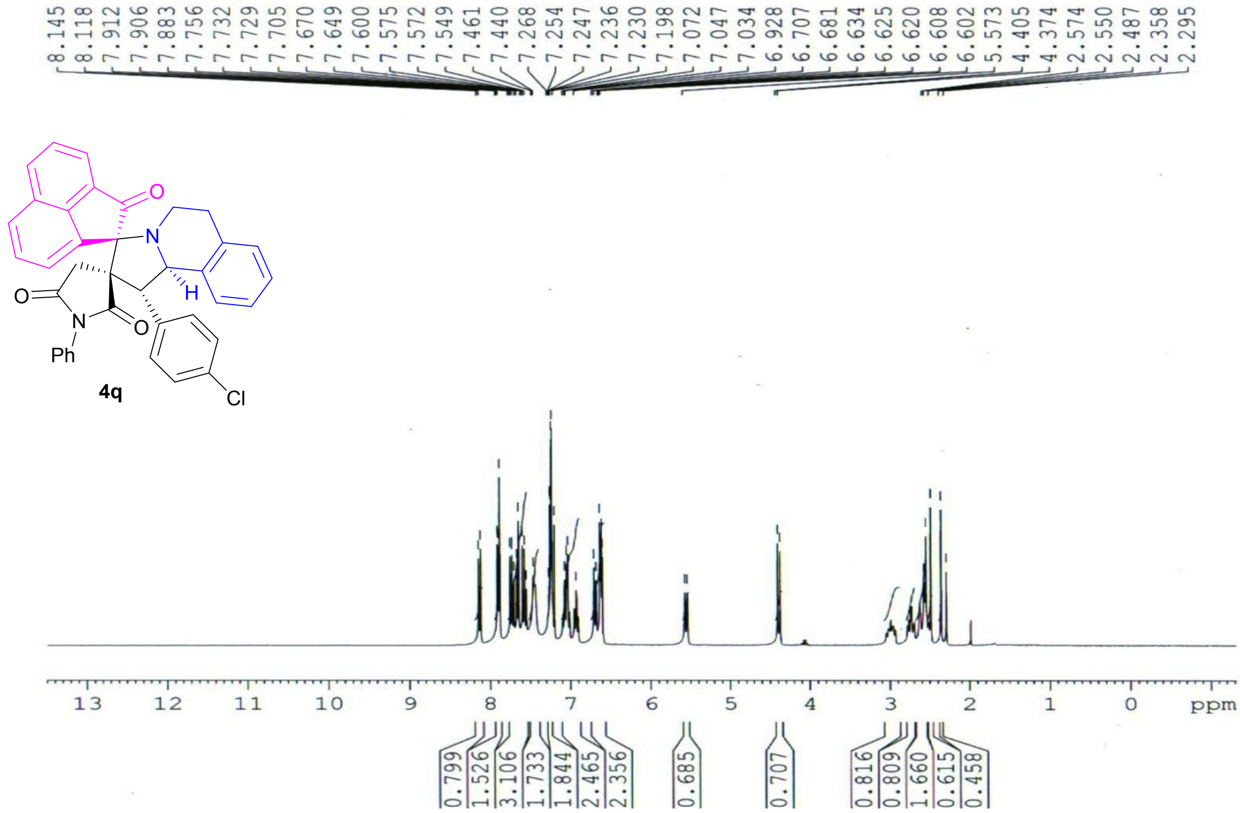

Fig. S53. ^{1}H NMR spectrum of **4q** in $CDCl_3$

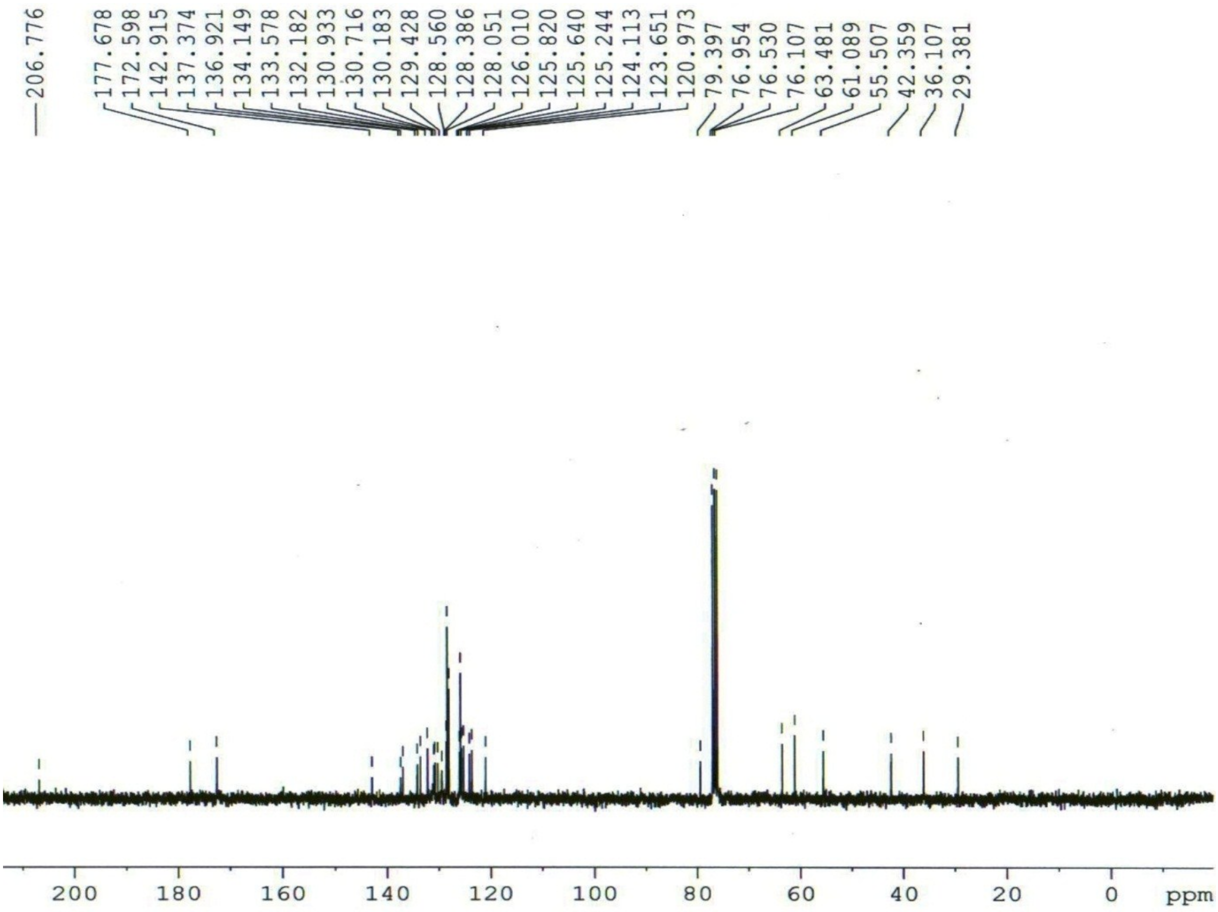

Fig. S54. ^{13}C NMR spectrum of **4q** in $CDCl_3$

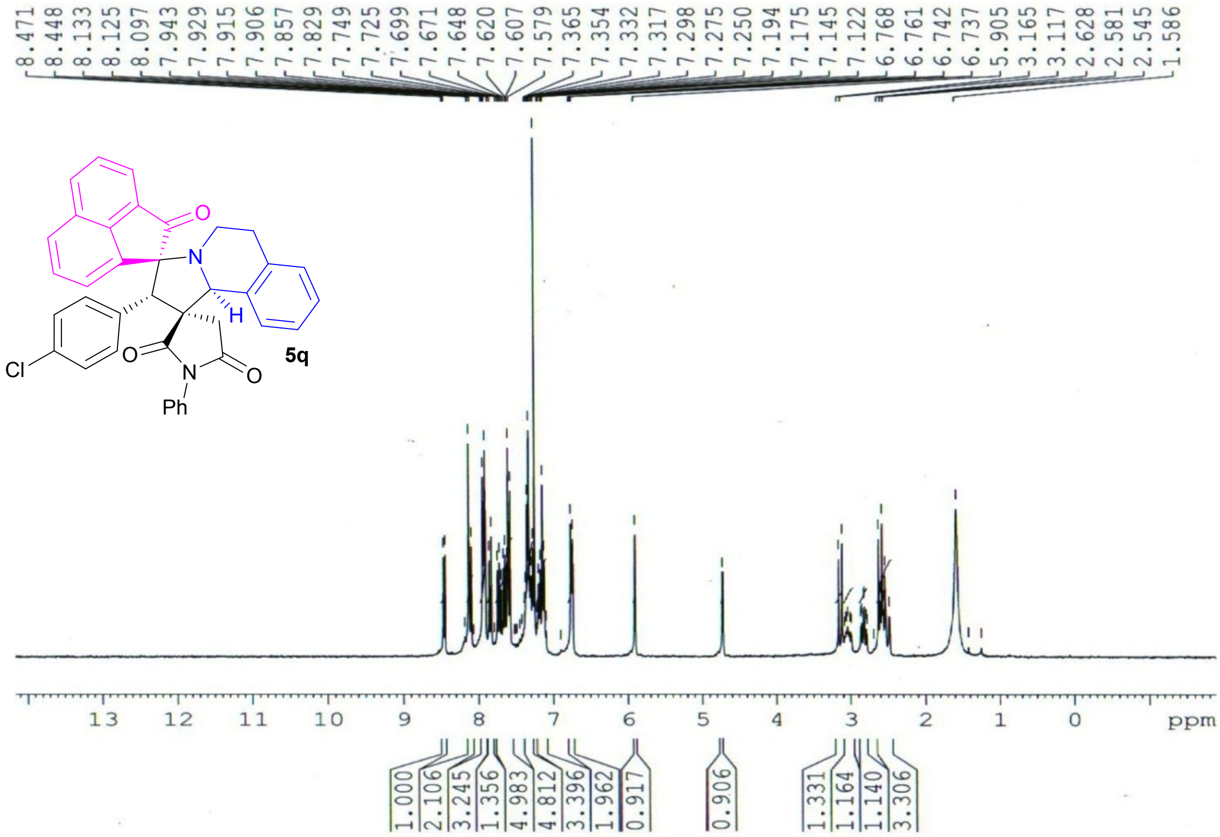

Fig. S55. ^{1}H NMR spectrum of **5q** in $CDCl_3$

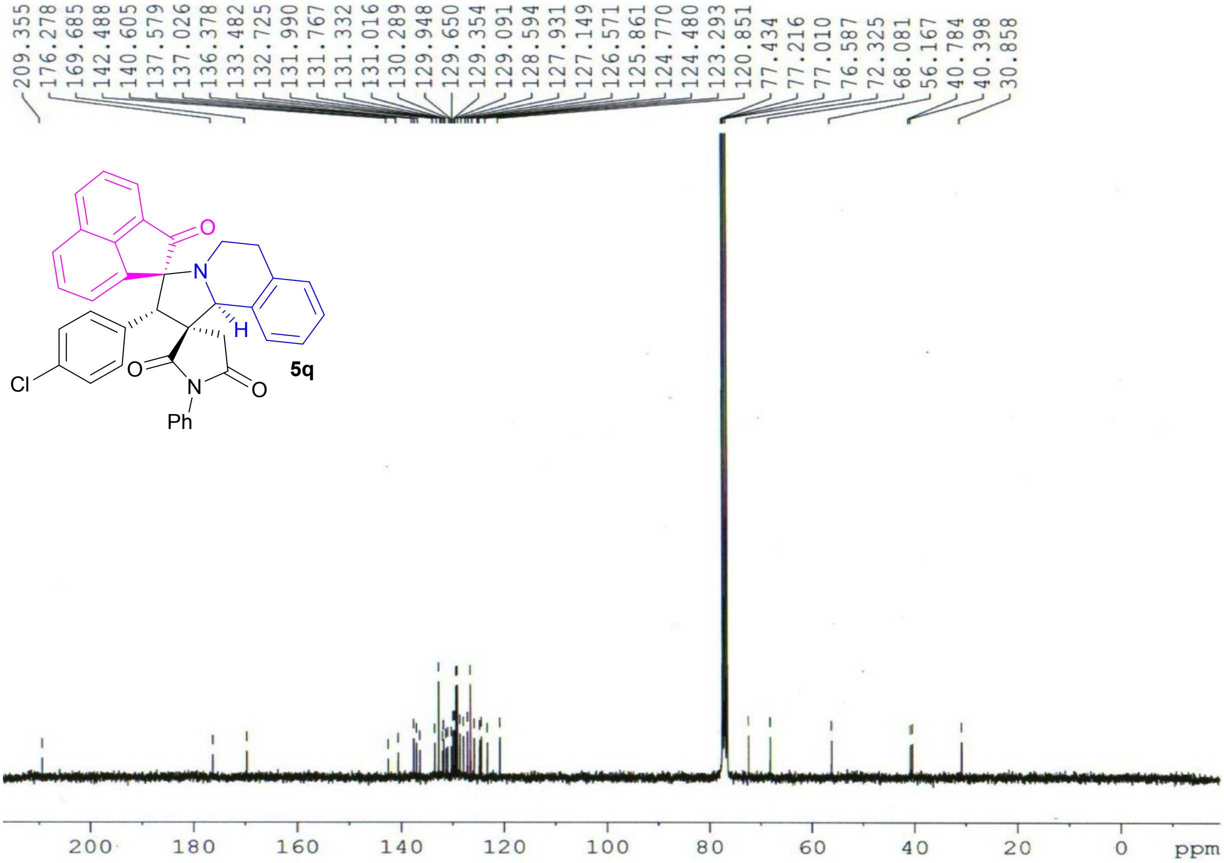

Fig. S56. ^{13}C NMR spectrum of **5q** in $CDCl_3$

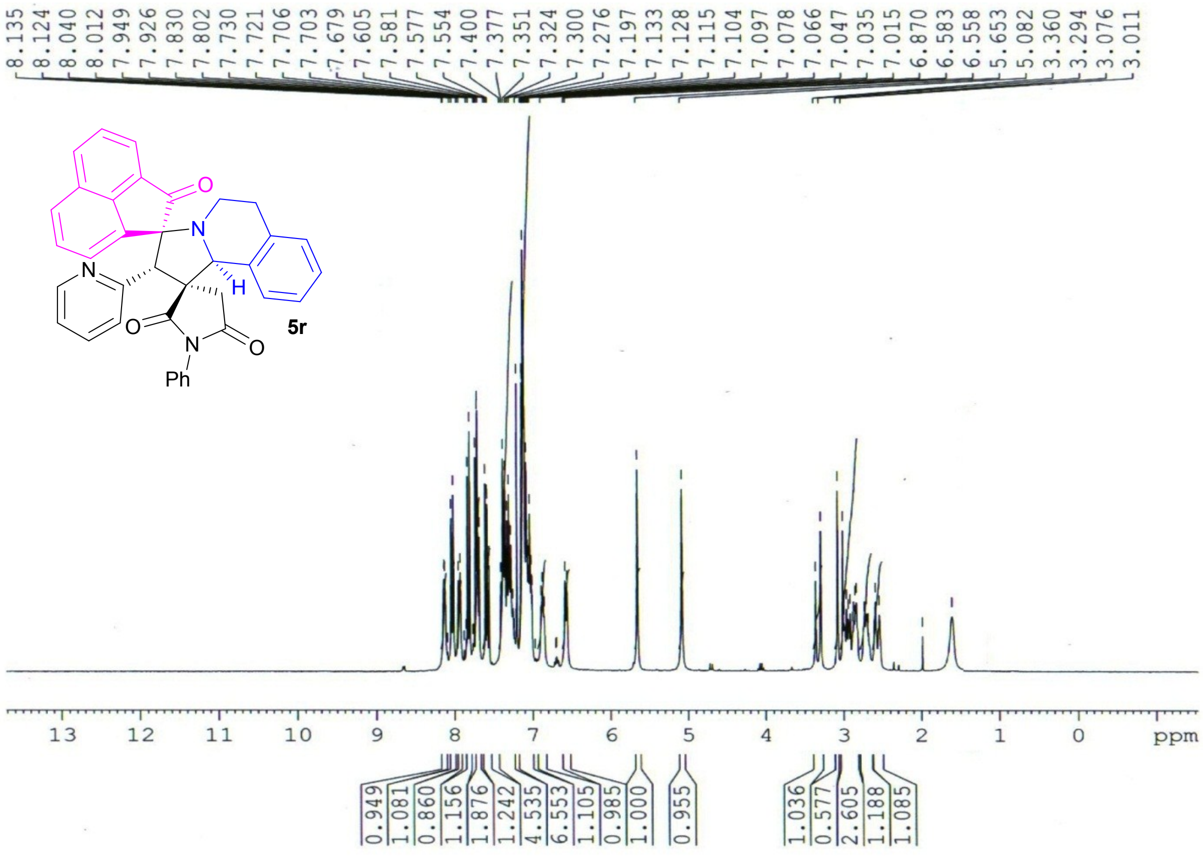

Fig. S57. ^{1}H NMR spectrum of **5r** in $CDCl_3$

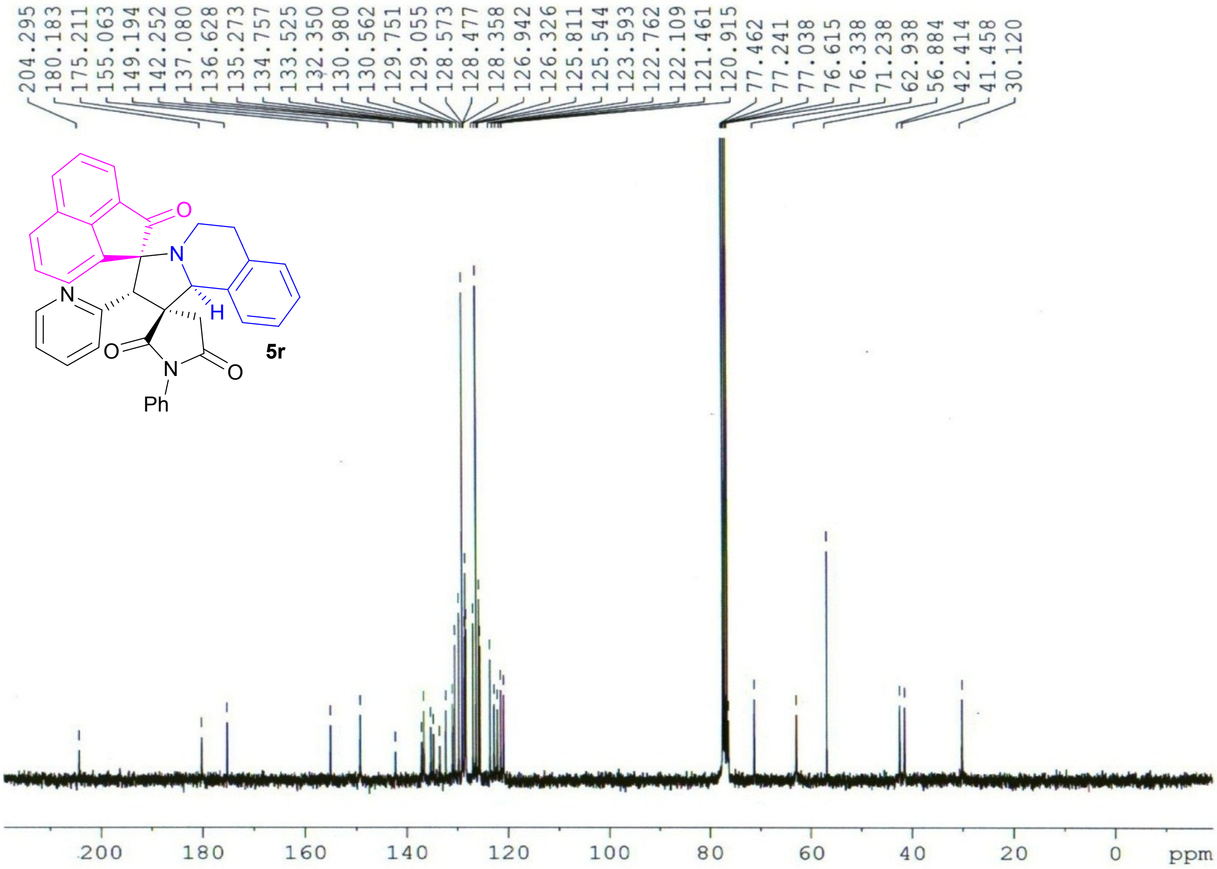

Fig. S58. ^{13}C NMR spectrum of **5r** in $CDCl_3$
